# Epoxy tetrahydrophthalimides as potential bioactive agents: synthesis and computational study

**DOI:** 10.1002/ps.70111

**Published:** 2025-07-30

**Authors:** Kariny Bragato Amorim Torrent, Vitor Cunha Baia, Laisa Samarini Gomes, Eloiza Ribeiro Castro, Vânia Maria Teixeira Carneiro, Elson Santiago Alvarenga

**Affiliations:** ^1^ Department of Chemistry Universidade Federal de Viçosa Viçosa Brazil

**Keywords:** imide, epoxide, herbicide, *in silico*, docking

## Abstract

**BACKGROUND:**

Due to the growing resistance of weeds to known herbicides, the search for new bioactive substances has been increasing in recent years. Imides belong to an important class of chemical compounds known to present various biological activities such as herbicide, fungicide, insecticide, and bactericide. In this study, the synthesis, purification, structural elucidation, and bioassays of four pairs of epoxy tetrahydrophthalimides **15a**–**18a** and **15b**–**18b** were conducted. Additionally *in silico* studies were performed to identify potential biological targets for the synthesized compounds.

**RESULTS:**

The target compounds were prepared using a four‐step synthetic route that starts with a microwave‐assisted Diels–Alder reaction between maleic anhydride and isoprene. All synthesized compounds had their phytotoxicity evaluated using germination tests in Petri dishes against *Lactuca sativa*, *Cucumis sativus*, *Sorghum bicolor* and *Bidens pilosa*. At concentrations of 500 and 300 μm substance **16a** presented inhibition of 70% and 66%, respectively, of the aerial parts of sorghum plants, which is higher than that observed for the commercial herbicide *S*‐metolachlor. Molecular docking studies were performed for compounds **15a**, **15b**, **17a**, and **17b**, indicating that they form complexes with the mitogen‐activated protein kinase 5R92, which shares similar amino acid sequences with those found in plants.

**CONCLUSION:**

All substances caused inhibition or stimulation of seed growth compared to the control. Some substances caused plant growth inhibition superior or equivalent to the commercial herbicide, denoting these imides for the development of new agrochemicals. *In silico* studies suggest that mitogen‐activated protein kinase may be the target of these compounds. © 2025 The Author(s). *Pest Management Science* published by John Wiley & Sons Ltd on behalf of Society of Chemical Industry.

## Graphical abstract

Epoxy tetrahydrophthalimides were synthesized and evaluated for phytotoxic activity. Some compounds showed greater inhibition than *S*‐metolachlor against *Sorghum bicolor*. Docking studies indicated interaction with mitogen‐activated protein kinase, suggesting it as a potential target. These findings support the potential of imides as leads for new herbicidal agents.

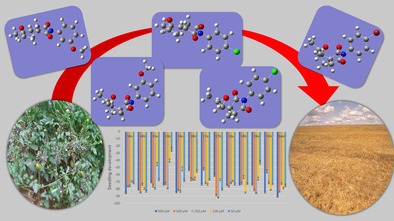

## 1 INTRODUCTION

Herbicides are bioactive substances that provide effective and economical control of weeds, increasing crop productivity. In recent decades, herbicides have become the most widely used agrochemical products in the world.^1^, ^2^ However, over time, and due to overuse of certain herbicides, weeds have evolved and developed some resistance to these bioactive substances.^3^, ^4^, ^5^ In fact, in South America 143 cases of resistance have been reported, 50 of them in Brazil.^6^ Therefore, the search for new bioactive substances is increasing.

Plant‐based bioactive substances play an important role in the development of more selective and effective pesticides. These natural products offer a wide variety of biological activities that can be exploited to control crop pests, providing a promising path toward safer and more environmentally responsible agricultural practices.^7^, ^8^, ^9^, ^10^

One strategy for designing and developing bioactive substances involves molecular hybridization, which is based on combining different molecules to produce new compounds with enhanced biological potential.^11^, ^12^ Substances containing an amide subunit in their structure belong to an important class of organic compounds separated into subclasses, such as: succinimides, maleimides, glutarimides, phthalimides and naphthalimides. Imide derivatives have attracted the attention of researchers because they are electrically neutral compounds of hydrophobic nature, able to cross biological membranes resulting in high biological potential. They are useful in the synthesis of pharmaceutical and agrochemical products and serve as a structural platform for obtaining a variety of bioactive compounds.^13^, ^14^, ^15^, ^16^, ^17^

Two recent studies highlight the potential of imide‐ and amide‐based derivatives as sustainable alternatives for weed control. Mejías *et al*.^18^ developed and characterized a core‐shell formulation composed of Pluronic F‐127 nanoparticles embedded in a poly(vinyl alcohol) polymeric matrix to encapsulate the strigolactone mimic PL01, which contains an imide functional group in its structure. This nanoformulation significantly increased the compound's water solubility while preserving its ability to induce suicidal germination of parasitic weed seeds such as *Phelipanche aegyptiaca* and *Phelipanche ramosa*. Similarly, Scavo *et al*.^19^ demonstrated through field trials that an aminophenoxazinone‐based mimic containing an amide group, nanoencapsulated in organic particles, effectively controlled weeds in durum wheat crops. The bioherbicide showed efficacy comparable to or greater than that of commercial herbicides and exhibited low environmental toxicity. Together, these findings underscore the promising role of imide‐ and amide‐functionalized compounds, combined with nanotechnology, in the development of more effective and environmentally safe bioherbicides.

**Figure 1:**
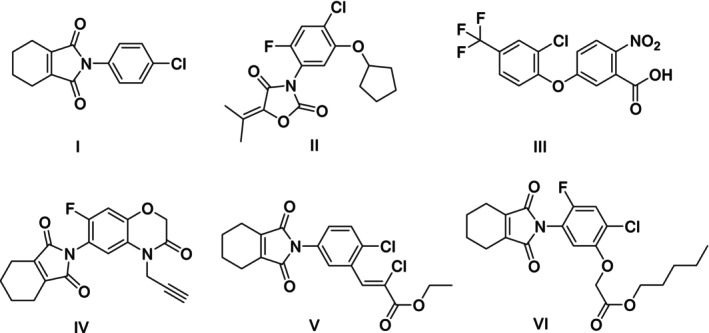
Commercial herbicides chlorphthalim (**I**), pentoxazone (**II**), acifluorfen (**III**), flumioxazin (**IV**), cinidon‐ethyl (**V**), and flumichlorac‐pentyl (**VI**).

Imides marketed by agrochemical industries as herbicides are shown in Fig. 1. These substances inhibit the enzyme protoporphyrinogen‐IX oxidase (PPO) which acts in the synthesis of chlorophyll, and can effectively control long leaf weeds, with a small dosage.^20^, ^21^, ^22^, ^23^

Inhibition of PPO prevents the conversion of protoporphyrinogen‐IX to protoporphyrin‐IX, leading to the accumulation and non‐enzymatic oxidation of protoporphyrinogen‐IX. This accumulation causes damage to cell membranes, resulting in rapid necrosis of weed tissues. Due to their swift and lethal action, PPO inhibitor herbicides are widely used, particularly in situations requiring quick and effective control of weeds resistant to other modes of action.^24^, ^25^

In recent years, our research group has been working on synthesizing amides with potential biological activities, including insecticides^26^, ^27^ and herbicides. Chiral amides have shown insecticidal activity against the stored grain pest *Rhyzopertha dominica*,^28^ while lumissantonin amides have presented phytotoxic selectivity against *Sorghum bicolor*.^29^ Amides analogous to piperine were synthesized and tested for toxicity against the tomato pinworm, *Tuta absoluta*, and its predators, the ant *Solenopsis saevissima* and the wasp *Polybia ignobilis*.^30^ Aromatic amides, derived from sorbic acid and hexanoic anhydride, showed herbicidal activity in seed germination tests on three dicotyledonous species and one monocotyledonous plant, and these compounds appear to inhibit histone deacetylase in plants, according to *in silico* studies.^31^ Novel potential herbicides were also prepared from sorbic acid, resulting in eight amides with bioactivity in sorghum, onion, cucumber, lettuce and beggartick seeds. The protein target of the most active substance in plants was identified *in silico*.^32^ *N*‐Phenylnorbornenosuccinic acids and *N*‐phenylnorbornenesuccinimide derivatives were prepared using Diels–Alder reactions and exhibited significant inhibitory activity on five plant species tested. Molecular docking studies indicated the possible enzymatic target of one of the compounds.^33^

In our previous study, we detailed the synthesis, identification, and biological activity of epoxy derivatives of 5‐methylhexahydroisoindole‐1,3‐dione, as well as the biological evaluation of these compounds.^34^ This work reports the synthesis of eight new epoxy tetrahydrophthalimides in four steps from the readily available maleic anhydride, and isoprene. The herbicidal activities of these imides were evaluated using seeds of the monocotyledonous plant *Sorghum bicolor* and seeds of the dicotyledonous plants *Lactuca sativa*, *Cucumis sativus*, and *Bidens pilosa*. *In silico* study was conducted to find potential enzymatic targets for the synthesized compounds.

## 2 MATERIALS AND METHODS

### 2.1 General procedures

The melting points were obtained in MQAPF‐301 melting point apparatus and were not corrected. Infrared (IR) spectra were acquired using Varian 660‐IR spectrophotometer (equipped with GLADI‐ATR; Agilent, Santa Clara, CA, USA) using the attenuated total reflectance (ATR) method. Nuclear magnetic resonance (NMR) spectra were obtained on a Bruker 400 MHz equipment using deuterated chloroform (CDCl_3_) as solvent. Proton (^1^H)‐NMR chemical shifts were reported using residual chloroform (CHCl_3_) signal (*δ* = 7.27 ppm) as reference. Carbon‐13 (^13^C)‐NMR chemical shifts were recorded using CDCl_3_ (*δ* = 77.0 ppm) as reference. Mass spectra were obtained using Shimadzu (Kyoto, Japan) GC–MS‐QP5050A and GC–MS‐QP2010 Ultra after 70 eV electron impact (EI) ionization. The ^1^H‐ and ^13^C‐NMR, IR and mass spectra of the synthesized target compounds **15a–18a** and **15b–18b** can be found in Supporting Information (Figs S5–S46).

### 2.2 Synthetic procedures

#### 2.2.1 Procedure for the synthesis of carbamoyl carboxylic acids **3**–**6**

Maleic anhydride **1** (9.8 g, 0.10 mol) and isoprene (6.8 g, 0.10 mol) were irradiated in a microwave reactor for 5 min. After this period, the sealed tube was cooled to room temperature providing a white solid that was washed with hexane to afford the tetrahydrophthalic anhydride **2**, in 99% yield. Then 3.0 mmol (0.50 g) of the anhydride **2** was transferred to a round bottomed flask and suspended in 3.0 mL of anhydrous dichloromethane (DCM). The mixture was stirred until complete solubilization. Then, 3.0 mmol of the corresponding amine was added under magnetic stirring. The reactions were instantaneous. The solvent was removed under reduced pressure and the residue was recrystallized from a mixture of hexane and DCM, providing the desired carbamoyl carboxylic acids **3**–**6**, in yields between 65% and 99%.

#### 2.2.2 Procedure for the synthesis of tetrahydrophthalimides **11**–**14**

To a solution of 0.50 g of the product of the previous step (compounds **3**–**6**) in methanol (30 mL) concentrated sulfuric acid (2.0 mL) was added dropwise and the resulting mixture was stirred for 1 h at room temperature. The reaction mixture was transferred to a separatory funnel, dissolved with DCM (30 mL) and washed with saturated aqueous solution of sodium bicarbonate (3 × 30 mL). The organic layer was dried over anhydrous magnesium sulfate, filtered and the solvent evaporated under reduced pressure, to afford a mixture of esters **7**–**10** and tetrahydrophthalimides **11**–**14**. The residue was analyzed by gas chromatography‐flame ionization detector (GC‐FID) and integration of the peak areas provided the proportion of the ester and phthalimides present in the mixture. The yields and the ratios of the products for each reaction can be found in Supporting Information Table S1 and Figs S1–S4.

#### 2.2.3 Procedure for the synthesis of epoxy tetrahydrophthalimides **15a**–**18a** and **15b**–**18b**

A mixture (0.50 g) containing the tetrahydrophthalimide **11**–**14** and their respective esters **7**–**10** was dissolved in DCM (10 mL) and *meta*‐chloroperbenzoic acid (*m*‐CPBA) was added in a 1:1.5 ratio (tetrahydrophthalimide/*m‐*CPBA), considering the estimated amount of tetrahydrophthalimide present in the mixture (Table S1). The reaction mixture was stirred for 1 h at room temperature, and then transferred to a separatory funnel, dissolved with DCM (30 mL) and washed with saturated aqueous solution of sodium bicarbonate (3 × 25 mL). The organic layer was dried over anhydrous magnesium sulfate, filtered and the solvent evaporated under reduced pressure. The residue was purified by silica gel column chromatography eluting with hexane/ethyl acetate (1:1) and subsequently recrystallized from diethyl ether, to provide the epoxy tetrahydrophthalimides **15a**–**18a** and **15b**–**18b**. Each reaction led to the isolation of two different diastereoisomers (e.g., **15a** and **15b**) as a mixture of enantiomers.

(1a*S*,2a*S*,5a*R*,6a*R*)‐ and (1a*R*,2a*R*,5a*S*,6a*S*)‐4‐(3‐Methoxyphenyl)‐1a‐methyltetrahydro‐1a*H*‐oxireno[2,3‐f]isoindole‐3,5(4*H*,5a*H*)‐dione (**15a**): Appearance: White crystalline solid. Thin‐layer chromatography (TLC): Rf = 0.5 hexane/ethyl acetate (1:1). Melting point (m.p.) 124.7–125.7 °C. Yield: 19%. IR (ATR, $\bar{\nu }$/cm^−1^): 3088, 2931, 1776, 1700, 1599, 1258, 1185, 790. ^1^H‐NMR (400 MHz, CDCl_3_, *δ* = 7.27 ppm): 1.40 (3H, s, H1), 2.01 (1H, dd, *J* = 15 and 10 Hz, H2y), 2.08 (1H, dd, *J* = 15 and 10 Hz, H6y), 2.50 (1H, dd, *J* = 15 and 8 Hz, H2x), 2.68 (1H, ddd, *J* = 15, 8 and 4 Hz, H6x), 2.98–3.20 (3H, m, H2a, H5a and H6a), 3.80 (3H, s, H7′), 6.79 (1H, t, *J* = 2 Hz, H2′), 6.84 (1H, ddd, *J* = 8, 2 and 1 Hz, H6′), 6.93 (1H, ddd, *J* = 8, 2 and 1 Hz, H4′), 7.36 (1H, t, *J* = 8 Hz, H5′). ^13^C‐NMR (100 MHz, CDCl_3_, *δ* = 77.0 ppm): 21.8 (C1), 23.8 (C6), 28.8 (C2), 35.3 (C2a), 36.9 (C5a), 55.0 (C1a), 55.4 (C7′), 56.1 (C6a), 112.2 (C2′), 114.5 (C4′), 118.6 (C6′), 129.9 (C5′), 132.7 (C1′), 160.1 (C3′), 178.5 (C3), 178.6 (C5). *m/z* (%): 287 ([M^⨥^], 90), 244 (40), 149 (44), 123 (32), 81 (38), 53 (41), 43 (100).

(1a*S*,2a*R*,5a*S*,6a*R*)‐ and (1a*R*,2a*S*,5a*R*,6a*S*)‐4‐(3‐Methoxyphenyl)‐1a‐methyltetrahydro‐1a*H*‐oxireno[2,3‐f]isoindole‐3,5(4*H*,5a*H*)‐dione (**15b**): Appearance: White crystalline solid. TLC: Rf = 0.2 hexane/ethyl acetate (1:1). M.p. 126.2–127.4 °C. Yield: 66%. IR (ATR, $\bar{\nu }$/cm^−1^): 3077, 2990, 2927, 1780, 1705, 1591, 1258, 1188, 849. ^1^H‐NMR (400 MHz, CDCl_3_, *δ* = 7.27 ppm): 1.32 (3H, s, H1), 2.18 (1H, dd, *J* = 15 and 7 Hz, H2y), 2.22 (1H, dd, *J* = 15 and 7 Hz, H6y), 2.59 (1H, d, *J* = 15 Hz, H2x), 2.77 (1H, dd, *J* = 15 and 4 Hz, H6x), 2.85–2.95 (2H, m, H2a and H5a), 3.04 (1H, d, *J* = 4 Hz, H6a), 3.80 (3H, s, H7′), 6.84 (1H, t, *J* = 2 Hz, H2′), 6.89 (1H, ddd, *J* = 8, 2 and 1 Hz, H6′), 6.92 (1H, ddd, *J* = 8, 2 and 1 Hz, H4′), 7.36 (1H, t, *J* = 8 Hz, H5′). ^13^C‐NMR (100 MHz, CDCl_3_, *δ* = 77.0 ppm): 21.9 (C1), 23.6 (C6), 28.0 (C2), 35.3 (C2a), 36.7 (C5a), 55.4 (C7′), 56.7 (C1a), 57.6 (C6a), 112.4 (C2′), 114.6 (C4′), 119.0 (C6′), 129.8 (C5′), 133.8 (C1′), 160.1 (C3′), 179.6 (C3), 179.7 (C5). *m/z* (%):287 ([M^⨥^], 100), 244 (22), 204 (50), 149 (50), 93 (56), 43 (78).

(1a*S*,2a*S*,5a*R*,6a*R*)‐ and (1a*R*,2a*R*,5a*S*,6a*S*)‐1a‐Methyl‐4‐(*p*‐tolyl)tetrahydro‐1a*H*‐oxireno[2,3‐f]isoindole‐3,5(4*H*,5a*H*)‐dione (**16a**): Appearance: White crystalline solid. TLC: Rf = 0.6 hexane/ethyl acetate (1:1). M.p. 149.5–150.5 °C. Yield: 28%. IR (ATR, $\bar{\nu }$/cm^−1^): 2935, 2845, 1769, 1696, 1510, 1186, 808. ^1^H‐NMR (400 MHz, CDCl_3_, *δ* = 7.27 ppm): 1.39 (3H, s, H1), 2.00 (1H, dd, *J* = 15 and 10 Hz, H2y), 2.07 (1H, dd, *J* = 15 and 10 Hz, H6y), 2.37 (3H, s, H7′), 2.50 (1H, dd, *J* = 15 and 8 Hz, H2x), 2.67 (1H, ddd, *J* = 15, 8 and 4 Hz, H6x), 2.98–3.19 (3H, m, H2a, H5a and H6a), 7.13 (2H, d, *J* = 8 Hz, H3′ and H5′), 7.27 (2H, d, *J* = 8 Hz, H2′ and H6′). ^13^C‐NMR (100 MHz, CDCl_3_, *δ* = 77.0 ppm): 21.1 (C1), 21.7 (C7′), 23.8 (C6), 28.7 (C2), 35.2 (C2a), 36.8 (C5a), 55.0 (C1a), 56.0 (C6a), 126.1 (C3′ and C5′), 129.0 (C4′), 129.8 (C2′ and C6′), 138.6 (C1′), 178.7 (C3), 178.8 (C5). *m/z* (%): 271 ([M^⨥^], 81), 228 (64), 133 (41), 81 (35), 53 (42), 43 (100).

(1a*S*,2a*R*,5a*S*,6a*R*)‐ and (1a*R*,2a*S*,5a*R*,6a*S*)‐1a‐Methyl‐4‐(*p*‐tolyl)tetrahydro‐1a*H*‐oxireno[2,3‐f]isoindole‐3,5(4*H*,5a*H*)‐dione (**16b**): Appearance: White crystalline solid. TLC: Rf = 0.3 hexane/ethyl acetate (1:1). M.p. 193.5–194.2 °C. Yield: 55%. IR (ATR, $\bar{\nu }$/cm^−1^): 2983, 2918, 2859, 1777, 1696, 1518, 1194, 821. ^1^H‐NMR (400 MHz, CDCl_3_, *δ* = 7.27 ppm): 1.32 (3H, s, H1), 2.16 (1H, dd, *J* = 15 and 7 Hz, H2y), 2.22 (1H, dd, *J* = 15 and 7 Hz, H6y), 2.37 (3H, s, H7′), 2.59 (1H, d, *J* = 15 Hz, H2x), 2.75 (1H, dd, *J* = 15 and 4 Hz, H6x), 2.85–2.95 (2H, m, H2a and H5a), 3.04 (1H, d, *J* = 4 Hz, H6a), 7.17 (2H, d, *J* = 8 Hz, H3′ and H5′), 7.25 (2H, d, *J* = 8 Hz, H2′ and H6′). ^13^C‐NMR (100 MHz, CDCl_3_, *δ* = 77.0 ppm): 21.2 (C1), 21.9 (C7′), 23.5 (C6), 27.9 (C2), 35.2 (C2a), 36.6 (C5a), 56.6 (C1a), 57.6 (C6a), 126.5 (C3′ and C5′), 129.7 (C2′ and C6′), 130.0 (C4′), 138.4 (C1′), 179.8 (C3), 179.9 (C5). *m/z* (%): 271 ([M^⨥^], 100), 228 (28), 188 (48), 133 (57), 93 (87), 43 (91).

(1a*S*,2a*S*,5a*R*,6a*R*)‐ and (1a*R*,2a*R*,5a*S*,6a*S*)‐4‐(4‐Fluorophenyl)‐1a‐methyltetrahydro‐1a*H*‐oxireno[2,3‐f]isoindole‐3,5(4*H*,5a*H*)‐dione (**17a**): Appearance: White crystalline solid. TLC: Rf = 0.7 hexane/ethyl acetate (1:1). M.p. 157.2–158.9 °C. Yield: 18%. IR (ATR, $\bar{\nu }$/cm^−1^): 3083, 2927, 1775, 1700, 1505, 1181, 843. ^1^H‐NMR (400 MHz, CDCl_3_, *δ* = 7.27 ppm): 1.40 (3H, s, H1), 2.00 (1H, dd, *J* = 15 and 10 Hz, H2y), 2.13 (1H, dd, *J* = 15 and 10 Hz, H6y), 2.52 (1H, dd, *J* = 15 and 8 Hz, H2x), 2.70 (1H, ddd, *J* = 15, 8 and 4 Hz, H6x), 3.05–3.19 (3H, m, H2a, H5a and H6a), 7.12–7.18 (2H, m, H3′ and H5′), 7.23–7.29 (2H, m, H2′ and H6′). ^13^C‐NMR (100 MHz, CDCl_3_, *δ* = 77.0 ppm): 21.7 (C1), 23.8 (C6), 28.7 (C2), 35.2 (C2a), 36.8 (C5a), 55.0 (C1a), 56.0 (C6a), 116.1 (C3′ and C5′, d, *J* = 23 Hz), 127.6 (C1′), 128.1 (C2′ and C6′, d, *J* = 9 Hz), 162.1 (C4′, d, *J* = 249 Hz), 178.5 (C3), 178.6 (C5). *m/z* (%): 275 ([M^⨥^], 31), 232 (54), 137 (19), 81 (28), 53 (30), 43 (100).

(1a*S*,2a*R*,5a*S*,6a*R*)‐ and (1a*R*,2a*S*,5a*R*,6a*S*)‐4‐(4‐Fluorophenyl)‐1a‐methyltetrahydro‐1a*H*‐oxireno[2,3‐f]isoindole‐3,5(4*H*,5a*H*)‐dione (**17b**): Appearance: White crystalline solid. TLC: Rf = 0.4 hexane/ethyl acetate (1:1). M.p. 153.4–154.1 °C. Yield: 73%. IR (ATR, $\bar{\nu }$/cm^−1^): 3069, 2928, 1777, 1698, 1508, 1193, 833. ^1^H‐NMR (400 MHz, CDCl_3_, *δ* = 7.27 ppm): 1.33 (3H, s, H1), 2.19 (1H, dd, *J* = 15 and 7 Hz, H2y), 2.23 (1H, dd, *J* = 15 and 7 Hz, H6y), 2.59 (1H, d, *J* = 15 Hz, H2x), 2.77 (1H, dd, *J* = 15 and 4 Hz, H6x), 2.86–2.96 (2H, m, H2a and H5a), 3.06 (1H, d, *J* = 4 Hz, H6a), 7.08–7.18 (2H, m, H3′ and H5′), 7.26–7.1831 (2H, m, H2′ and H6′). ^13^C‐NMR (100 MHz, CDCl_3_, *δ* = 77.0 ppm): 21.8 (C1), 23.5 (C6), 27.9 (C2), 35.3 (C2a), 36.6 (C5a), 56.7 (C1a), 57.6 (C6a), 116.0 (C3′ and C5′, d, *J* = 23 Hz), 128.0 (C2′ and C6′, d, *J* = 10 Hz), 128.6 (C1′), 162.2 (C4′, d, *J* = 248 Hz), 179.6 (C3), 179.7 (C5). *m/z* (%): 275 ([M^⨥^], 38), 232 (14), 192 (46), 93 (100), 81 (47), 43 (83).

(1a*S*,2a*S*,5a*R*,6a*R*)‐ and (1a*R*,2a*R*,5a*S*,6a*S*)‐4‐(3‐Fluorophenyl)‐1a‐methyltetrahydro‐1a*H*‐oxireno[2,3‐f]isoindole‐3,5(4*H*,5a*H*)‐dione (**18a**): Appearance: White crystalline solid. TLC: Rf = 0.6 hexane/ethyl acetate (1:1). M.p. 124.1–125.0 °C. Yield: 17%. IR (ATR, $\bar{\nu }$/cm^−1^): 2955, 1779, 1702, 1592, 1375, 1173, 821. ^1^H‐NMR (400 MHz, CDCl_3_, *δ* = 7.27 ppm): 1.41 (3H, s, H1), 2.02 (1H, dd, *J* = 15 and 10 Hz, H2y), 2.16 (1H, dd, *J* = 15 and 10 Hz, H6y), 2.52 (1H, dd, *J* = 15 and 8 Hz, H2x), 2.70 (1H, ddd, *J* = 15, 8 and 4 Hz, H6x), 2.98–3.25 (3H, m, H2a, H5a and H6a), 6.98–7.13 (3H, m, H2′, H4′ and H6′), 7.43–7.37 (1H, m, H5′). ^13^C‐NMR (100 MHz, CDCl_3_, *δ* = 77.0 ppm): 21.7 (C1), 23.8 (C6), 28.7 (C2), 35.3 (C2a), 36.8 (C5a), 55.0 (C1a), 56.0 (C6a), 113.9 (C4′, d, *J* = 24 Hz), 115.6 (C2′, d, *J* = 21 Hz), 121.9, (C6′, d, *J* = 3 Hz), 130.2 (C5′, d, *J* = 9 Hz), 133.0 (C1′, d, *J* = 10 Hz), 162.6 (C3′, d, *J* = 248 Hz), 178.2 (C3), 178.31 (C5). *m/z* (%): 275 [M^⨥^], (22), 232 (50), 81 (25), 55 (31), 43 (100).

(1a*S*,2a*R*,5a*S*,6a*R*)‐ and (1a*R*,2a*S*,5a*R*,6a*S*)‐4‐(3‐Fluorophenyl)‐1a‐methyltetrahydro‐1a*H*‐oxireno[2,3‐f]isoindole‐3,5(4*H*,5a*H*)‐dione (**18b**): Appearance: White crystalline solid. TLC: Rf = 0.4 hexane/ethyl acetate (1:1). M.p. 128.0–129.1 °C. Yield: 64%. IR (ATR, $\bar{\nu }$/cm^−1^): 3067, 2922, 1777, 1697, 1597, 1390, 1182, 849. ^1^H‐NMR (400 MHz, CDCl_3_, *δ* = 7.27 ppm): 1.34 (3H, s, H1), 2.20 (1H, dd, *J* = 15 and 7 Hz, H2y), 2.25 (1H, dd, *J* = 15 and 7 Hz, H6y), 2.61 (1H, d, *J* = 15 Hz, H2x), 2.79 (1H, dd, *J* = 15 and 4 Hz, H6x), 2.87–2.97 (2H, m, H2a and H5a), 3.07 (1H, d, *J* = 4 Hz, H6a), 7.04–7.17 (3H, m, H2′, H4′ and H6′), 7.35–7.51 (1H, m, H5′). ^13^C‐NMR (100 MHz, CDCl_3_, *δ* = 77.0 ppm): 21.8 (C1), 23.6 (C6), 28.0 (C2), 35.3 (C2a), 36.7 (C5a), 56.7 (C1a), 57.6 (C6a), 114.3 (C4′, d, *J* = 23 Hz), 115.5 (C2′, d, *J* = 23 Hz), 122.4 (C6′, d, *J* = 3 Hz), 130.2 (C5′, d, *J* = 9 Hz), 134.0 (C1′, d, *J* = 10 Hz), 162.6 (C3′, d, *J* = 247 Hz), 179.2 (C3), 179.3 (C5). *m/z* (%): 275 ([M^⨥^]), 275 (38), 232 (14), 192 (47), 93 (100), 81 (48), 43 (83).

### 2.3 Herbicidal bioassays

Bioassays were conducted using the epoxy tetrahydrophthalimides **15a–18a** and **15b–18b**, synthesized according to previously reported procedures.^34^ Lettuce (*L. sativa* ‘Vitória Santo Antão’), cucumber (*C. sativus* ‘Agristar do Brasil Ltda’), sorghum (*Sorghum bicolor*) and beggartick (*B. pilosa*) seeds were placed on germination paper in a Petri dish (5.5 cm diameter). The solution containing the dissolved compound (2.5 mL) was added to the Petri dish which was identified, sealed with plastic film, and transferred to the germination chamber (biological oxygen demand (BOD) incubator). The chamber was maintained at 25 °C in the absence of light for 120 h (5 days). The experiments were performed in a completely randomized design with three replications with 15 seeds of each species. The seeds of the plants were obtained commercially except sorghum and beggartick seeds which were collected at the campus of the Universidade Federal de Viçosa, Viçosa, MG, Brazil (altitude: 648.74 m; latitude: 20° 45′ 14″ S; longitude: 42° 52′ 53″ W).

The commercial herbicide Dual (Dual Gold Syngenta® Company, São Paulo, Brazil; *S*‐metolachlor) was used as positive control, using 0.3% aqueous dimethyl sulfoxide (DMSO) solution at the concentration of 500, 300, 150, 100, and 50 μm. Solutions of each tested substance were prepared in five distinct concentrations (500, 300, 150, 100, and 50 μm). The compounds were dissolved in DMSO and diluted with distilled water to prepare the most concentrated solution (500 μm) containing 0.3% DMSO. The remaining solutions were prepared from the 500 μm solution by the corresponding dilution with 0.3% aqueous DMSO.^31^, ^35^, ^36^

Statistical analyses were conducted using Microsoft Excel® software. The experiments were executed in triplicate. The data, expressed as percentage of shoots or roots growth inhibition with respect to untreated controls (water containing 0.3% DMSO), were analyzed using Tukey's test at 0.05 probability level.

After the period of 5 days, the aerial and root lengths were measured, and the values were expressed as percentage difference from the negative control. The plates were frozen at 0 °C for 24 h to facilitate handling of the seedlings. The lengths of roots and aerial parts of the seedlings were measured by digitalization of their photographic images and data were statistically analyzed.

The percentage of growth, *G* (%), of shoots and roots was calculated according to the following formula:

(1) $$G\;\left(\%\right)=\frac{\left(S-C\right)}{C}\times 100$$

where *S* is the average length of the germinating shoots or roots of the plants, and *C* corresponds to the average growth of the negative control. Aqueous solution containing 0.3% DMSO (*v*/*v*) was used as negative control.

### 2.4 Molecular docking

#### 2.4.1 Conformational search and ligand optimization

The three‐dimensional (3D) structures of the candidate molecules were prepared using the Spartan 14 v.1.1.4 software.^37^ Conformational searches were then performed, examining up to 1000 conformers through molecular dynamics simulations, using the MMFF (Merck Molecular Force Field). The most stable conformations, those with a population of 5% or greater according to the Boltzmann distribution, were saved in .*mol2* format using the same software.

#### 2.4.2 Selection of potential protein targets through ligand‐based virtual screening

The SwissSimilarity tool^38^, ^39^ (http://www.swisssimilarity.ch) was employed to search for ligands similar to the candidate molecules, with the goal of identifying potential biological targets. The parameters used in SwissSimilarity included the bioactive compounds class, the LigandExpo^40^ compound library, and a combined two‐dimensional (2D) and 3D screening method. Only proteins with ligand scores of 0.5 or higher were selected for the subsequent steps, which involved retrieving their structures from the RCSB Protein Data Bank (PDB)^41^ (https://www.rcsb.org/) and performing genome identification.

Using the Ugene v.50 program^42^ and the Clustal Omega 1.2.4^43^ alignment algorithm, the amino acid sequences were aligned, and their Hamming similarities were calculated, accounting for all gaps. When two or more amino acid sequences exhibited over 95% similarity, the enzyme with the best crystallographic resolution was selected. A search for similar proteins in plant genomes (taxid: 3193) was performed via the National Center for Biotechnology Information (NCBI, https://blast.ncbi.nlm.nih.gov/) using DELTA‐BLAST (Domain Enhanced Lookup Time Accelerated‐Basic Local Alignment Search Tool)^44^ with default parameters. Proteins with scores below 200 were discarded, while the remaining ones were used to search for similar proteins in the genomes of *Sorghum* spp. (taxid: 4557), *Cucumis* spp. (taxid: 3655), *Lactuca* (taxid: 4235), and *Bidens* spp. (taxid: 42336) (Table S12).

#### 2.4.3 Docking

The 3D structure of the protein (PDB ID: 5R92) and proteins with similarities of amino acid sequences in relation to the amino acid sequence of 5R92, were obtained from the RCSB PDB. Ligands, co‐crystallized residues, and water molecules were removed, and polar hydrogens were added using MGLTools 1.5.6.^45^ The protein and ligand were prepared, and a molecular docking study was conducted using AutoDock Vina^46^, ^47^ as computational software. The DogSiteScorer tool^48^, ^49^, ^50^ (https://proteins.plus/) was used to analyze the potential binding sites of the protein. The docking region (GridBox) with the highest score assigned by DogSiteScorer, which also contained the co‐crystallized ligand, had the following dimensions: 50.32 × 111.50 × 26.37 (*x*, *y*, *z*). The protein–ligand complex was analyzed for 2D interactions using LigPlot+^51^, ^52^ and for surface representation using ChimeraX.^53^, ^54^

## 3 RESULTS AND DISCUSSIONS

### 3.1 Synthesis

**Scheme 1:**
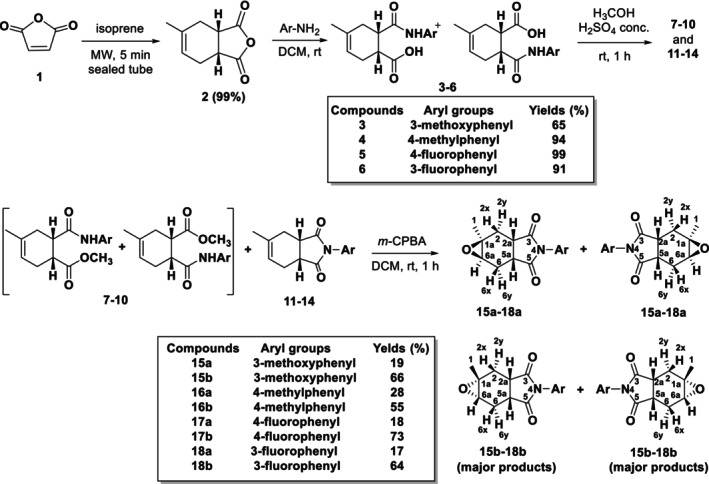
Synthesis of epoxy tetrahydrophthalimides **15a**–**18b**.

The synthetic route used to prepare eight epoxy tetrahydrophthalimides **15a**–**18a** and **15b**–**18b** is summarized in Scheme 1. In the first step, a microwave‐assisted Diels–Alder reaction between maleic anhydride **1** and isoprene produced the tetrahydrophthalic anhydride **2** in high yield. Subsequently, treatment of **2** was treated with aromatic amines to form mixtures of amides **3**–**6**. These mixtures were then treated with methanol in the presence of concentrated sulfuric acid, providing mixtures containing methyl esters **7**–**10** and tetrahydrophthalimides **11**–**14** (Table S1). Due to the difficulty in separating them, the mixtures obtained in the previous step were oxidized with *m*‐CPBA. The resulting diastereomers, **15a–18a** and **15b–18b** (major products), were separated by silica gel column chromatography, yielding racemic mixtures. Both enantiomers for **15a–18a** and **15b–18b** are represented in Scheme 1.

All the compounds obtained were characterized based on their spectrometric data. The spectra of the target compounds **15a**–**18a** and **15b**–**18b** are shown in Figs S5–S46. Due to the similarity of these compounds, we will describe the interpretation of the spectrometric data of compound **15a**.

In the IR spectrum of the epoxy tetrahydrophthalimide **15a** (Fig. S5), two bands due to C=O stretching were observed in 1776 and 1700 cm^−1^. The band at 1776 cm^−1^ corresponds to symmetric stretching of the imide carbonyls, while the band at 1700 cm^−1^ corresponds to asymmetric stretching of the imide carbonyls. Additionally, a strong band at 1185 cm^−1^ is assigned to the stretching of the C–N–C bonds.

In the ^1^H‐NMR spectrum of compound **15a** (Fig. S7 and Table S10) the signal with double doublet multiplicity at *δ* = 2.08 ppm with *J* = 15 and 10 Hz was assigned to hydrogen H6y, and the double doublet at *δ* = 2.01 ppm with *J* = 15 and 10 Hz was assigned to H2y. These coupling constants are due to geminal couplings H6x–H6y and H2x–H2y (*J* = 15 Hz) and vicinal couplings H6y–H5a and H2y–H2a (*J* = 10 Hz). The double doublet at *δ* = 2.50 ppm with *J* = 15 and 8 Hz was assigned to H2x, which is coupled with H2y (geminal) and H2a (vicinal). The double‐double doublet at *δ* = 2.68 ppm with *J* = 15, 8 and 4 Hz, was assigned to H6x, which is coupled to H6y/H5a/H6a. The multiplet at *δ* = 2.98–3.20 ppm, was assigned to H2a, H5a and H6a. The deshielded hydrogens of the *meta*‐substituted aromatic ring (H2′, H4′, H6′ and H5′) were observed at *δ* = 6.79–7.36 ppm. The signals of the imide carbonyls of compound **15a** (Fig. S10 and Table S11) are observed at *δ* = 178.5 and 178.6 ppm in the ^13^C‐NMR spectrum. The C1a and C6a carbons that make up the epoxide were marked, respectively, as peaks at *δ* = 55.0 ppm and 56.1 ppm. The methine carbons C2a and C5a were marked as *δ* = 35.3 ppm and 36.9 ppm, respectively. Methylenic carbons C2 and C6, shown as negative peaks in the ^13^C DEPT (distortionless enhancement by polarization transfer) spectrum (Fig. S11), were marked as *δ* = 28.8 and 23.8 ppm, respectively.

All assignments were made with the aid of 2D NMR experiments, such as COSY (correlation spectroscopy) contour map (Fig. S8), HMQC (heteronuclear multiple‐quantum correlation) (Fig. S12) and HMBC (heteronuclear multiple‐bond correlation) (Fig. S13). The relative stereochemistry of **15a** was confirmed by the cross‐correlations of H2a/H2x, H5a/H6x, and H6y/H6a observed in the NOESY (nuclear Overhauser effect spectroscopy) spectrum (Fig. S9).

### 3.2 Biological assay

The results of the bioassays were presented in bar graphs with their respective standard deviations. The values of inhibition and stimulation of the substances were evaluated according to the growth (positive values) or inhibition (negative values) of the root and stem of the tested seeds. Dual (Syngenta® Company, São Paulo, Brazil; *S*‐metolachlor) was used as positive control and an aqueous solution of 0.3% DMSO (*v*/*v*) was used as a negative control.

#### 3.2.1 Sorghum bicolor

**Figure 2:**
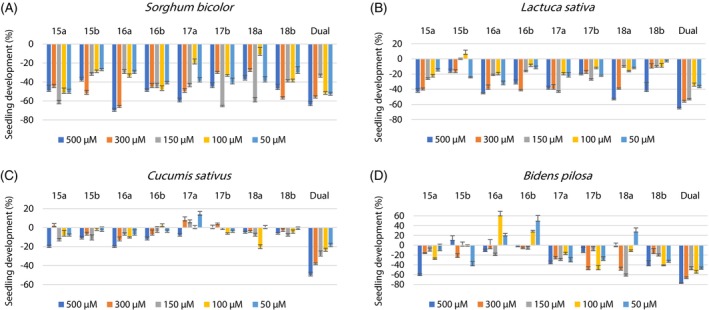
Development of the aerial parts of sorghum (A), lettuce (B), cucumber (C) and beggartick (D) plants in relation to the control. The error bars represent the standard deviation.

All substances showed inhibition activity on the aerial part of sorghum seeds. At concentrations of 500 and 300 μm substance **16a** presented inhibition of 70% and 66%, respectively, of the aerial parts of sorghum plants, which is higher than that observed for commercial herbicide. At 500 μm substance **17a** induced inhibition of 60% of the aerial parts. Substance **18b** at 300 μm presented inhibition of 57% of the stems, which is statistically comparable to the commercial herbicide at the same concentration (Fig. 2(A) and Table S2).

**Figure 3:**
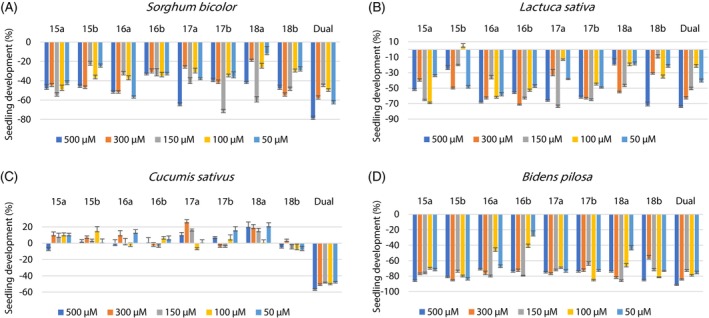
Development of the root parts of sorghum (A), lettuce (B), cucumber (C) and beggartick (D) plants in relation to the control. The error bars represent the standard deviation.

Assessing the development of the roots of sorghum seeds, we observed that all substances inhibited their growth. Substance **17a** at 500 μm presented inhibition percentage of the roots of 65% (Fig. 3(A) and Table S3).

#### 3.2.2 Lactuca sativa

All tested substances interfered in the development of the aerial parts of lettuce seeds in several different concentrations, some achieving similar inhibition results to that observed for commercial herbicide. At the concentration of 500 μm, substances **16a** and **18a** presented results close to 50% of growth inhibition of the aerial parts of lettuce seeds (Fig. 2(B) and Table S4).

Analyzing the graph of the root parts of lettuce seeds, it is observed that all the substances inhibited the development of the roots (Fig. 3(B) and Table S5).

#### 3.2.3 Cucumis sativus

The developments of the aerial parts of cucumber seeds were affected by all substances, however none of them displayed significant inhibition effects of these parts (Fig. 2(C) and Table S6).

All substances in most concentrations showed stimulus in the growth of cucumber roots, but the growth was not substantial (Fig. 3(C) and Table S7).

#### 3.2.4 Bidens pilosa

*Bidens pilosa* is a widely diffused agricultural weed in various countries. The seeds of this plant are attached to animals and human clothes and are easily dispersed by wind allowing it to promptly proliferate and infest new areas. This species quickly invades areas after fire destruction, as it is adapted to grow in a wide range of soil types.

All evaluated substances interfered in the development of the aerial parts of beggartick seeds, some of them inhibiting, others stimulating their growth. Substance **16a** at 100 μm was the only one that stimulated significantly seed growth (61%). At 500 μm substance **15a** restrained the development of the stems by 61% (Fig. 2(D) and Table S8).

The root parts of beggartick seeds were restrained by all substances in all concentrations evaluated. At the highest concentration, all substances prevented root growth by more than 70%, and **15a**, **15b**, and **18b** were the most active substances inhibiting by more than 80% this part of the plant (Fig. 3(D) and Table S9).

### 3.3 Molecular docking

The virtual screening performed by SwissSimilarity resulted in the initial selection of ligands complexed (Fig. S47) to the following proteins deposited in the PDB under the codes: 5IX0,^55^ 5QPV, 5SNB,^56^ 5QQV, 1XNN,^57^ 5IWG,^57^ 5AM4,^58^ 5J1Y,^59^ 4Y5E, 3UDK,^60^ 6CWY,^61^ 4Y58, 4DMX,^62^ 1C3S,^63^ 5RW3,^64^ 3ZV9,^65^ 5Q26, 3ZLW,^66^ 6ARK,^67^ 6X5B,^68^ 3UDR,^60^ 5RFQ,^69^ 5EZS,^70^ 7AXF,^71^ 5QEA,^72^ 2EXC, 7BJL,^73^ 3UDJ,^60^ 7O5P,^73^ 5R92,^74^ 4U3U,^75^ 4BDI,^75^ 4UXN,^76^ 5U2M,^77^ 2KO7,^77^ 1YKV,^78^ 5N17,^79^ 2IHQ,^79^ 5Q13,^80^ 5ZHP,^81^ 5CJ6,^82^ 3UDN,^60^ 5RE5,^69^ 2DC6, 6QEF,^83^ 4YM9,^84^ 3UDM,^60^ 6YJM,^85^ 5YQX,^86^ 2 DC9, 5KGT,^86^ 5NQE,^87^ 4GQI,^88^ 6QEG,^83^ 4X6H,^89^ 2W0A,^90^ 7B9L,^91^ 6XIH,^92^ 5DR1, 5NH0, 5RHE,^69^ 5RAV, 5PGU,^93^ 6V52,^94^ 3UDY^60^ and 5Q19.^80^

The ligands of these proteins exhibit similarity to the candidate compounds, suggesting that the compounds may also form stable complexes with these proteins. However, an analysis of plant genomes, using the amino acid sequences of these proteins, revealed that only eight proteins showed similarity to those found in the genomes of *Sorghum* spp. (taxid: 4557), *Cucumis* spp. (taxid: 3655), and *Lactuca* (taxid: 4235). No similar proteins were identified in the genome of *Bidens* spp. (taxid: 42336). Among the eight selected proteins, the best results were obtained for protein 5R92, which achieved scores above 370.

Protein 5R92, a member of the transferase class, is a mitogen‐activated protein kinase (MAPK) 14, also known as p38α. No 3D structure of p38α produced by *Sorghum bicolor*, *C. sativus*, *L. sativa*, or *B. pilosa* was found in the RCSB PDB. Therefore, the study proceeded with the 5R92 structure, which, as previously mentioned, has an amino acid sequence highly similar to those produced by several plants of the same genera as the species mentioned. In addition, 11 MAPK proteins (2FST, 5R8U, 5R8V, 5R9H, 5R9K, 5R9L, 5R9V, 5R93, 5R94, 5R97, and 2EXC) with amino acid sequences similar to 5R92 were also used to calculate their affinities for compounds **15a**, **15b**, **17a**, and **17b**. To establish comparative parameters, the binding affinity of ligand SO7 ((*S*)‐*N*‐methoxy‐1‐(4‐methoxyphenyl)‐*N*‐methyl‐5‐oxopyrrolidine‐3‐carboxamide) to the 5R92 protein was also determined. Additionally, the affinities of dihydroxylated derivatives of compounds **15a**/**15b** and **17a**/**17b** were evaluated in order to compare them with the other compounds studied.

MAPKs are critical enzymes in eukaryotic organisms, playing a fundamental role in the response to external stimuli and the regulation of cellular processes.^95^ They are integral components of signaling pathways involved in transducing signals related to abiotic and biotic stresses.^74^, ^96^, ^97^ Activation of MAPKs triggers a cascade of molecular events that regulate gene expression and physiological responses, promoting cellular adaptation and resistance to adverse conditions.^98^ In plants, MAPKs are associated with the regulation of growth, development, and nutrient uptake, as well as the modulation of immune responses. Consequently, studying MAPKs is crucial for understanding the molecular mechanisms that control stress resistance.

The 5R92 enzyme consists of 360 amino acid residues and contains four binding sites. One of these sites, known as the non‐canonical site, is used by the enzyme to form a complex with TAB1, which is involved in the activation of MAPKs. Any molecule capable of efficiently binding to this non‐canonical site could potentially block MAPK activation and, therefore, interfere with the activity of these enzymes. The compound called SO7 is complexed to the non‐canonical site of the 3D structure of MAPK 5R92, selected during the search for substances analogous to the compounds synthesized in this work.

**Figure 4:**
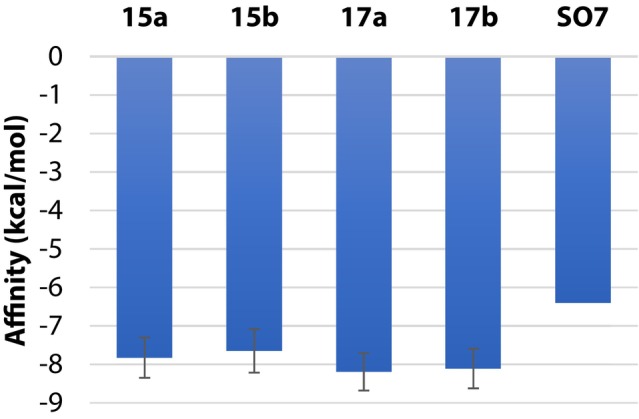
Affinities of compounds **15a**, **15b**, **17a**, and **17b** for the mitogen‐activated protein kinases 2FST, 5R8U, 5R8V, 5R9H, 5R9K, 5R9L, 5R9V, 5R93, 5R94, 5R97, and 2EXC, according to calculations carried out with the software AutoDock Vina. The binding affinity of ligand SO7 to the 5R92 protein was also computed for comparative analysis. Error bars are standard deviations.

Binding affinity calculations indicated that compounds **15a**, **15b**, **17a**, and **17b** (Fig. 4) exhibited favorable affinities, ranging from −7.65 to −8.19 kcal/mol. These values were higher than that observed for the reference ligand SO7, which showed a binding affinity of −6.40 kcal/mol, suggesting a greater potential for interaction of the evaluated compounds with the molecular target. The putative dihydroxylated derivatives of compounds **15a**/**15b** and **17a**/**17b** (Fig. S48) were employed to assess whether the hydrolysis of epoxides in acidic and basic aqueous media affects their binding affinity to the protein. The results indicated no significant differences in binding affinity between the epoxidized and the corresponding dihydroxylated compounds (Fig. S49).

The binding affinity and herbicidal efficacy of a compound are largely determined by the efficiency of hydrogen bonding and hydrophobic interactions with specific amino acid residues. These interactions play a pivotal role in the herbicidal activity of each compound, with the quality of hydrogen bonding serving as a critical parameter for elucidating their influence on target protein inhibition and the underlying mechanisms of action. The amino acid residues participating in these interactions are likely part of the herbicide action site, and the strength and efficiency of these bonds are key factors in determining the compounds' capacity to disrupt targeted biological pathways.

**Figure 5:**
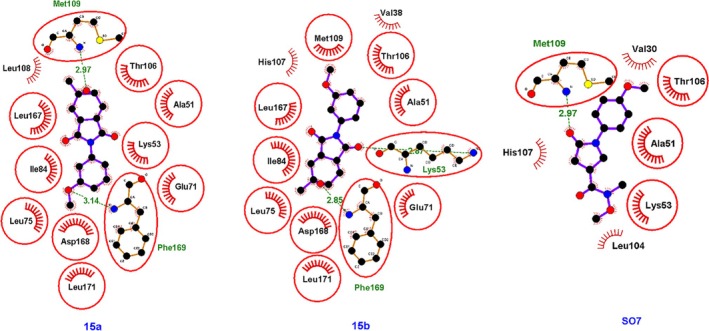
Two‐dimensional representation of the interactions of compounds **15a**, **15b** and SO7 with the protein 5R92.

The interactions of compounds **15a** and **15b** with the active site of the protein 5R92 are illustrated in Fig. 5. The analyzed compounds established hydrogen bonds with amino acid residues such as phenylalanine at Phe‐169. Compound **15a** also formed a hydrogen bond with the Met‐109 residue, which is the same residue involved in the interaction with the reference ligand SO7. The hydrophobic interactions observed for compound **15a** were also identified in compound **15b**. Additionally, the ligand SO7 exhibits hydrophobic interactions with residues Thr‐106, Ala‐51, and Lys‐53, which are also involved in interactions with compounds **15a** and **15b**, suggesting a similarity in their binding modes.

**Figure 6:**
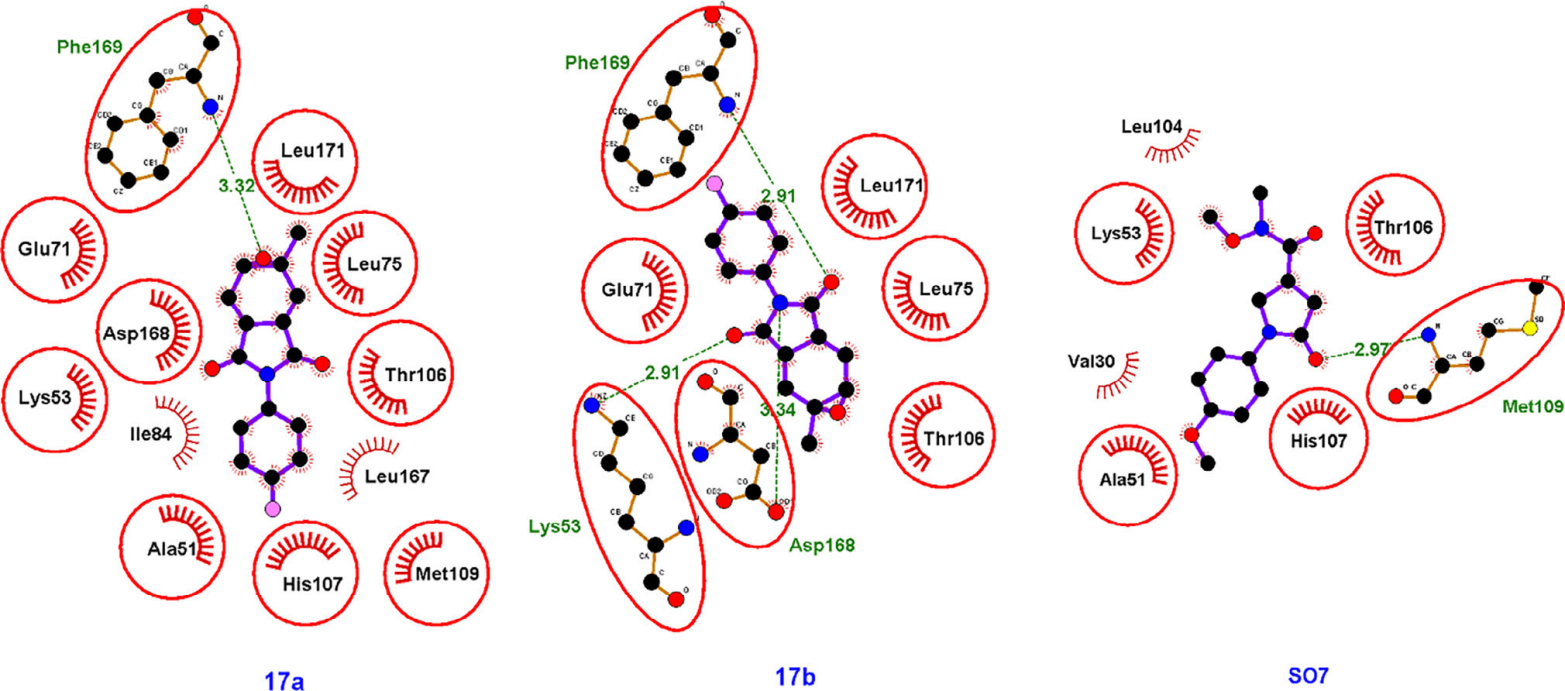
Two‐dimensional representation of the interactions of compounds **17a**, **17b** and SO7 with the protein 5R92.

**Figure 7:**
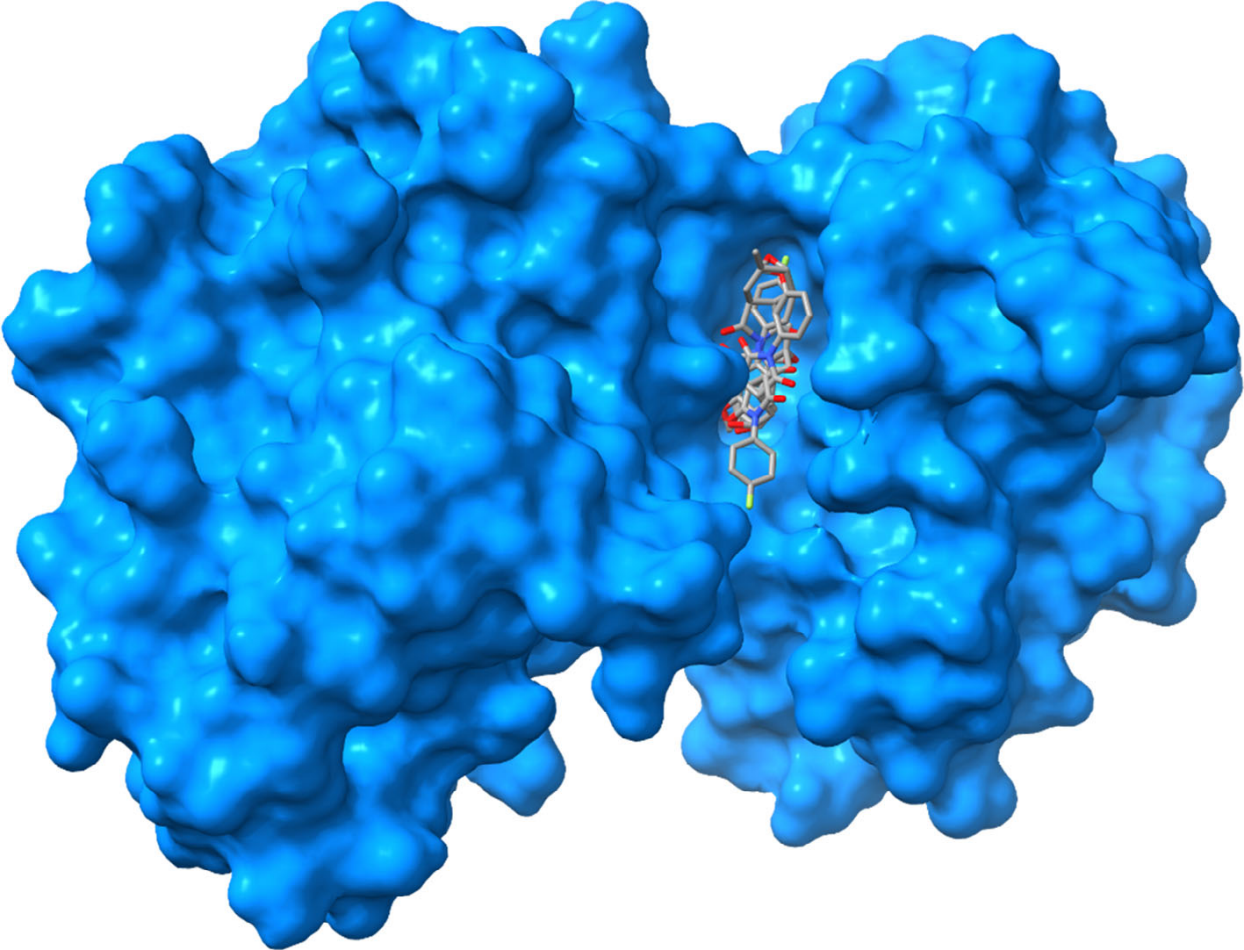
Representation of the surface of the 5R92 protein anchored to compounds **15a**, **15b**, **17a**, and **17b**.

Compounds **17a** and **17b** established hydrogen bonds with the amino acid residue Phe‐169 (Fig. 6). Additionally, compound **17b** formed hydrogen bonds with the residues Asp‐168 and Lys‐53. Hydrophobic interactions with the residues Thr‐106, Leu‐75, Leu‐171, and Glu‐71 were also observed for both compounds, indicating a consistent interaction profile between **17a** and **17b**. Figure 7 displays the surface representation of the protein 5R92 complexed with compounds **15a**, **15b**, **17a**, and **17b**. It is plausible to conclude that the effects observed for the tested compounds on the 5R92 protein may extend to other enzymes with similar amino acid sequences, representing a potential benefit for applications as pesticides. However, phthalimide‐based compounds should be handled with caution in laboratory settings, and any future use will depend on rigorous confirmation of their environmental and human safety. Extensive testing and regulatory approval will be essential steps before these compounds can be widely employed as herbicides.

## 4 CONCLUSIONS

Four pairs of diastereoisomers of epoxy tetrahydrophthalimides **15a** and **15b**; **16a** and **16b**; **17a** and **17b**; and **18a** and **18b** were synthesized from maleic anhydride **1** through four reaction steps.

The herbicidal potential of the target epoxides **15a**–**18a** and **15b**–**18b** was evaluated against *L. sativa*, *C. sativus*, *Sorghum bicolor*, and *B. pilosa*. All substances interfered with the growth of the evaluated seeds, with some showing better inhibition results than the commercial herbicide used as positive control, indicating a potential use of this class of compounds for the development of new agricultural protection agents. Significant inhibitions of root growth in lettuce, sorghum and beggartick were observed for all evaluated compounds.

The *in silico* study suggested that a possible enzymatic target for compounds is the MAPK, whose amino acid sequences have similarities with the enzymes produced by plants. The activity of the evaluated compounds can be associated with the electrophilic centers located at the epoxy ring and at the carbonyls of the imide. These electrophilic centers can bind to the amino acid residues from plant enzymes. The use of plant‐based bioactive substances also presents itself as a promising approach for the development and structural modification of molecules with herbicidal potential.

## CONFLICT OF INTEREST

The authors declare no conflict of interest.

## Supporting information


**Table S1. Reactions data for the step of formation of the tetrahydrophthalimides 11–14**.
**Figure S1.** Chromatogram of the mixture of tetrahydrophthalimide (11) and respective esters (7).
**Figure S2.** Chromatogram of the mixture of tetrahydrophthalimide (12) and respective esters (8).
**Figure S3.** Chromatogram of the mixture of tetrahydrophthalimide (13) and respective esters (9).
**Figure S4.** Chromatogram of the mixture of tetrahydrophthalimide (14) and respective esters (10).
**Figure S5.** IR spectrum of compound 15a.
**Figure S6.** Mass spectrum of the compound 15a.
**Figure S7.** 1H‐NMR spectrum (400 MHz, CDCl3 δCHCl3 = 7.27 ppm) of the compound 15a.
**Figure S8.** COSY contour map of compound 15a.
**Figure S9.** NOESY contour map of compound 15a.
**Figure S10.** 13C‐NMR spectrum (100 MHz, CDCl3 δCDCl3 = 77.0 ppm) of the compound 15a.
**Figure S11.** DEPT spectrum (100 MHz) of compound 15a.
**Figure S12.** HMQC contour map of compound 15a.
**Figure S13.** HMBC contour map of compound 15a.
**Figure S14.** IR spectrum of compound 15b.
**Figure S15.** Mass spectrum of the compound 15b.
**Figure S16.** 1H‐NMR spectrum (400 MHz, CDCl3 δCHCl3 = 7.27 ppm) of the compound 15b.
**Figure S17.** COSY contour map of compound 15b.
**Figure S18.** NOESY contour map of compound 15b.
**Figure S19.** 13C‐NMR spectrum (100 MHz, CDCl3 δCDCl3 = 77.0 ppm) of the compound 15b.
**Figure S20.** DEPT spectrum (100 MHz) of compound 15b.
**Figure S21.** HMQC contour map of compound 15b.
**Figure S22.** HMBC contour map of compound 15b.
**Figure S23.** IR spectrum of compound 16a.
**Figure S24.** Mass spectrum of the compound 16a.
**Figure S25.** 1H‐NMR spectrum (400 MHz, CDCl3 δCHCl3 = 7.27 ppm) of the compound 16a.
**Figure S26.** 13C‐NMR spectrum (100 MHz, CDCl3 δCDCl3 = 77.0 ppm) of the compound 16a.
**Figure S27.** IR spectrum of compound 16b.
**Figure S28.** Mass spectrum of the compound 16b.
**Figure S29.** 1H‐NMR spectrum (400 MHz, CDCl3 δCHCl3 = 7.27 ppm) of the compound 16b.
**Figure S30.** 13C‐NMR spectrum (100 MHz, CDCl3 δCDCl3 = 77.0 ppm) of the compound 16b.
**Figure S31.** IR spectrum of compound 17a.
**Figure S32.** Mass spectrum of the compound 17a.
**Figure S33.** 1H‐NMR spectrum (400 MHz, CDCl3 δCHCl3 = 7.27 ppm) of the compound 17a.
**Figure S34.** 13C‐NMR spectrum (100 MHz, CDCl3 δCDCl3 = 77.0 ppm) of the compound 17a.
**Figure S35.** IR spectrum of compound 17b.
**Figure S36.** Mass spectrum of the compound 17b.
**Figure S37.** 1H‐NMR spectrum (400 MHz, CDCl3 δCHCl3 = 7.27 ppm) of the compound 17b.
**Figure S38.** 13C‐NMR spectrum (100 MHz, CDCl3 δCDCl3 = 77.0 ppm) of the compound 17b.
**Figure S39.** IR spectrum of compound 18a.
**Figure S40.** Mass spectrum of the compound 18a.
**Figure S41.** 1H‐NMR spectrum (400 MHz, CDCl3 δCHCl3 = 7.27 ppm) of the compound 18a.
**Figure S42.** 13C‐NMR spectrum (100 MHz, CDCl3 δCDCl3 = 77.0 ppm) of the compound 18a.
**Figure S43.** IR spectrum of compound 18b.
**Figure S44.** Mass spectrum of the compound 18b.
**Figure S45.** 1H‐NMR spectrum (400 MHz, CDCl3 δCHCl3 = 7.27 ppm) of the compound 18b.
**Figure S46.** 13C‐NMR spectrum (100 MHz, CDCl3 δCDCl3 = 77.0 ppm) of the compound 18b.
**Table S2.** Development of aerial parts of sorghum plants in relation to the control.
**Table S3.** Development of root parts of sorghum plants in relation to the control.
**Table S4.** Development of the aerial parts of lettuce plants in relation to the control.
**Table S5.** Development of the root parts of lettuce plants in relation to the control.
**Table S6.** Development of the aerial parts of cucumber plants in relation to the control.
**Table S7.** Development of the root parts of cucumber plants in relation to the control.
**Table S8.** Development of aerial parts of beggartick plants in relation to the control.
**Table S9.** Development of root parts of beggartick plants in relation to the control.
**Figure S47.** Ligands complexed with proteins available in the PDB, identified based on the results obtained through SwissSimilarity. The bolded codes correspond to the identifiers deposited in the LigandExpo database.
**Figure S48.** Structures of the hydroxylated derivatives. Compound 15c corresponds to the hydroxylated derivative of 15a/15b obtained under acidic conditions, while compound 15d is the corresponding derivative formed in basic medium. Similarly, compound 17c is the hydroxylated derivative of 17a/17b in acidic conditions, and compound 17d is the derivative obtained under basic conditions.
**Figure S49.** Affinities of hydroxylated derivates 15c, 15d, 17c, and 17d for the mitogen‐activated protein kinases 2FST, 5R8U, 5R8V, 5R9H, 5R9K, 5R9L, 5R9V, 5R93, 5R94, 5R97, and 2EXC, according to calculations carried out with the software AutoDock Vina. Error bars are standard deviations.
**Table S10.** 1H‐NMR spectra data of products 15a–18a and 15b–18b (CDCl3, 400 MHz).
**Table S11.** 13C‐NMR spectra data of products 15a–18a and 15b–18b (CDCl3, 100 MHz).
**Table S12.** Amino acid sequences similar to that of the mitogen‐activated protein kinase 5R92 that were obtained by searching in the genome of plants through the National Center for Biotechnology Information (NCBI, http://www.ncbi.nlm.nih.gov), using BLAST, with DELTA‐BLAST (Domain Enhanced Lookup Time Accelerated BLAST) set to the default parameters.

## Data Availability

The data that supports the findings of this study are available in the supplementary material of this article.
