# Supplementary material for: Epoxy tetrahydrophthalimides as potential bioactive agents: synthesis and computational study
Source: Pest Manag Sci. 2025 Jul 30;81(12):7995–8006. doi: 10.1002/ps.70111 (PMC12618921; doi:10.1002/ps.70111)
Supplement: Supplementary file 1 — Table S1. Reactions data for the step of formation of the tetrahydrophthalimides 11–14. Figure S1. Chromatogram of the mixture of tetrahydrophthalimide (11) and respective esters (7). Figure S2. Chromatogram of the mixture of tetrahydrophthalimide (12) and respective esters (8). Figure S3. Chromatogram of the mixture of tetrahydrophthalimide (13) and respective esters (9). Figure S4. Chromatogram of the mixture of tetrahydrophthalimide (14) and respective esters (10). Figure S5. IR spectrum of compound 15a. Figure S6. Mass spectrum of the compound 15a. Figure S7. 1H‐NMR spectrum (400 MHz, CDCl3 δCHCl3 = 7.27 ppm) of the compound 15a. Figure S8. COSY contour map of compound 15a. Figure S9. NOESY contour map of compound 15a. Figure S10. 13C‐NMR spectrum (100 MHz, CDCl3 δCDCl3 = 77.0 ppm) of the compound 15a. Figure S11. DEPT spectrum (100 MHz) of compound 15a. Figure S12. HMQC contour map of compound 15a. Figure S13. HMBC contour map of compound 15a. Figure S14. IR spectrum of compound 15b. Figure S15. Mass spectrum of the compound 15b. Figure S16. 1H‐NMR spectrum (400 MHz, CDCl3 δCHCl3 = 7.27 ppm) of the compound 15b. Figure S17. COSY contour map of compound 15b. Figure S18. NOESY contour map of compound 15b. Figure S19. 13C‐NMR spectrum (100 MHz, CDCl3 δCDCl3 = 77.0 ppm) of the compound 15b. Figure S20. DEPT spectrum (100 MHz) of compound 15b. Figure S21. HMQC contour map of compound 15b. Figure S22. HMBC contour map of compound 15b. Figure S23. IR spectrum of compound 16a. Figure S24. Mass spectrum of the compound 16a. Figure S25. 1H‐NMR spectrum (400 MHz, CDCl3 δCHCl3 = 7.27 ppm) of the compound 16a. Figure S26. 13C‐NMR spectrum (100 MHz, CDCl3 δCDCl3 = 77.0 ppm) of the compound 16a. Figure S27. IR spectrum of compound 16b. Figure S28. Mass spectrum of the compound 16b. Figure S29. 1H‐NMR spectrum (400 MHz, CDCl3 δCHCl3 = 7.27 ppm) of the compound 16b. Figure S30. 13C‐NMR spectrum (100 MHz, CDCl3 δCDCl3 = 77.0 ppm) of the compound 16b. Figure S31. IR spectrum of c [file PS-81-7995-s001.docx]

**SUPPLEMENTARY INFORMATION**

**Epoxy Tetrahydrophthalimides as Potential Bioactive Agents: Synthesis and Computational Study**

Kariny Bragato Amorim Torrent, Vitor Cunha Baia, Laisa Samarini Gomes, Eloiza Ribeiro Castro, Vânia Maria Teixeira Carneiro, Elson Santiago Alvarenga*

Department of Chemistry, Universidade Federal de Viçosa, 36570-900, Viçosa, MG, Brazil

*****Corresponding author: elson@ufv.br

**Table S1:** Reactions data for the step of formation of the tetrahydrophthalimides **11**-**14**.

| **Starting materials** | **Mixture of products**  **(esters + imide)** | **Yields of the mixtures**  **(esters + imide)^a^** | **Mixtures ratio**  **(imide: esters)^b^** | **Yields of the imides^c^** |
| --- | --- | --- | --- | --- |
| **3** | **7** + **11** | 95% | 63:37 | 60% |
| **4** | **8** + **12** | 98% | 74:26 | 73% |
| **5** | **9** + **13** | 99% | 69:31 | 68% |
| **6** | **10** + **14** | 99% | 62:38 | 61% |

^a^Isolated yields. ^b^The proportions were calculated based on the peak areas obtained from the chromatograms of the reaction mixtures. ^c^Yields were calculated from peak areas.

**Figure S1:** Chromatogram of the mixture of tetrahydrophthalimide **(11)** and respective esters **(7)**.

**Figure S2:** Chromatogram of the mixture of tetrahydrophthalimide **(12)** and respective esters **(8)**.

**Figure S3:** Chromatogram of the mixture of tetrahydrophthalimide **(13)** and respective esters **(9)**.

**Figure S4:** Chromatogram of the mixture of tetrahydrophthalimide **(14)** and respective esters **(10)**.

**Figure S5**: IR spectrum of compound **15a**.

**Figure S6:** Mass spectrum of the compound **15a**.

**Figure S7:** ^1^H NMR spectrum (400 MHz, CDCl_3_ δ_CHCl3_ = 7.27 ppm) of the compound **15a**.

**Figure S8:** COSY contour map of compound **15a**.

**Figure S9:** NOESY contour map of compound **15a**.

**Figure S10:** ^13^C NMR spectrum (100 MHz, CDCl_3_ δ_CDCl3_ = 77.0 ppm) of the compound **15a**.

**Figure S11:** DEPT spectrum (100 MHz) of compound **15a**.

**Figure S12:** HMQC contour map of compound **15a**.

**Figure S13:** HMBC contour map of compound **15a**.

**Figure S14**: IR spectrum of compound **15b**.

**Figure S15:** Mass spectrum of the compound **15b**.

**Figure S16:** ^1^H NMR spectrum (400 MHz, CDCl_3_ δ_CHCl3_ = 7.27 ppm) of the compound **15b**.

**Figure S17:** COSY contour map of compound **15b**.

**Figure S18:** NOESY contour map of compound **15b**.

**Figure S19:** ^13^C NMR spectrum (100 MHz, CDCl_3_ δ_CDCl3_ = 77.0 ppm) of the compound **15b**.

**Figure S20:** DEPT spectrum (100 MHz) of compound **15b**.

**Figure S21:** HMQC contour map of compound **15b**.

**Figure S22:** HMBC contour map of compound **15b**.

**Figure S23**: IR spectrum of compound **16a**.

**Figure S24:** Mass spectrum of the compound **16a**.

**Figure S25:** ^1^H-NMR spectrum (400 MHz, CDCl_3_ δ_CHCl3_ = 7.27 ppm) of the compound **16a**.


**Figure S26:** ^13^C NMR spectrum (100 MHz, CDCl_3_ δ_CDCl3_ = 77.0 ppm) of the compound **16a**.

**Figure S27**: IR spectrum of compound **16b**.

**Figure S28:** Mass spectrum of the compound **16b**.

**Figure S29:** ^1^H NMR spectrum (400 MHz, CDCl_3_ δ_CHCl3_ = 7.27 ppm) of the compound **16b**.

**Figure S30:** ^13^C NMR spectrum (100 MHz, CDCl_3_ δ_CDCl3_ = 77.0 ppm) of the compound **16b**.

**Figure S31**: IR spectrum of compound **17a**.

**Figure S32:** Mass spectrum of the compound **17a**.

**Figure S33:** ^1^H-NMR spectrum (400 MHz, CDCl_3_ δ_CHCl3_ = 7.27 ppm) of the compound **17a**.

**Figure S34:** ^13^C NMR spectrum (100 MHz, CDCl_3_ δ_CDCl3_ = 77.0 ppm) of the compound **17a**.

**Figure S34**: IR spectrum of compound **17b**.

**Figure S36:** Mass spectrum of the compound **17b**.

**Figure S37:** ^1^H-NMR spectrum (400 MHz, CDCl_3_ δ_CHCl3_ = 7.27 ppm) of the compound **17b**.

**Figure S38:** ^13^C NMR spectrum (100 MHz, CDCl_3_ δ_CDCl3_ = 77.0 ppm) of the compound **17b**.

**Figure S39**: IR spectrum of compound **18a**.

**Figure S40:** Mass spectrum of the compound **18a**.

**Figure S41:** ^1^H-NMR spectrum (400 MHz, CDCl_3_ δ_CHCl3_ = 7.27 ppm) of the compound **18a**.

**Figure S42:** ^13^C NMR spectrum (100 MHz, CDCl_3_ δ_CDCl3_ = 77.0 ppm) of the compound **18a**.

**Figure S43**: IR spectrum of compound **18b**.

**Figure S44:** Mass spectrum of the compound **18b**.

**Figure S45:** ^1^H-NMR spectrum (400 MHz, CDCl_3_ δ_CHCl3_ = 7.27 ppm) of the compound **18b**.

**Figure S46:** ^13^C NMR spectrum (100 MHz, CDCl_3_ δ_CDCl3_ = 77.0 ppm) of the compound **18b**.

**Table S2:** Development of aerial parts of sorghum plants in relation to the control.

|  | Shoot length (% of control) | | | | |
| --- | --- | --- | --- | --- | --- |
| **Compounds** | **500** *µ*M | **300** *µ*M | **150** *µ*M | **100** *µ*M | **50** *µ*M |
| **15a** | -49c | -45d | -62a | -51a | -51a |
| **15b** | -38d | -52c | -32d | -29d | -27d |
| **16a** | -70a | -66a | -30d | -34d | -30d |
| **16b** | -49c | -45d | -45b | -48b | -41b |
| **17a** | -60b | -50c | -44b | -20f | -39c |
| **17b** | -45c | -30e | -65a | -33d | -41b |
| **18a** | -37d | -28e | -60b | -11e | -39c |
| **18b** | -47c | -57b | -39c | -39c | -30d |
| **Dual** | -64b | -56b | -34d | -52a | -53a |

Data are means of three replicates. Water containing 0.3% DMSO (v/v) treatment was used as contrast. Mean values in the same column with the same letter are not significantly different at P= 0.05% by Tukey’s test.

**Table S3:** Development of root parts of sorghum plants in relation to the control.

|  | Root length (% of control) | | | | |
| --- | --- | --- | --- | --- | --- |
| **Compounds** | **500** *µ*M | **300** *µ*M | **150** *µ*M | **100** *µ*M | **50** *µ*M |
| **15a** | -48d | -45bc | -55c | -49a | -43c |
| **15b** | -46d | -47b | -23g | -37b | -25e |
| **16a** | -52c | -52a | -33f | -38b | -57b |
| **16b** | -33f | -31d | -34f | -35b | -33de |
| **17a** | -65b | -26e | -42e | -31c | -38d |
| **17b** | -40e | -42c | -72a | -35c | -36d |
| **18a** | -42de | -19f | -61b | -26d | -12g |
| **18b** | -48d | -55a | -49d | -30c | -29e |
| **Dual** | -79a | -58a | -45de | -50a | -63a |

Data are means of three replicates. Water containing 0.3% DMSO (v/v) treatment was used as contrast. Mean values in the same column with the same letter are not significantly different at P= 0.05% by Tukey’s test.

**Table S4:** Development of the aerial parts of lettuce plants in relation to the control.

|  | Shoot length (% of control) | | | | |
| --- | --- | --- | --- | --- | --- |
| **Compounds** | **500** *µ*M | **300** *µ*M | **150** *µ*M | **100** *µ*M | **50** *µ*M |
| **15a** | -43c | -40b | -26c | -23b | -15c |
| **15b** | -17f | -17c | 0g | 7d | -24b |
| **16a** | -45c | -39b | -21d | -20b | -33a |
| **16b** | -32e | -41b | -16e | -9c | -12c |
| **17a** | -39d | -39b | -43b | -20b | -23b |
| **17b** | -20f | -18c | -27c | -12c | -22b |
| **18a** | -53b | -39b | -10f | -16bc | -12c |
| **18b** | -42c | -11d | -10f | -10c | -3d |
| **Dual** | -65a | -56a | -53a | -35a | -37a |

Data are means of three replicates. Water containing 0.3% DMSO (v/v) treatment was used as contrast. Mean values in the same column with the same letter are not significantly different at P= 0.05% by Tukey’s test.

**Table S5:** Development of the root parts of lettuce plants in relation to the control.

|  | Root length (% of control) | | | | |
| --- | --- | --- | --- | --- | --- |
| **Compounds** | **500** *µ*M | **300** *µ*M | **150** *µ*M | **100** *µ*M | **50** *µ*M |
| **15a** | -52c | -40f | -65b | -69a | -34d |
| **15b** | -25d | -50d | -20e | 4h | -49b |
| **16a** | -68b | -63b | -37d | -62b | -59a |
| **16b** | -56c | -71a | -63b | -53c | -48b |
| **17a** | -66b | -33e | -74a | -13g | -38d |
| **17b** | -62b | -63b | -65b | -45d | -49b |
| **18a** | -19e | -55c | -47c | -20f | -19e |
| **18b** | -72a | -31g | -10f | -36e | -22e |
| **Dual** | -74a | -63b | -51c | -22f | -41c |

Data are means of three replicates. Water containing 0.3% DMSO (v/v) treatment was used as contrast. Mean values in the same column with the same letter are not significantly different at P= 0.05% by Tukey’s test.

**Table S6:** Development of the aerial parts of cucumber plants in relation to the control.

|  | Shoot length (% of control) | | | | |
| --- | --- | --- | --- | --- | --- |
| **Compounds** | **500** *µ*M | **300** *µ*M | **150** *µ*M | **100** *µ*M | **50** *µ*M |
| **15a** | -20b | 2d | -13b | -8bc | -8b |
| **15b** | -11c | -7e | -12bc | -2d | -3cd |
| **16a** | -20b | -13b | -7d | -10b | -7bc |
| **16b** | -12cd | -7e | -4de | 2d | -4cd |
| **17a** | -8de | 8c | 6f | 0d | 14b |
| **17b** | 0 | 4cd | -1e | -6cd | -4cd |
| **18a** | -5e | -4e | -8c | -21a | 0e |
| **18b** | -6e | -3e | -8c | -5cd | -1d |
| **Dual** | -50a | -38a | -29a | -24a | -19a |

Data are means of three replicates. Water containing 0.3% DMSO (v/v) treatment was used as contrast. Mean values in the same column with the same letter are not significantly different at P= 0.05% by Tukey’s test.

**Table S7:** Development of the root parts of cucumber plants in relation to the control.

|  | Root length (% of control) | | | | |
| --- | --- | --- | --- | --- | --- |
| **Compounds** | **500** *µ*M | **300** *µ*M | **150** *µ*M | **100** *µ*M | **50** *µ*M |
| **15a** | -8b | 10d | 8e | 10e | 10d |
| **15b** | 2d | 7cd | 3d | 15f | 1c |
| **16a** | -2c | 10d | -1c | -3b | 13de |
| **16b** | 1d | -3b | -4bc | 6de | 5c |
| **17a** | 10e | 26f | 16b | -7b | 1c |
| **17b** | 7e | -4b | -4bc | 5cd | 16e |
| **18a** | 20f | 19e | 15f | 1c | 21f |
| **18b** | -5bc | 3c | -6b | -7b | -8b |
| **Dual** | -57a | -51a | -48a | -50a | -48a |

Data are means of three replicates. Water containing 0.3% DMSO (v/v) treatment was used as contrast. Mean values in the same column with the same letter are not significantly different at P= 0.05% by Tukey’s test.

**Table S8:** Development of aerial parts of beggartick plants in relation to the control.

|  | Shoot length (% of control) | | | | |
| --- | --- | --- | --- | --- | --- |
| **Compounds** | **500** *µ*M | **300** *µ*M | **150** *µ*M | **100** *µ*M | **50** *µ*M |
| **15a** | -61b | -16d | -11e | -28e | -10d |
| **15b** | 11f | -24c | -1g | -1g | -41b |
| **16a** | -12d | -6e | -20d | 61a | 20e |
| **16b** | -2e | -6e | -7f | 28b | 50g |
| **17a** | -37c | -27c | -30c | -18 | -33c |
| **17b** | -14d | -49b | -10e | -49c | -30c |
| **18a** | -2e | -50b | -62a | -12f | 28f |
| **18b** | -41c | -16d | -21d | -41d | -34c |
| **Dual** | -77a | -67a | -49b | -56 | -48a |

Data are means of three replicates. Water containing 0.3% DMSO (v/v) treatment was used as contrast. Mean values in the same column with the same letter are not significantly different at P= 0.05% by Tukey’s test.

**Table S9:** Development of root parts of beggartick plants in relation to the control.

|  | Root length (% of control) | | | | |
| --- | --- | --- | --- | --- | --- |
| **Compounds** | **500** *µ*M | **300** *µ*M | **150** *µ*M | **100** *µ*M | **50** *µ*M |
| **15a** | -86b | -77b | -76bc | -70d | -72b |
| **15b** | -81c | -85a | -74c | -80c | -84a |
| **16a** | -71d | -77b | -80b | -47e | -68c |
| **16b** | -74d | -73b | -79b | -42e | -27e |
| **17a** | -75d | -77b | -72c | -69d | -74b |
| **17b** | -74d | -73b | -65d | -85ab | -73b |
| **18a** | -74d | -82a | -86a | -67d | -45d |
| **18b** | -85bc | -57c | -72c | -81bc | -73b |
| **Dual** | -91a | -84a | -73c | -79c | -76b |

Data are means of three replicates. Water containing 0.3% DMSO (v/v) treatment was used as contrast. Mean values in the same column with the same letter are not significantly different at P= 0.05% by Tukey’s test.

**Figure S47:** Ligands complexed with proteins available in the PDB, identified based on the results obtained through SwissSimilarity. The bolded codes correspond to the identifiers deposited in the LigandExpo database.

**Figure S48**: Structures of the hydroxylated derivatives. Compound **15c** corresponds to the hydroxylated derivative of **15a**/**15b** obtained under acidic conditions, while compound **15d** is the corresponding derivative formed in basic medium. Similarly, compound **17c** is the hydroxylated derivative of **17a**/**17b** in acidic conditions, and compound **17d** is the derivative obtained under basic conditions.

**Figure S49:** Affinities of hydroxylated derivates **15c**, **15d**, **17c**, and **17d** for the mitogen-activated protein kinases 2FST, 5R8U, 5R8V, 5R9H, 5R9K, 5R9L, 5R9V, 5R93, 5R94, 5R97, and 2EXC, according to calculations carried out with the software AutoDock Vina. Error bars are standard deviations.

**Table S10:** ^1^H NMR spectra data of products **15a-18a** and **15b-18b** (CDCl_3_, 400 MHz).

| H | *δ*_H_ (ppm), m, *J* (Hz) | | | | | | | |
| --- | --- | --- | --- | --- | --- | --- | --- | --- |
|  | **15a** | **15b** | **16a** | **16b** | **17a** | **17b** | **18a** | **18b** |
| H1 | 1.40 (s) | 1.32 (s) | 1.39 (s) | 1.32 (s) | 1.40 (s) | 1.33 (s) | 1.41 (s) | 1.34 (s) |
| H2y | 2.01 (dd, 15, 10) | 2.18 (dd, 15, 7) | 2.00 (dd, 15, 10) | 2.16 (dd, 15, 7) | 2.00 (dd, 15, 10) | 2.19 (dd, 15, 7) | 2.02 (dd, 15, 10) | 2.20 (dd, 15, 7) |
| H6y | 2.08 (dd, 15, 10) | 2.22 (dd, 15, 7) | 2.07 (dd, 15, 10) | 2.22 (dd, 15, 7) | 2.13 (dd, 15, 10) | 2.23 (dd, 15, 7) | 2.16 (dd, 15, 10) | 2.25 (1H, dd, *J* = 15, 7) |
| H2x | 2.50 (dd, 15, 8) | 2.59 (d, 15) | 2.50 (dd, 15, 8) | 2.59 (d, 15) | 2.52 (dd, 15, 8) | 2.59 (d, 15) | 2.52 (dd, 15, 8) | 2.61 (d, 15) |
| H6x | 2.68 (ddd, 15, 8, 4) | 2.77 (dd, 15, 4) | 2.67 (ddd, 15, 8, 4) | 2.75 (dd, 15, 4) | 2.70 (ddd, 15, 8, 4) | 2.77 (dd, 15, 4) | 2.70 (ddd, 15, 8, 4) | 2.79 (dd, 15, 4) |
| H2a | 2.98-3.20 (m) | 2.85-2,95 (m) | 2.98-3.19 (m) | 2.85-2,95 (m) | 3.05-3.19 (m) | 2.86-2,96 (m) | 2.98-3.25 (m) | 2.87-2.97 (m) |
| H5a | 2.98-3.20 (m) | 2.85-2,95 (m) | 2.98-3.19 (m) | 2.85-2,95 (m) | 3.05-3.19 (m) | 2.86-2,96 (m) | 2.98-3.25 (m) | 2.87-2.97 (m) |
| H6a | 2.98-3.20 (m) | 3.04 (d, 4) | 2.98-3.19 (m) | 3.04 (d, 4) | 3.05-3.19 (m) | 3.06 (d, 4) | 2.98-3.25 (m) | 3.07 (d, 4) |
| H7’ | 3.80 (s) | 3.80 (s) | 2.37 (s) | 2.37 (s) |  |  |  |  |
| H2’ | 6.79 (t, 2) | 6.84 (t, 2) | 7.27 (d, 8) | 7.25 (d, 8) | 7.23-7.29 (m) | 7.26-7.18(m) | 6.98-7.13 (m) | 7.04-7.17 (m) |
| H6’ | 6.84 (ddd, 8, 2, 1) | 6.89 (ddd, 8, 2, 1) | 7.27 (d, 8) | 7.25 (d, 8) | 7.23-7.29 (m) | 7.26-7.18 (m) | 6.98-7.13 (m) | 7.04-7.17 (m) |
| H4’ | 6.93 (ddd, 8, 2 1) | 6.92 (ddd, 8, 2 1 Hz) |  |  |  |  | 6.98-7.13 (m) | 7.04-7.17 (m) |
| H5’ | 7.36 (t, 8) | 7.36 (t, 8) | 7.13 (d, 8) | 7.17 (d, 8) | 7.12-7.18 (m) | 7.08-7.18 (m) | 7.43-7.37 (m) | 7.35-7.51 (m) |
| H3’ |  |  | 7.13 (d, 8) | 7.17 (d, 8) | 7.12-7.18 (m) | 7.08-7.18 (m) |  |  |

**Table S11:** ^13^C NMR spectra data of products **15a-18a** and **15b-18b** (CDCl_3_, 100 MHz).

| C | *δ*_H_ (ppm), m, *J* (Hz) | | | | | | | |
| --- | --- | --- | --- | --- | --- | --- | --- | --- |
|  | **15a** | **15b** | **16a** | **16b** | **17a** | **17b** | **18a** | **18b** |
| C1 | 21.8 | 21.9 | 21.1 | 21.2 | 21.7 | 21.8 | 21.7 | 21.8 |
| C6 | 23.8 | 23.6 | 23.8 | 23.5 | 23.8 | 23.5 | 23.8 | 23.6 |
| C2 | 28.8 | 28.0 | 28.7 | 27.9 | 28.7 | 27.9 | 28.7 | 28.0 |
| C2a | 35.3 | 35.3 | 35.2 | 35.2 | 35.2 | 35.3 | 35.3 | 35.3 |
| C5a | 36.9 | 36.7 | 36.8 | 36.6 | 36.8 | 36.6 | 36.8 | 36.7 |
| C1a | 55.0 | 56.7 | 55.0 | 56.6 | 55.0 | 56.7 | 55.0 | 56.7 |
| C7’ | 55.4 | 54.5 | 21.7 | 21.9 |  |  |  |  |
| C6a | 56.1 | 57.6 | 56.0 | 57.6 | 56.0 | 57.6 | 56.0 | 57.6 |
| C2’ | 112.2 | 112.4 | 129.8 | 129.7 | 128.1 (d, 9) | 128.0 (d, 10) | 115.6 (d, 21) | 115.5 (d, 23) |
| C4’ | 114.5 | 114.6 | 129.0 | 130.0 | 162.1 (d, 249) | 162.2 (d, 248) | 113.9 (d, 24) | 114.3 (d, 23) |
| C6’ | 118.6 | 119.0 | 129.8 | 129.7 | 128.1 (d, 9) | 128.0 (d, 10) | 121.9 (d, 3) | 122.4 (d, 3) |
| C5’ | 129.9 | 129.8 | 126.1 | 126.5 | 116.1 (d, 23) | 116.0 (d, 23) | 130.2 (d, 9) | 130.2 (d, 9) |
| C1’ | 132.7 | 133.8 | 138.6 | 138.4 | 127.6 | 128.6 | 133.0 (d, 10) | 134.0 (d, 10) |
| C3’ | 160.1 | 160.1 | 126.1 | 126.5 | 116.1 (d, 23) | 116.0 (d, 23) | 162.6 (d, 248) | 162.6 (d, 247) |
| C3 | 178.5 | 179.6 | 178.7 | 179.8 | 178.5 | 179.6 | 178.2 | 179.2 |
| C5 | 178.6 | 179.7 | 178.8 | 179.9 | 178.6 | 179.7 | 178.31 | 179.3 |

**Table S12:** Amino acid sequences similar to that of the mitogen-activated protein kinase 5R92 that were obtained by searching in the genome of plants through the National Center for Biotechnology Information-NCBI (http://www.ncbi.nlm.nih.gov), using BLAST, with DELTA-BLAST (Domain Enhanced Lookup Time Accelerated BLAST) set to the default parameters.

| **Protein [plant species]** | **Max Score** | **Total Score** | **Query Cover** | **Accession** |
| --- | --- | --- | --- | --- |
| mitogen-activated protein kinase 6 [Sorghum bicolor] | 381 | 381 | 97% | =HYPERLINK("https://www.ncbi.nlm.nih.gov/protein/XP_002467591.1?report=genbank&log$=prottop&blast_rank=1&RID=EK4FW9P8013","XP_002467591.1") |
| mitogen-activated protein kinase homolog MMK2 [Cucumis sativus] | 376 | 376 | 96% | =HYPERLINK("https://www.ncbi.nlm.nih.gov/protein/XP_004138470.1?report=genbank&log$=prottop&blast_rank=2&RID=EK4FW9P8013","XP_004138470.1") |
| mitogen-activated protein kinase-like protein MMK2 [Cucumis melo var. makuwa] | 376 | 376 | 96% | =HYPERLINK("https://www.ncbi.nlm.nih.gov/protein/TYK03037.1?report=genbank&log$=prottop&blast_rank=3&RID=EK4FW9P8013","TYK03037.1") |
| mitogen-activated protein kinase 4-2 [Cucumis sativus] | 375 | 375 | 96% | =HYPERLINK("https://www.ncbi.nlm.nih.gov/protein/AKP99748.1?report=genbank&log$=prottop&blast_rank=4&RID=EK4FW9P8013","AKP99748.1") |
| mitogen-activated protein kinase homolog MMK2 [Cucumis melo] | 374 | 374 | 96% | =HYPERLINK("https://www.ncbi.nlm.nih.gov/protein/XP_008458174.2?report=genbank&log$=prottop&blast_rank=5&RID=EK4FW9P8013","XP_008458174.2") |
| mitogen-activated protein kinase homolog MMK2 [Lactuca sativa] | 371 | 371 | 95% | =HYPERLINK("https://www.ncbi.nlm.nih.gov/protein/XP_023758764.1?report=genbank&log$=prottop&blast_rank=6&RID=EK4FW9P8013","XP_023758764.1") |
| unnamed protein product [Lactuca virosa] | 371 | 371 | 95% | =HYPERLINK("https://www.ncbi.nlm.nih.gov/protein/CAH1412025.1?report=genbank&log$=prottop&blast_rank=7&RID=EK4FW9P8013","CAH1412025.1") |
| unnamed protein product [Lactuca saligna] | 373 | 373 | 95% | =HYPERLINK("https://www.ncbi.nlm.nih.gov/protein/CAI9259235.1?report=genbank&log$=prottop&blast_rank=8&RID=EK4FW9P8013","CAI9259235.1") |
| mitogen-activated protein kinase homolog NTF6 [Lactuca sativa] | 370 | 370 | 95% | =HYPERLINK("https://www.ncbi.nlm.nih.gov/protein/XP_023738592.1?report=genbank&log$=prottop&blast_rank=9&RID=EK4FW9P8013","XP_023738592.1") |
| unnamed protein product [Lactuca saligna] | 377 | 377 | 95% | =HYPERLINK("https://www.ncbi.nlm.nih.gov/protein/CAI9277977.1?report=genbank&log$=prottop&blast_rank=10&RID=EK4FW9P8013","CAI9277977.1") |
| mitogen-activated protein kinase 4-like [Cucumis melo] | 370 | 370 | 95% | =HYPERLINK("https://www.ncbi.nlm.nih.gov/protein/XP_008437327.1?report=genbank&log$=prottop&blast_rank=11&RID=EK4FW9P8013","XP_008437327.1") |
| unnamed protein product [Lactuca saligna] | 368 | 368 | 95% | =HYPERLINK("https://www.ncbi.nlm.nih.gov/protein/CAI9297882.1?report=genbank&log$=prottop&blast_rank=12&RID=EK4FW9P8013","CAI9297882.1") |
| mitogen-activated protein kinase 3 [Cucumis melo] | 368 | 368 | 95% | =HYPERLINK("https://www.ncbi.nlm.nih.gov/protein/NP_001284376.1?report=genbank&log$=prottop&blast_rank=13&RID=EK4FW9P8013","NP_001284376.1") |
| unnamed protein product [Lactuca virosa] | 367 | 367 | 95% | =HYPERLINK("https://www.ncbi.nlm.nih.gov/protein/CAH1446878.1?report=genbank&log$=prottop&blast_rank=14&RID=EK4FW9P8013","CAH1446878.1") |
| unnamed protein product [Lactuca saligna] | 367 | 367 | 95% | =HYPERLINK("https://www.ncbi.nlm.nih.gov/protein/CAI9301111.1?report=genbank&log$=prottop&blast_rank=15&RID=EK4FW9P8013","CAI9301111.1") |
| hypothetical protein IC582_027823 [Cucumis melo] | 366 | 366 | 95% | =HYPERLINK("https://www.ncbi.nlm.nih.gov/protein/KAL0533778.1?report=genbank&log$=prottop&blast_rank=16&RID=EK4FW9P8013","KAL0533778.1") |
| mitogen-activated protein kinase 4 [Sorghum bicolor] | 365 | 365 | 99% | =HYPERLINK("https://www.ncbi.nlm.nih.gov/protein/XP_021304350.1?report=genbank&log$=prottop&blast_rank=17&RID=EK4FW9P8013","XP_021304350.1") |
| mitogen-activated protein kinase 3-like [Cucumis sativus] | 365 | 365 | 95% | =HYPERLINK("https://www.ncbi.nlm.nih.gov/protein/NP_001267653.1?report=genbank&log$=prottop&blast_rank=18&RID=EK4FW9P8013","NP_001267653.1") |
| mitogen-activated protein kinase homolog NTF6 isoform X1 [Lactuca sativa] | 363 | 363 | 95% | =HYPERLINK("https://www.ncbi.nlm.nih.gov/protein/XP_023734730.1?report=genbank&log$=prottop&blast_rank=19&RID=EK4FW9P8013","XP_023734730.1") |
| hypothetical protein BDA96_01G436600 [Sorghum bicolor] | 363 | 363 | 95% | =HYPERLINK("https://www.ncbi.nlm.nih.gov/protein/KAG0551594.1?report=genbank&log$=prottop&blast_rank=20&RID=EK4FW9P8013","KAG0551594.1") |
| mitogen-activated protein kinase homolog NTF3 [Cucumis sativus] | 362 | 362 | 96% | =HYPERLINK("https://www.ncbi.nlm.nih.gov/protein/XP_004152682.1?report=genbank&log$=prottop&blast_rank=21&RID=EK4FW9P8013","XP_004152682.1") |
| mitogen-activated protein kinase homolog NTF3 [Cucumis melo] | 362 | 362 | 96% | =HYPERLINK("https://www.ncbi.nlm.nih.gov/protein/XP_008444747.1?report=genbank&log$=prottop&blast_rank=22&RID=EK4FW9P8013","XP_008444747.1") |
| mitogen-activated protein kinase 5 [Sorghum bicolor] | 362 | 362 | 95% | =HYPERLINK("https://www.ncbi.nlm.nih.gov/protein/XP_021305804.1?report=genbank&log$=prottop&blast_rank=23&RID=EK4FW9P8013","XP_021305804.1") |
| unnamed protein product [Lactuca saligna] | 361 | 361 | 95% | =HYPERLINK("https://www.ncbi.nlm.nih.gov/protein/CAI9263303.1?report=genbank&log$=prottop&blast_rank=24&RID=EK4FW9P8013","CAI9263303.1") |
| mitogen-activated protein kinase homolog NTF3 [Lactuca sativa] | 361 | 361 | 97% | =HYPERLINK("https://www.ncbi.nlm.nih.gov/protein/XP_023745437.1?report=genbank&log$=prottop&blast_rank=25&RID=EK4FW9P8013","XP_023745437.1") |
| unnamed protein product [Lactuca saligna] | 361 | 361 | 97% | =HYPERLINK("https://www.ncbi.nlm.nih.gov/protein/CAI9297872.1?report=genbank&log$=prottop&blast_rank=26&RID=EK4FW9P8013","CAI9297872.1") |
| mitogen-activated protein kinase 3 [Lactuca sativa] | 360 | 360 | 97% | =HYPERLINK("https://www.ncbi.nlm.nih.gov/protein/XP_023748812.1?report=genbank&log$=prottop&blast_rank=27&RID=EK4FW9P8013","XP_023748812.1") |
| unnamed protein product [Lactuca virosa] | 361 | 361 | 95% | =HYPERLINK("https://www.ncbi.nlm.nih.gov/protein/CAH1441271.1?report=genbank&log$=prottop&blast_rank=28&RID=EK4FW9P8013","CAH1441271.1") |
| unnamed protein product [Lactuca virosa] | 360 | 360 | 97% | =HYPERLINK("https://www.ncbi.nlm.nih.gov/protein/CAH1432370.1?report=genbank&log$=prottop&blast_rank=29&RID=EK4FW9P8013","CAH1432370.1") |
| mitogen-activated protein kinase homolog NTF4 [Lactuca sativa] | 361 | 361 | 95% | =HYPERLINK("https://www.ncbi.nlm.nih.gov/protein/XP_023737925.1?report=genbank&log$=prottop&blast_rank=30&RID=EK4FW9P8013","XP_023737925.1") |
| mitogen-activated protein kinase 2 [Sorghum bicolor] | 360 | 360 | 96% | =HYPERLINK("https://www.ncbi.nlm.nih.gov/protein/XP_002443888.1?report=genbank&log$=prottop&blast_rank=31&RID=EK4FW9P8013","XP_002443888.1") |
| mitogen-activated protein kinase homolog MMK1 [Cucumis sativus] | 360 | 360 | 95% | =HYPERLINK("https://www.ncbi.nlm.nih.gov/protein/XP_004144187.1?report=genbank&log$=prottop&blast_rank=32&RID=EK4FW9P8013","XP_004144187.1") |
| mitogen-activated protein kinase-like protein MMK1 isoform X1 [Cucumis melo var. makuwa] | 360 | 360 | 95% | =HYPERLINK("https://www.ncbi.nlm.nih.gov/protein/KAA0064742.1?report=genbank&log$=prottop&blast_rank=33&RID=EK4FW9P8013","KAA0064742.1") |
| mitogen-activated protein kinase 6 [Cucumis sativus] | 360 | 360 | 95% | =HYPERLINK("https://www.ncbi.nlm.nih.gov/protein/AKP99754.1?report=genbank&log$=prottop&blast_rank=34&RID=EK4FW9P8013","AKP99754.1") |
| hypothetical protein IC582_005980 [Cucumis melo] | 359 | 359 | 95% | =HYPERLINK("https://www.ncbi.nlm.nih.gov/protein/KAL0557442.1?report=genbank&log$=prottop&blast_rank=35&RID=EK4FW9P8013","KAL0557442.1") |
| unnamed protein product [Lactuca saligna] | 360 | 360 | 97% | =HYPERLINK("https://www.ncbi.nlm.nih.gov/protein/CAI9281659.1?report=genbank&log$=prottop&blast_rank=36&RID=EK4FW9P8013","CAI9281659.1") |
| mitogen-activated protein kinase 1 [Sorghum bicolor] | 359 | 359 | 95% | =HYPERLINK("https://www.ncbi.nlm.nih.gov/protein/XP_002437855.1?report=genbank&log$=prottop&blast_rank=37&RID=EK4FW9P8013","XP_002437855.1") |
| unnamed protein product [Lactuca virosa] | 357 | 357 | 95% | =HYPERLINK("https://www.ncbi.nlm.nih.gov/protein/CAH1446888.1?report=genbank&log$=prottop&blast_rank=38&RID=EK4FW9P8013","CAH1446888.1") |
| unnamed protein product [Lactuca virosa] | 354 | 354 | 88% | =HYPERLINK("https://www.ncbi.nlm.nih.gov/protein/CAH1432379.1?report=genbank&log$=prottop&blast_rank=39&RID=EK4FW9P8013","CAH1432379.1") |
| mitogen-activated protein kinase 3 [Cucumis melo var. makuwa] | 355 | 355 | 95% | =HYPERLINK("https://www.ncbi.nlm.nih.gov/protein/KAA0037795.1?report=genbank&log$=prottop&blast_rank=40&RID=EK4FW9P8013","KAA0037795.1") |
| mitogen-activated protein kinase 3 [Lactuca sativa] | 353 | 353 | 95% | =HYPERLINK("https://www.ncbi.nlm.nih.gov/protein/XP_023734727.1?report=genbank&log$=prottop&blast_rank=41&RID=EK4FW9P8013","XP_023734727.1") |
| mitogen-activated protein kinase 3 [Sorghum bicolor] | 353 | 353 | 97% | =HYPERLINK("https://www.ncbi.nlm.nih.gov/protein/XP_002453297.1?report=genbank&log$=prottop&blast_rank=42&RID=EK4FW9P8013","XP_002453297.1") |
| mitogen-activated protein kinase 3 [Cucumis melo var. makuwa] | 353 | 353 | 95% | =HYPERLINK("https://www.ncbi.nlm.nih.gov/protein/TYK02886.1?report=genbank&log$=prottop&blast_rank=43&RID=EK4FW9P8013","TYK02886.1") |
| mitogen-activated protein kinase 7 [Cucumis melo] | 352 | 352 | 96% | =HYPERLINK("https://www.ncbi.nlm.nih.gov/protein/XP_008452104.1?report=genbank&log$=prottop&blast_rank=44&RID=EK4FW9P8013","XP_008452104.1") |
| mitogen-activated protein kinase 15-like isoform X1 [Sorghum bicolor] | 358 | 358 | 93% | =HYPERLINK("https://www.ncbi.nlm.nih.gov/protein/XP_021316899.1?report=genbank&log$=prottop&blast_rank=45&RID=EK4FW9P8013","XP_021316899.1") |
| mitogen-activated protein kinase homolog NTF3 [Lactuca sativa] | 352 | 352 | 96% | =HYPERLINK("https://www.ncbi.nlm.nih.gov/protein/XP_023733127.1?report=genbank&log$=prottop&blast_rank=46&RID=EK4FW9P8013","XP_023733127.1") |
| mitogen-activated protein kinase 7 [Cucumis sativus] | 351 | 351 | 95% | =HYPERLINK("https://www.ncbi.nlm.nih.gov/protein/XP_011653154.1?report=genbank&log$=prottop&blast_rank=47&RID=EK4FW9P8013","XP_011653154.1") |
| mitogen-activated protein kinase 15 isoform X1 [Cucumis melo] | 358 | 358 | 98% | =HYPERLINK("https://www.ncbi.nlm.nih.gov/protein/XP_008458544.1?report=genbank&log$=prottop&blast_rank=48&RID=EK4FW9P8013","XP_008458544.1") |
| mitogen-activated protein kinase 15 isoform X2 [Cucumis melo] | 357 | 357 | 98% | =HYPERLINK("https://www.ncbi.nlm.nih.gov/protein/XP_008458545.1?report=genbank&log$=prottop&blast_rank=49&RID=EK4FW9P8013","XP_008458545.1") |
| unnamed protein product [Lactuca virosa] | 350 | 350 | 97% | =HYPERLINK("https://www.ncbi.nlm.nih.gov/protein/CAH1442849.1?report=genbank&log$=prottop&blast_rank=50&RID=EK4FW9P8013","CAH1442849.1") |
| mitogen-activated protein kinase 15 isoform X1 [Cucumis melo var. makuwa] | 357 | 357 | 94% | =HYPERLINK("https://www.ncbi.nlm.nih.gov/protein/KAA0033433.1?report=genbank&log$=prottop&blast_rank=51&RID=EK4FW9P8013","KAA0033433.1") |
| mitogen-activated protein kinase 15 isoform X1 [Cucumis sativus] | 356 | 356 | 98% | =HYPERLINK("https://www.ncbi.nlm.nih.gov/protein/XP_011656640.1?report=genbank&log$=prottop&blast_rank=52&RID=EK4FW9P8013","XP_011656640.1") |
| mitogen-activated protein kinase homolog NTF3 isoform X2 [Lactuca sativa] | 349 | 349 | 97% | =HYPERLINK("https://www.ncbi.nlm.nih.gov/protein/XP_023767183.1?report=genbank&log$=prottop&blast_rank=53&RID=EK4FW9P8013","XP_023767183.1") |
| mitogen-activated protein kinase 15 isoform X2 [Cucumis sativus] | 356 | 356 | 98% | =HYPERLINK("https://www.ncbi.nlm.nih.gov/protein/XP_004153181.1?report=genbank&log$=prottop&blast_rank=54&RID=EK4FW9P8013","XP_004153181.1") |
| mitogen-activated protein kinase homolog NTF3 isoform X1 [Lactuca sativa] | 349 | 349 | 97% | =HYPERLINK("https://www.ncbi.nlm.nih.gov/protein/XP_023767182.1?report=genbank&log$=prottop&blast_rank=55&RID=EK4FW9P8013","XP_023767182.1") |
| hypothetical protein LSAT_V11C100030370 [Lactuca sativa] | 350 | 350 | 95% | =HYPERLINK("https://www.ncbi.nlm.nih.gov/protein/KAJ0226007.1?report=genbank&log$=prottop&blast_rank=56&RID=EK4FW9P8013","KAJ0226007.1") |
| mitogen-activated protein kinase 14-like [Sorghum bicolor] | 355 | 355 | 94% | =HYPERLINK("https://www.ncbi.nlm.nih.gov/protein/XP_021303315.1?report=genbank&log$=prottop&blast_rank=57&RID=EK4FW9P8013","XP_021303315.1") |
| mitogen-activated protein kinase 7 [Lactuca sativa] | 348 | 348 | 96% | =HYPERLINK("https://www.ncbi.nlm.nih.gov/protein/XP_023741081.1?report=genbank&log$=prottop&blast_rank=58&RID=EK4FW9P8013","XP_023741081.1") |
| unnamed protein product [Lactuca virosa] | 350 | 350 | 96% | =HYPERLINK("https://www.ncbi.nlm.nih.gov/protein/CAH1413601.1?report=genbank&log$=prottop&blast_rank=59&RID=EK4FW9P8013","CAH1413601.1") |
| unnamed protein product [Lactuca virosa] | 354 | 354 | 94% | =HYPERLINK("https://www.ncbi.nlm.nih.gov/protein/CAH1449592.1?report=genbank&log$=prottop&blast_rank=60&RID=EK4FW9P8013","CAH1449592.1") |
| mitogen-activated protein kinase 15 isoform X3 [Lactuca sativa] | 354 | 354 | 94% | =HYPERLINK("https://www.ncbi.nlm.nih.gov/protein/XP_023763680.1?report=genbank&log$=prottop&blast_rank=61&RID=EK4FW9P8013","XP_023763680.1") |
| mitogen-activated protein kinase 15 isoform X4 [Lactuca sativa] | 354 | 354 | 94% | =HYPERLINK("https://www.ncbi.nlm.nih.gov/protein/XP_023763686.1?report=genbank&log$=prottop&blast_rank=62&RID=EK4FW9P8013","XP_023763686.1") |
| mitogen-activated protein kinase 15 isoform X1 [Lactuca sativa] | 353 | 353 | 94% | =HYPERLINK("https://www.ncbi.nlm.nih.gov/protein/XP_042752287.1?report=genbank&log$=prottop&blast_rank=63&RID=EK4FW9P8013","XP_042752287.1") |
| mitogen-activated protein kinase 15 isoform X2 [Lactuca sativa] | 353 | 353 | 94% | =HYPERLINK("https://www.ncbi.nlm.nih.gov/protein/XP_042752288.1?report=genbank&log$=prottop&blast_rank=64&RID=EK4FW9P8013","XP_042752288.1") |
| unnamed protein product [Lactuca saligna] | 347 | 347 | 96% | =HYPERLINK("https://www.ncbi.nlm.nih.gov/protein/CAI9276512.1?report=genbank&log$=prottop&blast_rank=65&RID=EK4FW9P8013","CAI9276512.1") |
| mitogen-activated protein kinase 9 isoform X4 [Cucumis melo] | 349 | 349 | 93% | =HYPERLINK("https://www.ncbi.nlm.nih.gov/protein/XP_008463378.1?report=genbank&log$=prottop&blast_rank=66&RID=EK4FW9P8013","XP_008463378.1") |
| mitogen-activated protein kinase 9 isoform X2 [Cucumis melo] | 349 | 349 | 93% | =HYPERLINK("https://www.ncbi.nlm.nih.gov/protein/XP_008463177.1?report=genbank&log$=prottop&blast_rank=67&RID=EK4FW9P8013","XP_008463177.1") |
| mitogen-activated protein kinase 9 isoform X3 [Cucumis melo] | 349 | 349 | 93% | =HYPERLINK("https://www.ncbi.nlm.nih.gov/protein/XP_008463306.1?report=genbank&log$=prottop&blast_rank=68&RID=EK4FW9P8013","XP_008463306.1") |
| mitogen-activated protein kinase 7 isoform X1 [Sorghum bicolor] | 352 | 352 | 93% | =HYPERLINK("https://www.ncbi.nlm.nih.gov/protein/XP_002441528.1?report=genbank&log$=prottop&blast_rank=69&RID=EK4FW9P8013","XP_002441528.1") |
| hypothetical protein Csa_013967 [Cucumis sativus] | 348 | 348 | 93% | =HYPERLINK("https://www.ncbi.nlm.nih.gov/protein/KGN64165.1?report=genbank&log$=prottop&blast_rank=70&RID=EK4FW9P8013","KGN64165.1") |
| mitogen-activated protein kinase 7 isoform X2 [Sorghum bicolor] | 351 | 351 | 93% | =HYPERLINK("https://www.ncbi.nlm.nih.gov/protein/XP_021303027.1?report=genbank&log$=prottop&blast_rank=71&RID=EK4FW9P8013","XP_021303027.1") |
| mitogen-activated protein kinase 9 isoform X1 [Cucumis melo] | 349 | 349 | 93% | =HYPERLINK("https://www.ncbi.nlm.nih.gov/protein/XP_050936165.1?report=genbank&log$=prottop&blast_rank=72&RID=EK4FW9P8013","XP_050936165.1") |
| mitogen-activated protein kinase 9 isoform X3 [Cucumis sativus] | 348 | 348 | 93% | =HYPERLINK("https://www.ncbi.nlm.nih.gov/protein/XP_031743880.1?report=genbank&log$=prottop&blast_rank=73&RID=EK4FW9P8013","XP_031743880.1") |
| mitogen-activated protein kinase 16 [Sorghum bicolor] | 351 | 351 | 98% | =HYPERLINK("https://www.ncbi.nlm.nih.gov/protein/XP_021313210.1?report=genbank&log$=prottop&blast_rank=74&RID=EK4FW9P8013","XP_021313210.1") |
| mitogen-activated protein kinase 9 isoform X2 [Cucumis sativus] | 348 | 348 | 93% | =HYPERLINK("https://www.ncbi.nlm.nih.gov/protein/XP_004137496.2?report=genbank&log$=prottop&blast_rank=75&RID=EK4FW9P8013","XP_004137496.2") |
| mitogen-activated protein kinase 9 isoform X1 [Cucumis sativus] | 348 | 348 | 93% | =HYPERLINK("https://www.ncbi.nlm.nih.gov/protein/XP_011650742.1?report=genbank&log$=prottop&blast_rank=76&RID=EK4FW9P8013","XP_011650742.1") |
| unnamed protein product [Lactuca saligna] | 352 | 352 | 94% | =HYPERLINK("https://www.ncbi.nlm.nih.gov/protein/CAI9293943.1?report=genbank&log$=prottop&blast_rank=77&RID=EK4FW9P8013","CAI9293943.1") |
| Pkinase domain-containing protein [Cucumis melo var. makuwa] | 342 | 342 | 86% | =HYPERLINK("https://www.ncbi.nlm.nih.gov/protein/KAA0042729.1?report=genbank&log$=prottop&blast_rank=78&RID=EK4FW9P8013","KAA0042729.1") |
| unnamed protein product [Lactuca saligna] | 352 | 352 | 94% | =HYPERLINK("https://www.ncbi.nlm.nih.gov/protein/CAI9293944.1?report=genbank&log$=prottop&blast_rank=79&RID=EK4FW9P8013","CAI9293944.1") |
| hypothetical protein BDA96_03G255500 [Sorghum bicolor] | 347 | 347 | 98% | =HYPERLINK("https://www.ncbi.nlm.nih.gov/protein/KAG0538660.1?report=genbank&log$=prottop&blast_rank=80&RID=EK4FW9P8013","KAG0538660.1") |
| hypothetical protein LSAT_V11C800419500 [Lactuca sativa] | 341 | 341 | 86% | =HYPERLINK("https://www.ncbi.nlm.nih.gov/protein/KAJ0191860.1?report=genbank&log$=prottop&blast_rank=81&RID=EK4FW9P8013","KAJ0191860.1") |
| hypothetical protein SORBI_3003G248100 [Sorghum bicolor] | 348 | 348 | 93% | =HYPERLINK("https://www.ncbi.nlm.nih.gov/protein/KXG33058.1?report=genbank&log$=prottop&blast_rank=82&RID=EK4FW9P8013","KXG33058.1") |
| hypothetical protein BDA96_03G268500 [Sorghum bicolor] | 348 | 348 | 93% | =HYPERLINK("https://www.ncbi.nlm.nih.gov/protein/KAG0538806.1?report=genbank&log$=prottop&blast_rank=83&RID=EK4FW9P8013","KAG0538806.1") |
| mitogen-activated protein kinase 8 isoform X3 [Sorghum bicolor] | 348 | 348 | 93% | =HYPERLINK("https://www.ncbi.nlm.nih.gov/protein/XP_021312072.1?report=genbank&log$=prottop&blast_rank=84&RID=EK4FW9P8013","XP_021312072.1") |
| hypothetical protein BDA96_03G268500 [Sorghum bicolor] | 348 | 348 | 93% | =HYPERLINK("https://www.ncbi.nlm.nih.gov/protein/KAG0538804.1?report=genbank&log$=prottop&blast_rank=85&RID=EK4FW9P8013","KAG0538804.1") |
| hypothetical protein BDA96_03G268500 [Sorghum bicolor] | 348 | 348 | 93% | =HYPERLINK("https://www.ncbi.nlm.nih.gov/protein/KAG0538805.1?report=genbank&log$=prottop&blast_rank=86&RID=EK4FW9P8013","KAG0538805.1") |
| unnamed protein product [Lactuca virosa] | 338 | 338 | 83% | =HYPERLINK("https://www.ncbi.nlm.nih.gov/protein/CAH1420065.1?report=genbank&log$=prottop&blast_rank=87&RID=EK4FW9P8013","CAH1420065.1") |
| mitogen-activated protein kinase 8 isoform X2 [Sorghum bicolor] | 348 | 348 | 93% | =HYPERLINK("https://www.ncbi.nlm.nih.gov/protein/XP_021312071.1?report=genbank&log$=prottop&blast_rank=88&RID=EK4FW9P8013","XP_021312071.1") |
| mitogen-activated protein kinase 9-3 [Cucumis sativus] | 345 | 345 | 93% | =HYPERLINK("https://www.ncbi.nlm.nih.gov/protein/AKP99751.1?report=genbank&log$=prottop&blast_rank=89&RID=EK4FW9P8013","AKP99751.1") |
| cyclin-dependent kinase C-2-like [Cucumis melo var. makuwa] | 345 | 345 | 92% | =HYPERLINK("https://www.ncbi.nlm.nih.gov/protein/KAA0040088.1?report=genbank&log$=prottop&blast_rank=90&RID=EK4FW9P8013","KAA0040088.1") |
| mitogen-activated protein kinase 8 isoform X1 [Sorghum bicolor] | 348 | 348 | 93% | =HYPERLINK("https://www.ncbi.nlm.nih.gov/protein/XP_021312070.1?report=genbank&log$=prottop&blast_rank=91&RID=EK4FW9P8013","XP_021312070.1") |
| cyclin-dependent kinase F-4 [Cucumis sativus] | 342 | 342 | 87% | =HYPERLINK("https://www.ncbi.nlm.nih.gov/protein/XP_004146120.2?report=genbank&log$=prottop&blast_rank=92&RID=EK4FW9P8013","XP_004146120.2") |
| cyclin-dependent kinase F-4 isoform X1 [Cucumis melo] | 342 | 342 | 90% | =HYPERLINK("https://www.ncbi.nlm.nih.gov/protein/XP_050942356.1?report=genbank&log$=prottop&blast_rank=93&RID=EK4FW9P8013","XP_050942356.1") |
| cell division control protein 2 homolog A [Lactuca sativa] | 336 | 336 | 83% | =HYPERLINK("https://www.ncbi.nlm.nih.gov/protein/XP_023771603.1?report=genbank&log$=prottop&blast_rank=94&RID=EK4FW9P8013","XP_023771603.1") |
| cyclin-dependent kinase F-4 isoform X2 [Cucumis melo] | 341 | 341 | 90% | =HYPERLINK("https://www.ncbi.nlm.nih.gov/protein/XP_050942358.1?report=genbank&log$=prottop&blast_rank=95&RID=EK4FW9P8013","XP_050942358.1") |
| cyclin-dependent kinase F-4-like isoform X1 [Cucumis melo var. makuwa] | 342 | 342 | 90% | =HYPERLINK("https://www.ncbi.nlm.nih.gov/protein/KAA0052994.1?report=genbank&log$=prottop&blast_rank=96&RID=EK4FW9P8013","KAA0052994.1") |
| unnamed protein product [Lactuca virosa] | 347 | 347 | 97% | =HYPERLINK("https://www.ncbi.nlm.nih.gov/protein/CAH1444610.1?report=genbank&log$=prottop&blast_rank=97&RID=EK4FW9P8013","CAH1444610.1") |
| unnamed protein product [Lactuca saligna] | 348 | 348 | 97% | =HYPERLINK("https://www.ncbi.nlm.nih.gov/protein/CAI9303849.1?report=genbank&log$=prottop&blast_rank=98&RID=EK4FW9P8013","CAI9303849.1") |
| mitogen-activated protein kinase 17 [Sorghum bicolor] | 346 | 346 | 94% | =HYPERLINK("https://www.ncbi.nlm.nih.gov/protein/XP_002440302.1?report=genbank&log$=prottop&blast_rank=99&RID=EK4FW9P8013","XP_002440302.1") |
| hypothetical protein BDA96_09G256600 [Sorghum bicolor] | 346 | 346 | 94% | =HYPERLINK("https://www.ncbi.nlm.nih.gov/protein/KAG0519349.1?report=genbank&log$=prottop&blast_rank=100&RID=EK4FW9P8013","KAG0519349.1") |
| cyclin-dependent kinase F-3 [Sorghum bicolor] | 340 | 340 | 89% | =HYPERLINK("https://www.ncbi.nlm.nih.gov/protein/XP_021307325.1?report=genbank&log$=prottop&blast_rank=101&RID=EK4FW9P8013","XP_021307325.1") |
| cell division control protein 2 homolog A isoform X1 [Cucumis melo] | 334 | 334 | 80% | =HYPERLINK("https://www.ncbi.nlm.nih.gov/protein/XP_008446776.1?report=genbank&log$=prottop&blast_rank=102&RID=EK4FW9P8013","XP_008446776.1") |
| mitogen-activated protein kinase homolog NTF6 [Cucumis sativus] | 337 | 337 | 95% | =HYPERLINK("https://www.ncbi.nlm.nih.gov/protein/XP_004148790.2?report=genbank&log$=prottop&blast_rank=103&RID=EK4FW9P8013","XP_004148790.2") |
| mitogen-activated protein kinase 9 [Lactuca sativa] | 344 | 344 | 97% | =HYPERLINK("https://www.ncbi.nlm.nih.gov/protein/XP_023756521.1?report=genbank&log$=prottop&blast_rank=104&RID=EK4FW9P8013","XP_023756521.1") |
| mitogen-activated protein kinase homolog NTF6 [Cucumis melo] | 336 | 336 | 95% | =HYPERLINK("https://www.ncbi.nlm.nih.gov/protein/XP_008465285.2?report=genbank&log$=prottop&blast_rank=105&RID=EK4FW9P8013","XP_008465285.2") |
| mitogen-activated protein kinase-like protein NTF6 [Cucumis melo var. makuwa] | 335 | 335 | 95% | =HYPERLINK("https://www.ncbi.nlm.nih.gov/protein/KAA0036980.1?report=genbank&log$=prottop&blast_rank=106&RID=EK4FW9P8013","KAA0036980.1") |
| unnamed protein product [Lactuca virosa] | 336 | 336 | 94% | =HYPERLINK("https://www.ncbi.nlm.nih.gov/protein/CAH1428175.1?report=genbank&log$=prottop&blast_rank=107&RID=EK4FW9P8013","CAH1428175.1") |
| unnamed protein product [Lactuca saligna] | 333 | 333 | 83% | =HYPERLINK("https://www.ncbi.nlm.nih.gov/protein/CAI9260217.1?report=genbank&log$=prottop&blast_rank=108&RID=EK4FW9P8013","CAI9260217.1") |
| cell division control protein 2 homolog A [Cucumis sativus] | 332 | 332 | 80% | =HYPERLINK("https://www.ncbi.nlm.nih.gov/protein/XP_004150484.1?report=genbank&log$=prottop&blast_rank=109&RID=EK4FW9P8013","XP_004150484.1") |
| cyclin-dependent kinase C-2 [Cucumis sativus] | 340 | 340 | 92% | =HYPERLINK("https://www.ncbi.nlm.nih.gov/protein/XP_011652872.2?report=genbank&log$=prottop&blast_rank=110&RID=EK4FW9P8013","XP_011652872.2") |
| cyclin-dependent kinase C-2-like isoform X1 [Cucumis melo] | 340 | 340 | 92% | =HYPERLINK("https://www.ncbi.nlm.nih.gov/protein/XP_008449858.1?report=genbank&log$=prottop&blast_rank=111&RID=EK4FW9P8013","XP_008449858.1") |
| LOW QUALITY PROTEIN: cyclin-dependent kinase C-2 [Cucumis sativus] | 340 | 340 | 92% | =HYPERLINK("https://www.ncbi.nlm.nih.gov/protein/XP_004149655.3?report=genbank&log$=prottop&blast_rank=112&RID=EK4FW9P8013","XP_004149655.3") |
| hypothetical protein BDA96_10G015700 [Sorghum bicolor] | 338 | 338 | 83% | =HYPERLINK("https://www.ncbi.nlm.nih.gov/protein/KAG0512464.1?report=genbank&log$=prottop&blast_rank=113&RID=EK4FW9P8013","KAG0512464.1") |
| cyclin-dependent kinase F-4 [Sorghum bicolor] | 338 | 338 | 83% | =HYPERLINK("https://www.ncbi.nlm.nih.gov/protein/XP_002436374.1?report=genbank&log$=prottop&blast_rank=114&RID=EK4FW9P8013","XP_002436374.1") |
| mitogen-activated protein kinase 13 [Cucumis sativus] | 333 | 333 | 95% | =HYPERLINK("https://www.ncbi.nlm.nih.gov/protein/AKP99752.1?report=genbank&log$=prottop&blast_rank=115&RID=EK4FW9P8013","AKP99752.1") |
| unnamed protein product [Lactuca saligna] | 333 | 333 | 84% | =HYPERLINK("https://www.ncbi.nlm.nih.gov/protein/CAI9279170.1?report=genbank&log$=prottop&blast_rank=116&RID=EK4FW9P8013","CAI9279170.1") |
| cyclin-dependent kinase F-4 isoform X3 [Lactuca sativa] | 336 | 336 | 85% | =HYPERLINK("https://www.ncbi.nlm.nih.gov/protein/XP_023734461.2?report=genbank&log$=prottop&blast_rank=117&RID=EK4FW9P8013","XP_023734461.2") |
| unnamed protein product [Lactuca saligna] | 339 | 339 | 92% | =HYPERLINK("https://www.ncbi.nlm.nih.gov/protein/CAI9285057.1?report=genbank&log$=prottop&blast_rank=118&RID=EK4FW9P8013","CAI9285057.1") |
| cyclin-dependent kinase F-4 isoform X1 [Lactuca sativa] | 336 | 336 | 85% | =HYPERLINK("https://www.ncbi.nlm.nih.gov/protein/XP_023734458.2?report=genbank&log$=prottop&blast_rank=119&RID=EK4FW9P8013","XP_023734458.2") |
| unnamed protein product [Lactuca saligna] | 336 | 336 | 85% | =HYPERLINK("https://www.ncbi.nlm.nih.gov/protein/CAI9284919.1?report=genbank&log$=prottop&blast_rank=120&RID=EK4FW9P8013","CAI9284919.1") |
| cyclin-dependent kinase F-4 isoform X4 [Lactuca sativa] | 336 | 336 | 85% | =HYPERLINK("https://www.ncbi.nlm.nih.gov/protein/XP_023734462.2?report=genbank&log$=prottop&blast_rank=121&RID=EK4FW9P8013","XP_023734462.2") |
| cell division control protein 2 homolog A [Lactuca sativa] | 330 | 330 | 80% | =HYPERLINK("https://www.ncbi.nlm.nih.gov/protein/XP_023730788.1?report=genbank&log$=prottop&blast_rank=122&RID=EK4FW9P8013","XP_023730788.1") |
| cyclin-dependent kinase F-4 isoform X2 [Lactuca sativa] | 336 | 336 | 85% | =HYPERLINK("https://www.ncbi.nlm.nih.gov/protein/XP_023734460.2?report=genbank&log$=prottop&blast_rank=123&RID=EK4FW9P8013","XP_023734460.2") |
| cyclin-dependent kinase C-2-like [Cucumis melo] | 338 | 338 | 92% | =HYPERLINK("https://www.ncbi.nlm.nih.gov/protein/XP_008443529.1?report=genbank&log$=prottop&blast_rank=124&RID=EK4FW9P8013","XP_008443529.1") |
| mitogen-activated protein kinase 19 isoform X2 [Cucumis sativus] | 336 | 336 | 97% | =HYPERLINK("https://www.ncbi.nlm.nih.gov/protein/XP_031740229.1?report=genbank&log$=prottop&blast_rank=125&RID=EK4FW9P8013","XP_031740229.1") |
| cyclin-dependent kinase C-2 [Lactuca sativa] | 337 | 337 | 92% | =HYPERLINK("https://www.ncbi.nlm.nih.gov/protein/XP_052620323.1?report=genbank&log$=prottop&blast_rank=126&RID=EK4FW9P8013","XP_052620323.1") |
| hypothetical protein LSAT_V11C500252170 [Lactuca sativa] | 337 | 337 | 92% | =HYPERLINK("https://www.ncbi.nlm.nih.gov/protein/KAJ0206655.1?report=genbank&log$=prottop&blast_rank=127&RID=EK4FW9P8013","KAJ0206655.1") |
| hypothetical protein Csa_018223 [Cucumis sativus] | 336 | 336 | 91% | =HYPERLINK("https://www.ncbi.nlm.nih.gov/protein/KAE8645962.1?report=genbank&log$=prottop&blast_rank=128&RID=EK4FW9P8013","KAE8645962.1") |
| cell division control protein 2 homolog [Sorghum bicolor] | 329 | 329 | 80% | =HYPERLINK("https://www.ncbi.nlm.nih.gov/protein/XP_021302193.1?report=genbank&log$=prottop&blast_rank=129&RID=EK4FW9P8013","XP_021302193.1") |
| mitogen-activated protein kinase 19-like isoform X2 [Cucumis melo] | 334 | 334 | 97% | =HYPERLINK("https://www.ncbi.nlm.nih.gov/protein/XP_008451775.1?report=genbank&log$=prottop&blast_rank=130&RID=EK4FW9P8013","XP_008451775.1") |
| unnamed protein product [Lactuca virosa] | 335 | 335 | 83% | =HYPERLINK("https://www.ncbi.nlm.nih.gov/protein/CAH1451740.1?report=genbank&log$=prottop&blast_rank=131&RID=EK4FW9P8013","CAH1451740.1") |
| unnamed protein product [Lactuca virosa] | 335 | 335 | 83% | =HYPERLINK("https://www.ncbi.nlm.nih.gov/protein/CAH1451741.1?report=genbank&log$=prottop&blast_rank=132&RID=EK4FW9P8013","CAH1451741.1") |
| unnamed protein product [Lactuca saligna] | 333 | 333 | 94% | =HYPERLINK("https://www.ncbi.nlm.nih.gov/protein/CAI9284146.1?report=genbank&log$=prottop&blast_rank=133&RID=EK4FW9P8013","CAI9284146.1") |
| unnamed protein product [Lactuca virosa] | 338 | 338 | 92% | =HYPERLINK("https://www.ncbi.nlm.nih.gov/protein/CAH1427233.1?report=genbank&log$=prottop&blast_rank=134&RID=EK4FW9P8013","CAH1427233.1") |
| mitogen-activated protein kinase 19 isoform X3 [Cucumis sativus] | 333 | 333 | 97% | =HYPERLINK("https://www.ncbi.nlm.nih.gov/protein/XP_031740230.1?report=genbank&log$=prottop&blast_rank=135&RID=EK4FW9P8013","XP_031740230.1") |
| mitogen-activated protein kinase 19-like isoform X4 [Cucumis melo] | 333 | 333 | 97% | =HYPERLINK("https://www.ncbi.nlm.nih.gov/protein/XP_008451777.1?report=genbank&log$=prottop&blast_rank=136&RID=EK4FW9P8013","XP_008451777.1") |
| mitogen-activated protein kinase 19 isoform X1 [Cucumis sativus] | 335 | 335 | 97% | =HYPERLINK("https://www.ncbi.nlm.nih.gov/protein/XP_004137245.3?report=genbank&log$=prottop&blast_rank=137&RID=EK4FW9P8013","XP_004137245.3") |
| mitogen-activated protein kinase 19 [Cucumis sativus] | 335 | 335 | 97% | =HYPERLINK("https://www.ncbi.nlm.nih.gov/protein/AKP99750.1?report=genbank&log$=prottop&blast_rank=138&RID=EK4FW9P8013","AKP99750.1") |
| cell division control protein 2 homolog 2 [Sorghum bicolor] | 328 | 328 | 80% | =HYPERLINK("https://www.ncbi.nlm.nih.gov/protein/CAZ96037.1?report=genbank&log$=prottop&blast_rank=139&RID=EK4FW9P8013","CAZ96037.1") |
| cyclin-dependent kinase A-2 [Sorghum bicolor] | 328 | 328 | 80% | =HYPERLINK("https://www.ncbi.nlm.nih.gov/protein/XP_002451427.1?report=genbank&log$=prottop&blast_rank=140&RID=EK4FW9P8013","XP_002451427.1") |
| mitogen-activated protein kinase 19-like isoform X1 [Cucumis melo] | 335 | 335 | 97% | =HYPERLINK("https://www.ncbi.nlm.nih.gov/protein/XP_008451774.1?report=genbank&log$=prottop&blast_rank=141&RID=EK4FW9P8013","XP_008451774.1") |
| mitogen-activated protein kinase 19-like isoform X3 [Cucumis melo] | 332 | 332 | 97% | =HYPERLINK("https://www.ncbi.nlm.nih.gov/protein/XP_008451776.1?report=genbank&log$=prottop&blast_rank=142&RID=EK4FW9P8013","XP_008451776.1") |
| cyclin-dependent kinase F-4 isoform X3 [Lactuca sativa] | 333 | 333 | 83% | =HYPERLINK("https://www.ncbi.nlm.nih.gov/protein/XP_042751602.1?report=genbank&log$=prottop&blast_rank=143&RID=EK4FW9P8013","XP_042751602.1") |
| unnamed protein product [Lactuca virosa] | 335 | 335 | 89% | =HYPERLINK("https://www.ncbi.nlm.nih.gov/protein/CAH1427463.1?report=genbank&log$=prottop&blast_rank=144&RID=EK4FW9P8013","CAH1427463.1") |
| cyclin-dependent kinase F-4 isoform X2 [Lactuca sativa] | 333 | 333 | 83% | =HYPERLINK("https://www.ncbi.nlm.nih.gov/protein/XP_023751005.1?report=genbank&log$=prottop&blast_rank=145&RID=EK4FW9P8013","XP_023751005.1") |
| cyclin-dependent kinase F-4 isoform X1 [Lactuca sativa] | 333 | 333 | 83% | =HYPERLINK("https://www.ncbi.nlm.nih.gov/protein/XP_023750997.1?report=genbank&log$=prottop&blast_rank=146&RID=EK4FW9P8013","XP_023750997.1") |
| hypothetical protein LSAT_V11C700367420 [Lactuca sativa] | 342 | 505 | 93% | =HYPERLINK("https://www.ncbi.nlm.nih.gov/protein/KAJ0194969.1?report=genbank&log$=prottop&blast_rank=147&RID=EK4FW9P8013","KAJ0194969.1") |
| hypothetical protein SORBI_3009G246300 [Sorghum bicolor] | 335 | 335 | 94% | =HYPERLINK("https://www.ncbi.nlm.nih.gov/protein/EES20009.1?report=genbank&log$=prottop&blast_rank=148&RID=EK4FW9P8013","EES20009.1") |
| hypothetical protein BDA96_09G261400 [Sorghum bicolor] | 335 | 335 | 94% | =HYPERLINK("https://www.ncbi.nlm.nih.gov/protein/KAG0519405.1?report=genbank&log$=prottop&blast_rank=149&RID=EK4FW9P8013","KAG0519405.1") |
| hypothetical protein SORBI_3001G107500 [Sorghum bicolor] | 332 | 332 | 91% | =HYPERLINK("https://www.ncbi.nlm.nih.gov/protein/EER93534.1?report=genbank&log$=prottop&blast_rank=150&RID=EK4FW9P8013","EER93534.1") |
| hypothetical protein BDA96_01G111900 [Sorghum bicolor] | 332 | 332 | 91% | =HYPERLINK("https://www.ncbi.nlm.nih.gov/protein/KAG0547806.1?report=genbank&log$=prottop&blast_rank=151&RID=EK4FW9P8013","KAG0547806.1") |
| cyclin-dependent kinase C-2-like [Cucumis melo var. makuwa] | 336 | 336 | 92% | =HYPERLINK("https://www.ncbi.nlm.nih.gov/protein/KAA0053658.1?report=genbank&log$=prottop&blast_rank=152&RID=EK4FW9P8013","KAA0053658.1") |
| hypothetical protein LSAT_V11C400171220 [Lactuca sativa] | 324 | 324 | 80% | =HYPERLINK("https://www.ncbi.nlm.nih.gov/protein/KAJ0210660.1?report=genbank&log$=prottop&blast_rank=153&RID=EK4FW9P8013","KAJ0210660.1") |
| uncharacterized protein LOC111877342 [Lactuca sativa] | 333 | 333 | 81% | =HYPERLINK("https://www.ncbi.nlm.nih.gov/protein/XP_023729631.1?report=genbank&log$=prottop&blast_rank=154&RID=EK4FW9P8013","XP_023729631.1") |
| cyclin-dependent kinase C-1 [Lactuca sativa] | 331 | 331 | 92% | =HYPERLINK("https://www.ncbi.nlm.nih.gov/protein/XP_023755370.1?report=genbank&log$=prottop&blast_rank=155&RID=EK4FW9P8013","XP_023755370.1") |
| mitogen-activated protein kinase 13 isoform X2 [Sorghum bicolor] | 330 | 330 | 95% | =HYPERLINK("https://www.ncbi.nlm.nih.gov/protein/XP_002453269.1?report=genbank&log$=prottop&blast_rank=156&RID=EK4FW9P8013","XP_002453269.1") |
| unnamed protein product [Lactuca virosa] | 331 | 331 | 92% | =HYPERLINK("https://www.ncbi.nlm.nih.gov/protein/CAH1446316.1?report=genbank&log$=prottop&blast_rank=157&RID=EK4FW9P8013","CAH1446316.1") |
| unnamed protein product [Lactuca saligna] | 331 | 331 | 92% | =HYPERLINK("https://www.ncbi.nlm.nih.gov/protein/CAI9301936.1?report=genbank&log$=prottop&blast_rank=158&RID=EK4FW9P8013","CAI9301936.1") |
| cyclin-dependent kinase G-2-like isoform X1 [Cucumis melo var. makuwa] | 337 | 337 | 95% | =HYPERLINK("https://www.ncbi.nlm.nih.gov/protein/KAA0034211.1?report=genbank&log$=prottop&blast_rank=159&RID=EK4FW9P8013","KAA0034211.1") |
| cyclin-dependent kinase G-2 isoform X1 [Cucumis sativus] | 337 | 337 | 95% | =HYPERLINK("https://www.ncbi.nlm.nih.gov/protein/XP_011655567.1?report=genbank&log$=prottop&blast_rank=160&RID=EK4FW9P8013","XP_011655567.1") |
| cyclin-dependent kinase G-2-like isoform X1 [Cucumis melo] | 337 | 337 | 95% | =HYPERLINK("https://www.ncbi.nlm.nih.gov/protein/XP_008446064.2?report=genbank&log$=prottop&blast_rank=161&RID=EK4FW9P8013","XP_008446064.2") |
| mitogen-activated protein kinase 10 [Sorghum bicolor] | 333 | 333 | 93% | =HYPERLINK("https://www.ncbi.nlm.nih.gov/protein/XP_021312656.1?report=genbank&log$=prottop&blast_rank=162&RID=EK4FW9P8013","XP_021312656.1") |
| hypothetical protein SORBI_3003G229400 [Sorghum bicolor] | 330 | 330 | 93% | =HYPERLINK("https://www.ncbi.nlm.nih.gov/protein/KXG32938.1?report=genbank&log$=prottop&blast_rank=163&RID=EK4FW9P8013","KXG32938.1") |
| cyclin-dependent kinase G-2 isoform X1 [Cucumis melo var. makuwa] | 336 | 336 | 98% | =HYPERLINK("https://www.ncbi.nlm.nih.gov/protein/KAA0061020.1?report=genbank&log$=prottop&blast_rank=164&RID=EK4FW9P8013","KAA0061020.1") |
| cyclin-dependent kinase G-2 isoform X1 [Cucumis melo] | 336 | 336 | 98% | =HYPERLINK("https://www.ncbi.nlm.nih.gov/protein/XP_008444433.2?report=genbank&log$=prottop&blast_rank=165&RID=EK4FW9P8013","XP_008444433.2") |
| hypothetical protein IC582_006732 [Cucumis melo] | 336 | 336 | 98% | =HYPERLINK("https://www.ncbi.nlm.nih.gov/protein/KAL0558165.1?report=genbank&log$=prottop&blast_rank=166&RID=EK4FW9P8013","KAL0558165.1") |
| unnamed protein product [Lactuca saligna] | 333 | 333 | 81% | =HYPERLINK("https://www.ncbi.nlm.nih.gov/protein/CAI9280500.1?report=genbank&log$=prottop&blast_rank=167&RID=EK4FW9P8013","CAI9280500.1") |
| mitogen-activated protein kinase 12 isoform X1 [Sorghum bicolor] | 329 | 329 | 94% | =HYPERLINK("https://www.ncbi.nlm.nih.gov/protein/XP_002437560.1?report=genbank&log$=prottop&blast_rank=168&RID=EK4FW9P8013","XP_002437560.1") |
| unnamed protein product [Lactuca virosa] | 332 | 332 | 81% | =HYPERLINK("https://www.ncbi.nlm.nih.gov/protein/CAH1423216.1?report=genbank&log$=prottop&blast_rank=169&RID=EK4FW9P8013","CAH1423216.1") |
| mitogen-activated protein kinase 12 isoform X2 [Sorghum bicolor] | 327 | 327 | 94% | =HYPERLINK("https://www.ncbi.nlm.nih.gov/protein/XP_021305170.1?report=genbank&log$=prottop&blast_rank=170&RID=EK4FW9P8013","XP_021305170.1") |
| hypothetical protein Csa_018659 [Cucumis sativus] | 334 | 334 | 98% | =HYPERLINK("https://www.ncbi.nlm.nih.gov/protein/KGN62365.1?report=genbank&log$=prottop&blast_rank=171&RID=EK4FW9P8013","KGN62365.1") |
| unnamed protein product [Lactuca virosa] | 330 | 330 | 94% | =HYPERLINK("https://www.ncbi.nlm.nih.gov/protein/CAH1449626.1?report=genbank&log$=prottop&blast_rank=172&RID=EK4FW9P8013","CAH1449626.1") |
| mitogen-activated protein kinase 10 [Sorghum bicolor] | 341 | 341 | 94% | =HYPERLINK("https://www.ncbi.nlm.nih.gov/protein/XP_002441579.2?report=genbank&log$=prottop&blast_rank=173&RID=EK4FW9P8013","XP_002441579.2") |
| cyclin-dependent kinase D-3-like [Cucumis melo var. makuwa] | 322 | 322 | 92% | =HYPERLINK("https://www.ncbi.nlm.nih.gov/protein/KAA0058120.1?report=genbank&log$=prottop&blast_rank=174&RID=EK4FW9P8013","KAA0058120.1") |
| cyclin-dependent kinase D-3-like [Cucumis melo var. makuwa] | 322 | 322 | 92% | =HYPERLINK("https://www.ncbi.nlm.nih.gov/protein/TYK28474.1?report=genbank&log$=prottop&blast_rank=175&RID=EK4FW9P8013","TYK28474.1") |
| mitogen-activated protein kinase 15 [Lactuca sativa] | 328 | 328 | 93% | =HYPERLINK("https://www.ncbi.nlm.nih.gov/protein/XP_023765622.1?report=genbank&log$=prottop&blast_rank=176&RID=EK4FW9P8013","XP_023765622.1") |
| mitogen-activated protein kinase 9 [Lactuca sativa] | 327 | 327 | 94% | =HYPERLINK("https://www.ncbi.nlm.nih.gov/protein/XP_023737458.1?report=genbank&log$=prottop&blast_rank=177&RID=EK4FW9P8013","XP_023737458.1") |
| serine/threonine-protein kinase MHK isoform X1 [Lactuca sativa] | 322 | 322 | 89% | =HYPERLINK("https://www.ncbi.nlm.nih.gov/protein/XP_023759200.1?report=genbank&log$=prottop&blast_rank=178&RID=EK4FW9P8013","XP_023759200.1") |
| cyclin-dependent kinase G-2 isoform X2 [Cucumis sativus] | 334 | 334 | 98% | =HYPERLINK("https://www.ncbi.nlm.nih.gov/protein/XP_031737297.1?report=genbank&log$=prottop&blast_rank=179&RID=EK4FW9P8013","XP_031737297.1") |
| serine/threonine-protein kinase MHK isoform X2 [Lactuca sativa] | 322 | 322 | 89% | =HYPERLINK("https://www.ncbi.nlm.nih.gov/protein/XP_042752965.1?report=genbank&log$=prottop&blast_rank=180&RID=EK4FW9P8013","XP_042752965.1") |
| unnamed protein product [Lactuca virosa] | 322 | 322 | 89% | =HYPERLINK("https://www.ncbi.nlm.nih.gov/protein/CAH1431953.1?report=genbank&log$=prottop&blast_rank=181&RID=EK4FW9P8013","CAH1431953.1") |
| hypothetical protein LSAT_V11C700381960 [Lactuca sativa] | 328 | 328 | 93% | =HYPERLINK("https://www.ncbi.nlm.nih.gov/protein/KAJ0195056.1?report=genbank&log$=prottop&blast_rank=182&RID=EK4FW9P8013","KAJ0195056.1") |
| unnamed protein product [Lactuca virosa] | 322 | 322 | 89% | =HYPERLINK("https://www.ncbi.nlm.nih.gov/protein/CAH1431952.1?report=genbank&log$=prottop&blast_rank=183&RID=EK4FW9P8013","CAH1431952.1") |
| cyclin-dependent kinase C-2 [Sorghum bicolor] | 325 | 325 | 91% | =HYPERLINK("https://www.ncbi.nlm.nih.gov/protein/XP_002439737.1?report=genbank&log$=prottop&blast_rank=184&RID=EK4FW9P8013","XP_002439737.1") |
| unnamed protein product [Lactuca virosa] | 325 | 325 | 93% | =HYPERLINK("https://www.ncbi.nlm.nih.gov/protein/CAH1449690.1?report=genbank&log$=prottop&blast_rank=185&RID=EK4FW9P8013","CAH1449690.1") |
| mitogen-activated protein kinase 9 [Lactuca sativa] | 325 | 325 | 94% | =HYPERLINK("https://www.ncbi.nlm.nih.gov/protein/XP_052625275.1?report=genbank&log$=prottop&blast_rank=186&RID=EK4FW9P8013","XP_052625275.1") |
| unnamed protein product [Lactuca virosa] | 325 | 325 | 93% | =HYPERLINK("https://www.ncbi.nlm.nih.gov/protein/CAH1449689.1?report=genbank&log$=prottop&blast_rank=187&RID=EK4FW9P8013","CAH1449689.1") |
| mitogen-activated protein kinase 9-like [Cucumis melo] | 328 | 328 | 95% | =HYPERLINK("https://www.ncbi.nlm.nih.gov/protein/XP_008453470.2?report=genbank&log$=prottop&blast_rank=188&RID=EK4FW9P8013","XP_008453470.2") |
| mitogen-activated protein kinase 14 [Lactuca sativa] | 322 | 322 | 93% | =HYPERLINK("https://www.ncbi.nlm.nih.gov/protein/XP_023752507.1?report=genbank&log$=prottop&blast_rank=189&RID=EK4FW9P8013","XP_023752507.1") |
| cyclin-dependent kinase D-3 [Cucumis sativus] | 321 | 321 | 92% | =HYPERLINK("https://www.ncbi.nlm.nih.gov/protein/XP_004137331.1?report=genbank&log$=prottop&blast_rank=190&RID=EK4FW9P8013","XP_004137331.1") |
| mitogen-activated protein kinase 9-like [Cucumis melo var. makuwa] | 328 | 328 | 95% | =HYPERLINK("https://www.ncbi.nlm.nih.gov/protein/TYK28484.1?report=genbank&log$=prottop&blast_rank=191&RID=EK4FW9P8013","TYK28484.1") |
| cyclin-dependent kinase G-1 [Sorghum bicolor] | 329 | 329 | 94% | =HYPERLINK("https://www.ncbi.nlm.nih.gov/protein/XP_002452405.1?report=genbank&log$=prottop&blast_rank=192&RID=EK4FW9P8013","XP_002452405.1") |
| cyclin-dependent kinase D-3 [Cucumis melo] | 321 | 321 | 92% | =HYPERLINK("https://www.ncbi.nlm.nih.gov/protein/XP_050936964.1?report=genbank&log$=prottop&blast_rank=193&RID=EK4FW9P8013","XP_050936964.1") |
| mitogen-activated protein kinase 9-like [Cucumis melo var. makuwa] | 328 | 328 | 95% | =HYPERLINK("https://www.ncbi.nlm.nih.gov/protein/KAA0058130.1?report=genbank&log$=prottop&blast_rank=194&RID=EK4FW9P8013","KAA0058130.1") |
| unnamed protein product [Lactuca virosa] | 326 | 326 | 93% | =HYPERLINK("https://www.ncbi.nlm.nih.gov/protein/CAH1450021.1?report=genbank&log$=prottop&blast_rank=195&RID=EK4FW9P8013","CAH1450021.1") |
| cyclin-dependent kinase D-1 isoform X2 [Sorghum bicolor] | 321 | 321 | 92% | =HYPERLINK("https://www.ncbi.nlm.nih.gov/protein/XP_021303461.1?report=genbank&log$=prottop&blast_rank=196&RID=EK4FW9P8013","XP_021303461.1") |
| unnamed protein product [Lactuca saligna] | 321 | 321 | 89% | =HYPERLINK("https://www.ncbi.nlm.nih.gov/protein/CAI9297447.1?report=genbank&log$=prottop&blast_rank=197&RID=EK4FW9P8013","CAI9297447.1") |
| unnamed protein product [Lactuca saligna] | 321 | 321 | 89% | =HYPERLINK("https://www.ncbi.nlm.nih.gov/protein/CAI9297446.1?report=genbank&log$=prottop&blast_rank=198&RID=EK4FW9P8013","CAI9297446.1") |
| mitogen-activated protein kinase 9 [Cucumis sativus] | 328 | 328 | 95% | =HYPERLINK("https://www.ncbi.nlm.nih.gov/protein/XP_011649120.1?report=genbank&log$=prottop&blast_rank=199&RID=EK4FW9P8013","XP_011649120.1") |
| cyclin-dependent kinase G-2 isoform X1 [Cucumis sativus] | 333 | 333 | 98% | =HYPERLINK("https://www.ncbi.nlm.nih.gov/protein/XP_004142940.2?report=genbank&log$=prottop&blast_rank=200&RID=EK4FW9P8013","XP_004142940.2") |
| unnamed protein product [Lactuca virosa] | 324 | 324 | 87% | =HYPERLINK("https://www.ncbi.nlm.nih.gov/protein/CAH1428556.1?report=genbank&log$=prottop&blast_rank=201&RID=EK4FW9P8013","CAH1428556.1") |
| probable serine/threonine-protein kinase At1g09600 isoform X1 [Cucumis sativus] | 327 | 327 | 91% | =HYPERLINK("https://www.ncbi.nlm.nih.gov/protein/XP_011656099.1?report=genbank&log$=prottop&blast_rank=202&RID=EK4FW9P8013","XP_011656099.1") |
| cyclin-dependent kinase G-2 isoform X1 [Lactuca sativa] | 329 | 329 | 91% | =HYPERLINK("https://www.ncbi.nlm.nih.gov/protein/XP_023769769.1?report=genbank&log$=prottop&blast_rank=203&RID=EK4FW9P8013","XP_023769769.1") |
| probable serine/threonine-protein kinase At1g54610 [Lactuca sativa] | 324 | 324 | 87% | =HYPERLINK("https://www.ncbi.nlm.nih.gov/protein/XP_023758066.1?report=genbank&log$=prottop&blast_rank=204&RID=EK4FW9P8013","XP_023758066.1") |
| unnamed protein product [Lactuca saligna] | 325 | 325 | 86% | =HYPERLINK("https://www.ncbi.nlm.nih.gov/protein/CAI9272670.1?report=genbank&log$=prottop&blast_rank=205&RID=EK4FW9P8013","CAI9272670.1") |
| mitogen-activated protein kinase 15-like [Lactuca sativa] | 318 | 318 | 94% | =HYPERLINK("https://www.ncbi.nlm.nih.gov/protein/XP_023750153.3?report=genbank&log$=prottop&blast_rank=206&RID=EK4FW9P8013","XP_023750153.3") |
| mitogen-activated protein kinase 20-like isoform X1 [Cucumis melo var. makuwa] | 326 | 326 | 96% | =HYPERLINK("https://www.ncbi.nlm.nih.gov/protein/KAA0054580.1?report=genbank&log$=prottop&blast_rank=207&RID=EK4FW9P8013","KAA0054580.1") |
| probable serine/threonine-protein kinase At1g54610 [Sorghum bicolor] | 323 | 323 | 89% | =HYPERLINK("https://www.ncbi.nlm.nih.gov/protein/XP_002465275.2?report=genbank&log$=prottop&blast_rank=208&RID=EK4FW9P8013","XP_002465275.2") |
| unnamed protein product [Lactuca saligna] | 325 | 325 | 94% | =HYPERLINK("https://www.ncbi.nlm.nih.gov/protein/CAI9299846.1?report=genbank&log$=prottop&blast_rank=209&RID=EK4FW9P8013","CAI9299846.1") |
| cyclin-dependent kinase B1-1 isoform X1 [Sorghum bicolor] | 315 | 315 | 81% | =HYPERLINK("https://www.ncbi.nlm.nih.gov/protein/XP_002441781.1?report=genbank&log$=prottop&blast_rank=210&RID=EK4FW9P8013","XP_002441781.1") |
| cyclin-dependent kinase D-1 isoform X1 [Sorghum bicolor] | 319 | 319 | 92% | =HYPERLINK("https://www.ncbi.nlm.nih.gov/protein/XP_002439746.1?report=genbank&log$=prottop&blast_rank=211&RID=EK4FW9P8013","XP_002439746.1") |
| mitogen-activated protein kinase 20-like isoform X1 [Cucumis melo] | 325 | 325 | 97% | =HYPERLINK("https://www.ncbi.nlm.nih.gov/protein/XP_050935591.1?report=genbank&log$=prottop&blast_rank=212&RID=EK4FW9P8013","XP_050935591.1") |
| mitogen-activated protein kinase 20-like isoform X2 [Cucumis melo] | 325 | 325 | 97% | =HYPERLINK("https://www.ncbi.nlm.nih.gov/protein/XP_008456353.2?report=genbank&log$=prottop&blast_rank=213&RID=EK4FW9P8013","XP_008456353.2") |
| unnamed protein product [Lactuca saligna] | 330 | 496 | 94% | =HYPERLINK("https://www.ncbi.nlm.nih.gov/protein/CAI9293966.1?report=genbank&log$=prottop&blast_rank=214&RID=EK4FW9P8013","CAI9293966.1") |
| putative serine/threonine-protein kinase [Cucumis melo var. makuwa] | 325 | 325 | 91% | =HYPERLINK("https://www.ncbi.nlm.nih.gov/protein/KAA0039258.1?report=genbank&log$=prottop&blast_rank=215&RID=EK4FW9P8013","KAA0039258.1") |
| unnamed protein product [Lactuca virosa] | 315 | 315 | 80% | =HYPERLINK("https://www.ncbi.nlm.nih.gov/protein/CAH1418321.1?report=genbank&log$=prottop&blast_rank=216&RID=EK4FW9P8013","CAH1418321.1") |
| hypothetical protein IC582_020491 [Cucumis melo] | 325 | 325 | 91% | =HYPERLINK("https://www.ncbi.nlm.nih.gov/protein/KAL0540484.1?report=genbank&log$=prottop&blast_rank=217&RID=EK4FW9P8013","KAL0540484.1") |
| cyclin-dependent kinase G-2 [Sorghum bicolor] | 327 | 327 | 93% | =HYPERLINK("https://www.ncbi.nlm.nih.gov/protein/XP_021317919.1?report=genbank&log$=prottop&blast_rank=218&RID=EK4FW9P8013","XP_021317919.1") |
| probable serine/threonine-protein kinase At1g09600 isoform X1 [Cucumis melo] | 325 | 325 | 91% | =HYPERLINK("https://www.ncbi.nlm.nih.gov/protein/XP_008459627.1?report=genbank&log$=prottop&blast_rank=219&RID=EK4FW9P8013","XP_008459627.1") |
| cyclin-dependent kinase G-2 isoform X2 [Lactuca sativa] | 325 | 325 | 91% | =HYPERLINK("https://www.ncbi.nlm.nih.gov/protein/XP_042753499.1?report=genbank&log$=prottop&blast_rank=220&RID=EK4FW9P8013","XP_042753499.1") |
| probable serine/threonine-protein kinase At1g54610 [Lactuca sativa] | 323 | 323 | 90% | =HYPERLINK("https://www.ncbi.nlm.nih.gov/protein/XP_023747177.1?report=genbank&log$=prottop&blast_rank=221&RID=EK4FW9P8013","XP_023747177.1") |
| hypothetical protein BDA96_06G142600 [Sorghum bicolor] | 327 | 327 | 93% | =HYPERLINK("https://www.ncbi.nlm.nih.gov/protein/KAG0526400.1?report=genbank&log$=prottop&blast_rank=222&RID=EK4FW9P8013","KAG0526400.1") |
| unnamed protein product [Lactuca saligna] | 314 | 314 | 80% | =HYPERLINK("https://www.ncbi.nlm.nih.gov/protein/CAI9287818.1?report=genbank&log$=prottop&blast_rank=223&RID=EK4FW9P8013","CAI9287818.1") |
| mitogen-activated protein kinase 20 isoform X1 [Cucumis sativus] | 324 | 324 | 93% | =HYPERLINK("https://www.ncbi.nlm.nih.gov/protein/XP_004143173.1?report=genbank&log$=prottop&blast_rank=224&RID=EK4FW9P8013","XP_004143173.1") |
| unnamed protein product [Lactuca saligna] | 322 | 322 | 87% | =HYPERLINK("https://www.ncbi.nlm.nih.gov/protein/CAI9280607.1?report=genbank&log$=prottop&blast_rank=225&RID=EK4FW9P8013","CAI9280607.1") |
| mitogen-activated protein kinase 20 isoform X2 [Cucumis sativus] | 323 | 323 | 93% | =HYPERLINK("https://www.ncbi.nlm.nih.gov/protein/XP_011657112.1?report=genbank&log$=prottop&blast_rank=226&RID=EK4FW9P8013","XP_011657112.1") |
| unnamed protein product [Lactuca saligna] | 328 | 328 | 91% | =HYPERLINK("https://www.ncbi.nlm.nih.gov/protein/CAI9299950.1?report=genbank&log$=prottop&blast_rank=227&RID=EK4FW9P8013","CAI9299950.1") |
| unnamed protein product [Lactuca virosa] | 322 | 322 | 90% | =HYPERLINK("https://www.ncbi.nlm.nih.gov/protein/CAH1423119.1?report=genbank&log$=prottop&blast_rank=228&RID=EK4FW9P8013","CAH1423119.1") |
| probable serine/threonine-protein kinase At1g54610 [Lactuca sativa] | 325 | 325 | 95% | =HYPERLINK("https://www.ncbi.nlm.nih.gov/protein/XP_023750106.1?report=genbank&log$=prottop&blast_rank=229&RID=EK4FW9P8013","XP_023750106.1") |
| unnamed protein product [Lactuca virosa] | 325 | 325 | 95% | =HYPERLINK("https://www.ncbi.nlm.nih.gov/protein/CAH1449930.1?report=genbank&log$=prottop&blast_rank=230&RID=EK4FW9P8013","CAH1449930.1") |
| unnamed protein product [Lactuca saligna] | 325 | 325 | 95% | =HYPERLINK("https://www.ncbi.nlm.nih.gov/protein/CAI9295110.1?report=genbank&log$=prottop&blast_rank=231&RID=EK4FW9P8013","CAI9295110.1") |
| unnamed protein product [Lactuca virosa] | 327 | 327 | 91% | =HYPERLINK("https://www.ncbi.nlm.nih.gov/protein/CAH1433841.1?report=genbank&log$=prottop&blast_rank=232&RID=EK4FW9P8013","CAH1433841.1") |
| cyclin-dependent kinase F-4 isoform X2 [Sorghum bicolor] | 317 | 317 | 90% | =HYPERLINK("https://www.ncbi.nlm.nih.gov/protein/XP_021316166.1?report=genbank&log$=prottop&blast_rank=233&RID=EK4FW9P8013","XP_021316166.1") |
| cyclin-dependent kinase F-4 [Lactuca sativa] | 312 | 312 | 81% | =HYPERLINK("https://www.ncbi.nlm.nih.gov/protein/XP_023772417.1?report=genbank&log$=prottop&blast_rank=234&RID=EK4FW9P8013","XP_023772417.1") |
| mitogen-activated protein kinase 15 isoform X1 [Cucumis melo] | 322 | 322 | 93% | =HYPERLINK("https://www.ncbi.nlm.nih.gov/protein/XP_008449538.1?report=genbank&log$=prottop&blast_rank=235&RID=EK4FW9P8013","XP_008449538.1") |
| mitogen-activated protein kinase 9 [Lactuca sativa] | 322 | 322 | 94% | =HYPERLINK("https://www.ncbi.nlm.nih.gov/protein/XP_023750800.1?report=genbank&log$=prottop&blast_rank=236&RID=EK4FW9P8013","XP_023750800.1") |
| mitogen-activated protein kinase 17-like isoform X2 [Cucumis melo] | 317 | 317 | 94% | =HYPERLINK("https://www.ncbi.nlm.nih.gov/protein/XP_008454747.1?report=genbank&log$=prottop&blast_rank=237&RID=EK4FW9P8013","XP_008454747.1") |
| probable serine/threonine-protein kinase At1g54610 [Lactuca sativa] | 322 | 322 | 86% | =HYPERLINK("https://www.ncbi.nlm.nih.gov/protein/XP_023771396.1?report=genbank&log$=prottop&blast_rank=238&RID=EK4FW9P8013","XP_023771396.1") |
| unnamed protein product [Lactuca virosa] | 322 | 322 | 94% | =HYPERLINK("https://www.ncbi.nlm.nih.gov/protein/CAH1433720.1?report=genbank&log$=prottop&blast_rank=239&RID=EK4FW9P8013","CAH1433720.1") |
| cyclin-dependent kinase F-4 isoform X1 [Sorghum bicolor] | 317 | 317 | 90% | =HYPERLINK("https://www.ncbi.nlm.nih.gov/protein/XP_002454430.1?report=genbank&log$=prottop&blast_rank=240&RID=EK4FW9P8013","XP_002454430.1") |
| hypothetical protein SORBI_3001G372900 [Sorghum bicolor] | 316 | 316 | 85% | =HYPERLINK("https://www.ncbi.nlm.nih.gov/protein/OQU92599.1?report=genbank&log$=prottop&blast_rank=241&RID=EK4FW9P8013","OQU92599.1") |
| mitogen-activated protein kinase 20 isoform X2 [Cucumis melo] | 320 | 320 | 93% | =HYPERLINK("https://www.ncbi.nlm.nih.gov/protein/XP_008449539.1?report=genbank&log$=prottop&blast_rank=242&RID=EK4FW9P8013","XP_008449539.1") |
| cyclin-dependent kinase C-3 isoform X1 [Sorghum bicolor] | 311 | 311 | 79% | =HYPERLINK("https://www.ncbi.nlm.nih.gov/protein/XP_002445594.1?report=genbank&log$=prottop&blast_rank=243&RID=EK4FW9P8013","XP_002445594.1") |
| unnamed protein product [Lactuca virosa] | 321 | 321 | 90% | =HYPERLINK("https://www.ncbi.nlm.nih.gov/protein/CAH1413168.1?report=genbank&log$=prottop&blast_rank=244&RID=EK4FW9P8013","CAH1413168.1") |
| mitogen-activated protein kinase 9-like isoform X1 [Cucumis melo] | 317 | 317 | 94% | =HYPERLINK("https://www.ncbi.nlm.nih.gov/protein/XP_008454742.1?report=genbank&log$=prottop&blast_rank=245&RID=EK4FW9P8013","XP_008454742.1") |
| unnamed protein product [Lactuca saligna] | 319 | 319 | 87% | =HYPERLINK("https://www.ncbi.nlm.nih.gov/protein/CAI9283728.1?report=genbank&log$=prottop&blast_rank=246&RID=EK4FW9P8013","CAI9283728.1") |
| hypothetical protein IC582_024352 [Cucumis melo] | 316 | 316 | 94% | =HYPERLINK("https://www.ncbi.nlm.nih.gov/protein/KAL0540122.1?report=genbank&log$=prottop&blast_rank=247&RID=EK4FW9P8013","KAL0540122.1") |
| probable serine/threonine-protein kinase At1g09600 isoform X2 [Cucumis sativus] | 317 | 317 | 90% | =HYPERLINK("https://www.ncbi.nlm.nih.gov/protein/XP_011656100.1?report=genbank&log$=prottop&blast_rank=248&RID=EK4FW9P8013","XP_011656100.1") |
| mitogen-activated protein kinase 9-like isoform X1 [Cucumis melo var. makuwa] | 316 | 316 | 94% | =HYPERLINK("https://www.ncbi.nlm.nih.gov/protein/KAA0056467.1?report=genbank&log$=prottop&blast_rank=249&RID=EK4FW9P8013","KAA0056467.1") |
| mitogen-activated protein kinase 20 isoform X2 [Cucumis sativus] | 318 | 318 | 93% | =HYPERLINK("https://www.ncbi.nlm.nih.gov/protein/XP_011657564.1?report=genbank&log$=prottop&blast_rank=250&RID=EK4FW9P8013","XP_011657564.1") |
| cyclin-dependent kinase B2-1 [Sorghum bicolor] | 311 | 311 | 79% | =HYPERLINK("https://www.ncbi.nlm.nih.gov/protein/XP_002438785.1?report=genbank&log$=prottop&blast_rank=251&RID=EK4FW9P8013","XP_002438785.1") |
| mitogen-activated protein kinase 17 isoform X2 [Cucumis sativus] | 314 | 314 | 94% | =HYPERLINK("https://www.ncbi.nlm.nih.gov/protein/XP_031741903.1?report=genbank&log$=prottop&blast_rank=252&RID=EK4FW9P8013","XP_031741903.1") |
| probable serine/threonine-protein kinase At1g09600 isoform X2 [Cucumis melo] | 316 | 316 | 90% | =HYPERLINK("https://www.ncbi.nlm.nih.gov/protein/XP_050946055.1?report=genbank&log$=prottop&blast_rank=253&RID=EK4FW9P8013","XP_050946055.1") |
| mitogen-activated protein kinase 20 isoform X3 [Cucumis melo] | 315 | 315 | 93% | =HYPERLINK("https://www.ncbi.nlm.nih.gov/protein/XP_008449540.1?report=genbank&log$=prottop&blast_rank=254&RID=EK4FW9P8013","XP_008449540.1") |
| hypothetical protein IC582_009998 [Cucumis melo] | 317 | 317 | 93% | =HYPERLINK("https://www.ncbi.nlm.nih.gov/protein/KAL0550930.1?report=genbank&log$=prottop&blast_rank=255&RID=EK4FW9P8013","KAL0550930.1") |
| cyclin-dependent kinase B2-1 [Sorghum bicolor] | 310 | 310 | 79% | =HYPERLINK("https://www.ncbi.nlm.nih.gov/protein/XP_002445888.1?report=genbank&log$=prottop&blast_rank=256&RID=EK4FW9P8013","XP_002445888.1") |
| mitogen-activated protein kinase 20 isoform X3 [Cucumis sativus] | 315 | 315 | 93% | =HYPERLINK("https://www.ncbi.nlm.nih.gov/protein/XP_011657566.1?report=genbank&log$=prottop&blast_rank=257&RID=EK4FW9P8013","XP_011657566.1") |
| mitogen-activated protein kinase 9 isoform X1 [Cucumis sativus] | 314 | 314 | 94% | =HYPERLINK("https://www.ncbi.nlm.nih.gov/protein/XP_011654436.1?report=genbank&log$=prottop&blast_rank=258&RID=EK4FW9P8013","XP_011654436.1") |
| mitogen-activated protein kinase 20 isoform X1 [Cucumis sativus] | 318 | 318 | 93% | =HYPERLINK("https://www.ncbi.nlm.nih.gov/protein/XP_004140142.1?report=genbank&log$=prottop&blast_rank=259&RID=EK4FW9P8013","XP_004140142.1") |
| unnamed protein product [Lactuca virosa] | 310 | 310 | 90% | =HYPERLINK("https://www.ncbi.nlm.nih.gov/protein/CAH1418327.1?report=genbank&log$=prottop&blast_rank=260&RID=EK4FW9P8013","CAH1418327.1") |
| mitogen-activated protein kinase 20-2 [Cucumis sativus] | 318 | 318 | 93% | =HYPERLINK("https://www.ncbi.nlm.nih.gov/protein/AKP99755.1?report=genbank&log$=prottop&blast_rank=261&RID=EK4FW9P8013","AKP99755.1") |
| hypothetical protein SORBI_3005G087300 [Sorghum bicolor] | 313 | 313 | 91% | =HYPERLINK("https://www.ncbi.nlm.nih.gov/protein/OQU83167.1?report=genbank&log$=prottop&blast_rank=262&RID=EK4FW9P8013","OQU83167.1") |
| cyclin-dependent kinase B2-2 [Cucumis melo] | 308 | 308 | 80% | =HYPERLINK("https://www.ncbi.nlm.nih.gov/protein/XP_008453019.1?report=genbank&log$=prottop&blast_rank=263&RID=EK4FW9P8013","XP_008453019.1") |
| cell division control protein 2 homolog C [Lactuca sativa] | 308 | 308 | 80% | =HYPERLINK("https://www.ncbi.nlm.nih.gov/protein/XP_023756404.1?report=genbank&log$=prottop&blast_rank=264&RID=EK4FW9P8013","XP_023756404.1") |
| unnamed protein product [Lactuca saligna] | 323 | 323 | 91% | =HYPERLINK("https://www.ncbi.nlm.nih.gov/protein/CAI9299951.1?report=genbank&log$=prottop&blast_rank=265&RID=EK4FW9P8013","CAI9299951.1") |
| probable serine/threonine-protein kinase At1g54610 isoform X2 [Sorghum bicolor] | 316 | 316 | 89% | =HYPERLINK("https://www.ncbi.nlm.nih.gov/protein/XP_021312377.1?report=genbank&log$=prottop&blast_rank=266&RID=EK4FW9P8013","XP_021312377.1") |
| probable serine/threonine-protein kinase At1g54610 isoform X1 [Sorghum bicolor] | 316 | 316 | 89% | =HYPERLINK("https://www.ncbi.nlm.nih.gov/protein/XP_021312376.1?report=genbank&log$=prottop&blast_rank=267&RID=EK4FW9P8013","XP_021312376.1") |
| cyclin-dependent kinase C-2 C [Lactuca sativa] | 317 | 317 | 88% | =HYPERLINK("https://www.ncbi.nlm.nih.gov/protein/XP_023745740.1?report=genbank&log$=prottop&blast_rank=268&RID=EK4FW9P8013","XP_023745740.1") |
| hypothetical protein SORBI_3001G372900 [Sorghum bicolor] | 316 | 316 | 87% | =HYPERLINK("https://www.ncbi.nlm.nih.gov/protein/OQU92600.1?report=genbank&log$=prottop&blast_rank=269&RID=EK4FW9P8013","OQU92600.1") |
| unnamed protein product [Lactuca saligna] | 317 | 317 | 88% | =HYPERLINK("https://www.ncbi.nlm.nih.gov/protein/CAI9283034.1?report=genbank&log$=prottop&blast_rank=270&RID=EK4FW9P8013","CAI9283034.1") |
| hypothetical protein BDA96_07G220600 [Sorghum bicolor] | 311 | 311 | 79% | =HYPERLINK("https://www.ncbi.nlm.nih.gov/protein/KAG0524543.1?report=genbank&log$=prottop&blast_rank=271&RID=EK4FW9P8013","KAG0524543.1") |
| unnamed protein product [Lactuca saligna] | 317 | 317 | 88% | =HYPERLINK("https://www.ncbi.nlm.nih.gov/protein/CAI9283035.1?report=genbank&log$=prottop&blast_rank=272&RID=EK4FW9P8013","CAI9283035.1") |
| cyclin-dependent kinase B2-2 [Cucumis sativus] | 308 | 308 | 80% | =HYPERLINK("https://www.ncbi.nlm.nih.gov/protein/XP_004145561.1?report=genbank&log$=prottop&blast_rank=273&RID=EK4FW9P8013","XP_004145561.1") |
| unnamed protein product [Lactuca virosa] | 323 | 323 | 91% | =HYPERLINK("https://www.ncbi.nlm.nih.gov/protein/CAH1433842.1?report=genbank&log$=prottop&blast_rank=274&RID=EK4FW9P8013","CAH1433842.1") |
| unnamed protein product [Lactuca virosa] | 317 | 317 | 89% | =HYPERLINK("https://www.ncbi.nlm.nih.gov/protein/CAH1450713.1?report=genbank&log$=prottop&blast_rank=275&RID=EK4FW9P8013","CAH1450713.1") |
| probable serine/threonine-protein kinase At1g54610 [Cucumis sativus] | 315 | 315 | 88% | =HYPERLINK("https://www.ncbi.nlm.nih.gov/protein/XP_004139211.1?report=genbank&log$=prottop&blast_rank=276&RID=EK4FW9P8013","XP_004139211.1") |
| putative serine/threonine-protein kinase [Cucumis melo var. makuwa] | 315 | 315 | 87% | =HYPERLINK("https://www.ncbi.nlm.nih.gov/protein/KAA0055352.1?report=genbank&log$=prottop&blast_rank=277&RID=EK4FW9P8013","KAA0055352.1") |
| probable serine/threonine-protein kinase At1g54610 isoform X2 [Cucumis melo] | 315 | 315 | 87% | =HYPERLINK("https://www.ncbi.nlm.nih.gov/protein/XP_008440049.2?report=genbank&log$=prottop&blast_rank=278&RID=EK4FW9P8013","XP_008440049.2") |
| probable serine/threonine-protein kinase At1g54610 [Lactuca sativa] | 320 | 320 | 90% | =HYPERLINK("https://www.ncbi.nlm.nih.gov/protein/XP_023751559.1?report=genbank&log$=prottop&blast_rank=279&RID=EK4FW9P8013","XP_023751559.1") |
| unnamed protein product [Lactuca saligna] | 316 | 316 | 89% | =HYPERLINK("https://www.ncbi.nlm.nih.gov/protein/CAI9274964.1?report=genbank&log$=prottop&blast_rank=280&RID=EK4FW9P8013","CAI9274964.1") |
| unnamed protein product [Lactuca virosa] | 316 | 316 | 89% | =HYPERLINK("https://www.ncbi.nlm.nih.gov/protein/CAH1415085.1?report=genbank&log$=prottop&blast_rank=281&RID=EK4FW9P8013","CAH1415085.1") |
| probable serine/threonine-protein kinase At1g54610 [Lactuca sativa] | 316 | 316 | 89% | =HYPERLINK("https://www.ncbi.nlm.nih.gov/protein/XP_023768657.1?report=genbank&log$=prottop&blast_rank=282&RID=EK4FW9P8013","XP_023768657.1") |
| cyclin-dependent kinase C-2 C [Lactuca sativa] | 317 | 317 | 90% | =HYPERLINK("https://www.ncbi.nlm.nih.gov/protein/XP_023761411.1?report=genbank&log$=prottop&blast_rank=283&RID=EK4FW9P8013","XP_023761411.1") |
| probable serine/threonine-protein kinase At1g54610 [Cucumis sativus] | 315 | 315 | 87% | =HYPERLINK("https://www.ncbi.nlm.nih.gov/protein/XP_004154002.1?report=genbank&log$=prottop&blast_rank=284&RID=EK4FW9P8013","XP_004154002.1") |
| probable serine/threonine-protein kinase At1g54610 [Cucumis melo] | 315 | 315 | 88% | =HYPERLINK("https://www.ncbi.nlm.nih.gov/protein/XP_008455661.1?report=genbank&log$=prottop&blast_rank=285&RID=EK4FW9P8013","XP_008455661.1") |
| unnamed protein product [Lactuca virosa] | 319 | 319 | 79% | =HYPERLINK("https://www.ncbi.nlm.nih.gov/protein/CAH1449624.1?report=genbank&log$=prottop&blast_rank=286&RID=EK4FW9P8013","CAH1449624.1") |
| cyclin-dependent kinase F-4 [Lactuca sativa] | 307 | 307 | 80% | =HYPERLINK("https://www.ncbi.nlm.nih.gov/protein/XP_023772421.1?report=genbank&log$=prottop&blast_rank=287&RID=EK4FW9P8013","XP_023772421.1") |
| probable serine/threonine-protein kinase At1g09600 isoform X2 [Lactuca sativa] | 316 | 316 | 89% | =HYPERLINK("https://www.ncbi.nlm.nih.gov/protein/XP_023762716.1?report=genbank&log$=prottop&blast_rank=288&RID=EK4FW9P8013","XP_023762716.1") |
| unnamed protein product [Lactuca saligna] | 320 | 320 | 90% | =HYPERLINK("https://www.ncbi.nlm.nih.gov/protein/CAI9280064.1?report=genbank&log$=prottop&blast_rank=289&RID=EK4FW9P8013","CAI9280064.1") |
| unnamed protein product [Lactuca saligna] | 306 | 306 | 80% | =HYPERLINK("https://www.ncbi.nlm.nih.gov/protein/CAI9287455.1?report=genbank&log$=prottop&blast_rank=290&RID=EK4FW9P8013","CAI9287455.1") |
| hypothetical protein LSAT_V11C500289100 [Lactuca sativa] | 312 | 312 | 80% | =HYPERLINK("https://www.ncbi.nlm.nih.gov/protein/KAJ0203239.1?report=genbank&log$=prottop&blast_rank=291&RID=EK4FW9P8013","KAJ0203239.1") |
| probable serine/threonine-protein kinase At1g54610 [Sorghum bicolor] | 318 | 318 | 86% | =HYPERLINK("https://www.ncbi.nlm.nih.gov/protein/XP_002452282.1?report=genbank&log$=prottop&blast_rank=292&RID=EK4FW9P8013","XP_002452282.1") |
| hypothetical protein BDA96_04G192500 [Sorghum bicolor] | 318 | 318 | 86% | =HYPERLINK("https://www.ncbi.nlm.nih.gov/protein/KAG0533423.1?report=genbank&log$=prottop&blast_rank=293&RID=EK4FW9P8013","KAG0533423.1") |
| hypothetical protein LSAT_V11C900496750 [Lactuca sativa] | 317 | 317 | 97% | =HYPERLINK("https://www.ncbi.nlm.nih.gov/protein/KAJ0188735.1?report=genbank&log$=prottop&blast_rank=294&RID=EK4FW9P8013","KAJ0188735.1") |
| probable serine/threonine-protein kinase At1g54610 [Cucumis melo] | 318 | 318 | 87% | =HYPERLINK("https://www.ncbi.nlm.nih.gov/protein/XP_016901915.1?report=genbank&log$=prottop&blast_rank=295&RID=EK4FW9P8013","XP_016901915.1") |
| hypothetical protein BDA96_07G013800 [Sorghum bicolor] | 320 | 320 | 87% | =HYPERLINK("https://www.ncbi.nlm.nih.gov/protein/KAG0522170.1?report=genbank&log$=prottop&blast_rank=296&RID=EK4FW9P8013","KAG0522170.1") |
| probable serine/threonine-protein kinase At1g54610 isoform X1 [Lactuca sativa] | 315 | 315 | 91% | =HYPERLINK("https://www.ncbi.nlm.nih.gov/protein/XP_023734616.1?report=genbank&log$=prottop&blast_rank=297&RID=EK4FW9P8013","XP_023734616.1") |
| probable serine/threonine-protein kinase At1g54610 isoform X2 [Lactuca sativa] | 315 | 315 | 91% | =HYPERLINK("https://www.ncbi.nlm.nih.gov/protein/XP_023734617.1?report=genbank&log$=prottop&blast_rank=298&RID=EK4FW9P8013","XP_023734617.1") |
| probable serine/threonine-protein kinase At1g54610 [Cucumis sativus] | 318 | 318 | 87% | =HYPERLINK("https://www.ncbi.nlm.nih.gov/protein/XP_004146248.1?report=genbank&log$=prottop&blast_rank=299&RID=EK4FW9P8013","XP_004146248.1") |
| probable serine/threonine-protein kinase At1g54610 [Lactuca sativa] | 315 | 315 | 89% | =HYPERLINK("https://www.ncbi.nlm.nih.gov/protein/XP_023739202.1?report=genbank&log$=prottop&blast_rank=300&RID=EK4FW9P8013","XP_023739202.1") |
| probable serine/threonine-protein kinase At1g54610 [Sorghum bicolor] | 314 | 314 | 89% | =HYPERLINK("https://www.ncbi.nlm.nih.gov/protein/XP_021308844.1?report=genbank&log$=prottop&blast_rank=301&RID=EK4FW9P8013","XP_021308844.1") |
| unnamed protein product [Lactuca saligna] | 309 | 309 | 85% | =HYPERLINK("https://www.ncbi.nlm.nih.gov/protein/CAI9287860.1?report=genbank&log$=prottop&blast_rank=302&RID=EK4FW9P8013","CAI9287860.1") |
| unnamed protein product [Lactuca saligna] | 315 | 315 | 89% | =HYPERLINK("https://www.ncbi.nlm.nih.gov/protein/CAI9284716.1?report=genbank&log$=prottop&blast_rank=303&RID=EK4FW9P8013","CAI9284716.1") |
| putative serine/threonine-protein kinase [Cucumis melo var. makuwa] | 318 | 318 | 87% | =HYPERLINK("https://www.ncbi.nlm.nih.gov/protein/TYK04978.1?report=genbank&log$=prottop&blast_rank=304&RID=EK4FW9P8013","TYK04978.1") |
| probable serine/threonine-protein kinase At1g09600 isoform X2 [Sorghum bicolor] | 316 | 316 | 90% | =HYPERLINK("https://www.ncbi.nlm.nih.gov/protein/XP_021301879.1?report=genbank&log$=prottop&blast_rank=305&RID=EK4FW9P8013","XP_021301879.1") |
| probable serine/threonine-protein kinase At1g09600 isoform X1 [Sorghum bicolor] | 316 | 316 | 90% | =HYPERLINK("https://www.ncbi.nlm.nih.gov/protein/XP_002441983.2?report=genbank&log$=prottop&blast_rank=306&RID=EK4FW9P8013","XP_002441983.2") |
| hypothetical protein BDA96_05G090200 [Sorghum bicolor] | 314 | 314 | 91% | =HYPERLINK("https://www.ncbi.nlm.nih.gov/protein/KAG0529339.1?report=genbank&log$=prottop&blast_rank=307&RID=EK4FW9P8013","KAG0529339.1") |
| probable serine/threonine-protein kinase At1g54610 [Cucumis sativus] | 318 | 318 | 87% | =HYPERLINK("https://www.ncbi.nlm.nih.gov/protein/XP_011656948.1?report=genbank&log$=prottop&blast_rank=308&RID=EK4FW9P8013","XP_011656948.1") |
| probable serine/threonine-protein kinase At1g54610 isoform X2 [Cucumis melo] | 318 | 318 | 87% | =HYPERLINK("https://www.ncbi.nlm.nih.gov/protein/XP_008448300.2?report=genbank&log$=prottop&blast_rank=309&RID=EK4FW9P8013","XP_008448300.2") |
| probable serine/threonine-protein kinase At1g54610 isoform X3 [Cucumis melo] | 318 | 318 | 87% | =HYPERLINK("https://www.ncbi.nlm.nih.gov/protein/XP_050947534.1?report=genbank&log$=prottop&blast_rank=310&RID=EK4FW9P8013","XP_050947534.1") |
| probable serine/threonine-protein kinase At1g54610 isoform X1 [Cucumis melo] | 318 | 318 | 87% | =HYPERLINK("https://www.ncbi.nlm.nih.gov/protein/XP_008448298.2?report=genbank&log$=prottop&blast_rank=311&RID=EK4FW9P8013","XP_008448298.2") |
| unnamed protein product [Lactuca virosa] | 305 | 305 | 80% | =HYPERLINK("https://www.ncbi.nlm.nih.gov/protein/CAH1418326.1?report=genbank&log$=prottop&blast_rank=312&RID=EK4FW9P8013","CAH1418326.1") |
| probable serine/threonine-protein kinase At1g54610 [Cucumis melo] | 313 | 313 | 86% | =HYPERLINK("https://www.ncbi.nlm.nih.gov/protein/XP_008450739.2?report=genbank&log$=prottop&blast_rank=313&RID=EK4FW9P8013","XP_008450739.2") |
| cyclin-dependent kinase D-3 [Lactuca sativa] | 308 | 308 | 88% | =HYPERLINK("https://www.ncbi.nlm.nih.gov/protein/XP_023736912.1?report=genbank&log$=prottop&blast_rank=314&RID=EK4FW9P8013","XP_023736912.1") |
| unnamed protein product [Lactuca saligna] | 312 | 312 | 89% | =HYPERLINK("https://www.ncbi.nlm.nih.gov/protein/CAI9276951.1?report=genbank&log$=prottop&blast_rank=315&RID=EK4FW9P8013","CAI9276951.1") |
| unnamed protein product [Lactuca saligna] | 305 | 305 | 81% | =HYPERLINK("https://www.ncbi.nlm.nih.gov/protein/CAI9296300.1?report=genbank&log$=prottop&blast_rank=316&RID=EK4FW9P8013","CAI9296300.1") |
| unnamed protein product [Lactuca saligna] | 305 | 305 | 80% | =HYPERLINK("https://www.ncbi.nlm.nih.gov/protein/CAI9287876.1?report=genbank&log$=prottop&blast_rank=317&RID=EK4FW9P8013","CAI9287876.1") |
| probable serine/threonine-protein kinase At1g54610 [Cucumis sativus] | 313 | 313 | 86% | =HYPERLINK("https://www.ncbi.nlm.nih.gov/protein/XP_004135651.1?report=genbank&log$=prottop&blast_rank=318&RID=EK4FW9P8013","XP_004135651.1") |
| unnamed protein product [Lactuca saligna] | 307 | 307 | 88% | =HYPERLINK("https://www.ncbi.nlm.nih.gov/protein/CAI9281916.1?report=genbank&log$=prottop&blast_rank=319&RID=EK4FW9P8013","CAI9281916.1") |
| probable serine/threonine-protein kinase At1g54610 [Cucumis melo] | 313 | 313 | 87% | =HYPERLINK("https://www.ncbi.nlm.nih.gov/protein/XP_008448295.2?report=genbank&log$=prottop&blast_rank=320&RID=EK4FW9P8013","XP_008448295.2") |
| probable serine/threonine-protein kinase At1g54610 [Cucumis sativus] | 314 | 314 | 87% | =HYPERLINK("https://www.ncbi.nlm.nih.gov/protein/XP_011656950.1?report=genbank&log$=prottop&blast_rank=321&RID=EK4FW9P8013","XP_011656950.1") |
| probable serine/threonine-protein kinase At1g54610 [Sorghum bicolor] | 316 | 316 | 87% | =HYPERLINK("https://www.ncbi.nlm.nih.gov/protein/XP_002457168.1?report=genbank&log$=prottop&blast_rank=322&RID=EK4FW9P8013","XP_002457168.1") |
| putative serine/threonine-protein kinase [Cucumis melo var. makuwa] | 313 | 313 | 86% | =HYPERLINK("https://www.ncbi.nlm.nih.gov/protein/TYK10183.1?report=genbank&log$=prottop&blast_rank=323&RID=EK4FW9P8013","TYK10183.1") |
| cyclin-dependent kinase E-1 [Sorghum bicolor] | 309 | 309 | 94% | =HYPERLINK("https://www.ncbi.nlm.nih.gov/protein/XP_002464856.1?report=genbank&log$=prottop&blast_rank=324&RID=EK4FW9P8013","XP_002464856.1") |
| unnamed protein product [Lactuca virosa] | 313 | 313 | 89% | =HYPERLINK("https://www.ncbi.nlm.nih.gov/protein/CAH1427656.1?report=genbank&log$=prottop&blast_rank=325&RID=EK4FW9P8013","CAH1427656.1") |
| unnamed protein product [Lactuca virosa] | 303 | 303 | 81% | =HYPERLINK("https://www.ncbi.nlm.nih.gov/protein/CAH1430926.1?report=genbank&log$=prottop&blast_rank=326&RID=EK4FW9P8013","CAH1430926.1") |
| hypothetical protein LSAT_V11C300123360 [Lactuca sativa] | 314 | 314 | 94% | =HYPERLINK("https://www.ncbi.nlm.nih.gov/protein/KAJ0216230.1?report=genbank&log$=prottop&blast_rank=327&RID=EK4FW9P8013","KAJ0216230.1") |
| cyclin-dependent kinase B2-2 isoform X2 [Lactuca sativa] | 303 | 303 | 81% | =HYPERLINK("https://www.ncbi.nlm.nih.gov/protein/XP_023730725.1?report=genbank&log$=prottop&blast_rank=328&RID=EK4FW9P8013","XP_023730725.1") |
| probable serine/threonine-protein kinase At1g54610 [Cucumis melo] | 312 | 312 | 90% | =HYPERLINK("https://www.ncbi.nlm.nih.gov/protein/XP_008438801.1?report=genbank&log$=prottop&blast_rank=329&RID=EK4FW9P8013","XP_008438801.1") |
| cyclin-dependent kinase C-2-like isoform X2 [Cucumis melo] | 309 | 309 | 87% | =HYPERLINK("https://www.ncbi.nlm.nih.gov/protein/XP_050940964.1?report=genbank&log$=prottop&blast_rank=330&RID=EK4FW9P8013","XP_050940964.1") |
| probable serine/threonine-protein kinase At1g54610 [Cucumis sativus] | 311 | 311 | 90% | =HYPERLINK("https://www.ncbi.nlm.nih.gov/protein/XP_004134185.1?report=genbank&log$=prottop&blast_rank=331&RID=EK4FW9P8013","XP_004134185.1") |
| probable serine/threonine-protein kinase At1g09600 isoform X1 [Lactuca sativa] | 313 | 313 | 89% | =HYPERLINK("https://www.ncbi.nlm.nih.gov/protein/XP_023762704.1?report=genbank&log$=prottop&blast_rank=332&RID=EK4FW9P8013","XP_023762704.1") |
| cyclin-dependent kinase C-3 isoform X2 [Sorghum bicolor] | 303 | 303 | 79% | =HYPERLINK("https://www.ncbi.nlm.nih.gov/protein/XP_021320508.1?report=genbank&log$=prottop&blast_rank=333&RID=EK4FW9P8013","XP_021320508.1") |
| unnamed protein product [Lactuca virosa] | 313 | 313 | 89% | =HYPERLINK("https://www.ncbi.nlm.nih.gov/protein/CAH1450712.1?report=genbank&log$=prottop&blast_rank=334&RID=EK4FW9P8013","CAH1450712.1") |
| putative serine/threonine-protein kinase [Cucumis melo var. makuwa] | 311 | 311 | 88% | =HYPERLINK("https://www.ncbi.nlm.nih.gov/protein/KAA0063637.1?report=genbank&log$=prottop&blast_rank=335&RID=EK4FW9P8013","KAA0063637.1") |
| putative serine/threonine-protein kinase [Cucumis melo var. makuwa] | 311 | 311 | 87% | =HYPERLINK("https://www.ncbi.nlm.nih.gov/protein/TYK04977.1?report=genbank&log$=prottop&blast_rank=336&RID=EK4FW9P8013","TYK04977.1") |
| unnamed protein product [Lactuca virosa] | 312 | 312 | 88% | =HYPERLINK("https://www.ncbi.nlm.nih.gov/protein/CAH1421007.1?report=genbank&log$=prottop&blast_rank=337&RID=EK4FW9P8013","CAH1421007.1") |
| shaggy-related protein kinase alpha-like [Sorghum bicolor] | 306 | 306 | 84% | =HYPERLINK("https://www.ncbi.nlm.nih.gov/protein/XP_021312947.1?report=genbank&log$=prottop&blast_rank=338&RID=EK4FW9P8013","XP_021312947.1") |
| probable serine/threonine-protein kinase At1g54610 [Cucumis melo] | 311 | 311 | 89% | =HYPERLINK("https://www.ncbi.nlm.nih.gov/protein/XP_008440094.1?report=genbank&log$=prottop&blast_rank=339&RID=EK4FW9P8013","XP_008440094.1") |
| unnamed protein product [Lactuca saligna] | 314 | 314 | 91% | =HYPERLINK("https://www.ncbi.nlm.nih.gov/protein/CAI9282181.1?report=genbank&log$=prottop&blast_rank=340&RID=EK4FW9P8013","CAI9282181.1") |
| mitogen-activated protein kinase 15 [Lactuca sativa] | 318 | 487 | 93% | =HYPERLINK("https://www.ncbi.nlm.nih.gov/protein/XP_052626926.1?report=genbank&log$=prottop&blast_rank=341&RID=EK4FW9P8013","XP_052626926.1") |
| unnamed protein product [Lactuca saligna] | 312 | 312 | 89% | =HYPERLINK("https://www.ncbi.nlm.nih.gov/protein/CAI9266021.1?report=genbank&log$=prottop&blast_rank=342&RID=EK4FW9P8013","CAI9266021.1") |
| probable serine/threonine-protein kinase At1g09600 [Lactuca sativa] | 311 | 311 | 90% | =HYPERLINK("https://www.ncbi.nlm.nih.gov/protein/XP_023764954.1?report=genbank&log$=prottop&blast_rank=343&RID=EK4FW9P8013","XP_023764954.1") |
| unnamed protein product [Lactuca saligna] | 305 | 305 | 85% | =HYPERLINK("https://www.ncbi.nlm.nih.gov/protein/CAI9287862.1?report=genbank&log$=prottop&blast_rank=344&RID=EK4FW9P8013","CAI9287862.1") |
| cyclin-dependent kinase B1-1 [Lactuca sativa] | 301 | 301 | 80% | =HYPERLINK("https://www.ncbi.nlm.nih.gov/protein/XP_023743584.1?report=genbank&log$=prottop&blast_rank=345&RID=EK4FW9P8013","XP_023743584.1") |
| unnamed protein product [Lactuca virosa] | 314 | 314 | 89% | =HYPERLINK("https://www.ncbi.nlm.nih.gov/protein/CAH1443720.1?report=genbank&log$=prottop&blast_rank=346&RID=EK4FW9P8013","CAH1443720.1") |
| unnamed protein product [Lactuca saligna] | 314 | 314 | 85% | =HYPERLINK("https://www.ncbi.nlm.nih.gov/protein/CAI9282182.1?report=genbank&log$=prottop&blast_rank=347&RID=EK4FW9P8013","CAI9282182.1") |
| probable serine/threonine-protein kinase At1g54610 isoform X2 [Sorghum bicolor] | 314 | 314 | 89% | =HYPERLINK("https://www.ncbi.nlm.nih.gov/protein/XP_021308983.1?report=genbank&log$=prottop&blast_rank=348&RID=EK4FW9P8013","XP_021308983.1") |
| cell division control protein 2 homolog A isoform X2 [Cucumis melo] | 300 | 300 | 73% | =HYPERLINK("https://www.ncbi.nlm.nih.gov/protein/XP_050946974.1?report=genbank&log$=prottop&blast_rank=349&RID=EK4FW9P8013","XP_050946974.1") |
| unnamed protein product [Lactuca saligna] | 301 | 301 | 80% | =HYPERLINK("https://www.ncbi.nlm.nih.gov/protein/CAI9287470.1?report=genbank&log$=prottop&blast_rank=350&RID=EK4FW9P8013","CAI9287470.1") |
| probable serine/threonine-protein kinase At1g54610 isoform X1 [Sorghum bicolor] | 314 | 314 | 89% | =HYPERLINK("https://www.ncbi.nlm.nih.gov/protein/XP_002463130.1?report=genbank&log$=prottop&blast_rank=351&RID=EK4FW9P8013","XP_002463130.1") |
| unnamed protein product [Lactuca saligna] | 310 | 310 | 90% | =HYPERLINK("https://www.ncbi.nlm.nih.gov/protein/CAI9267174.1?report=genbank&log$=prottop&blast_rank=352&RID=EK4FW9P8013","CAI9267174.1") |
| unnamed protein product [Lactuca saligna] | 314 | 314 | 89% | =HYPERLINK("https://www.ncbi.nlm.nih.gov/protein/CAI9304421.1?report=genbank&log$=prottop&blast_rank=353&RID=EK4FW9P8013","CAI9304421.1") |
| unnamed protein product [Lactuca virosa] | 301 | 301 | 80% | =HYPERLINK("https://www.ncbi.nlm.nih.gov/protein/CAH1423985.1?report=genbank&log$=prottop&blast_rank=354&RID=EK4FW9P8013","CAH1423985.1") |
| unnamed protein product [Lactuca virosa] | 323 | 323 | 90% | =HYPERLINK("https://www.ncbi.nlm.nih.gov/protein/CAH1423622.1?report=genbank&log$=prottop&blast_rank=355&RID=EK4FW9P8013","CAH1423622.1") |
| probable serine/threonine-protein kinase At1g54610 [Lactuca sativa] | 313 | 313 | 89% | =HYPERLINK("https://www.ncbi.nlm.nih.gov/protein/XP_023731218.1?report=genbank&log$=prottop&blast_rank=356&RID=EK4FW9P8013","XP_023731218.1") |
| probable serine/threonine-protein kinase At1g54610 isoform X1 [Cucumis melo] | 310 | 310 | 85% | =HYPERLINK("https://www.ncbi.nlm.nih.gov/protein/XP_050936057.1?report=genbank&log$=prottop&blast_rank=357&RID=EK4FW9P8013","XP_050936057.1") |
| putative serine/threonine-protein kinase [Cucumis melo var. makuwa] | 307 | 307 | 85% | =HYPERLINK("https://www.ncbi.nlm.nih.gov/protein/TYJ99278.1?report=genbank&log$=prottop&blast_rank=358&RID=EK4FW9P8013","TYJ99278.1") |
| protein IMPAIRED IN BABA-INDUCED STERILITY 1 [Lactuca sativa] | 312 | 312 | 89% | =HYPERLINK("https://www.ncbi.nlm.nih.gov/protein/XP_023768152.1?report=genbank&log$=prottop&blast_rank=359&RID=EK4FW9P8013","XP_023768152.1") |
| unnamed protein product [Lactuca virosa] | 312 | 312 | 88% | =HYPERLINK("https://www.ncbi.nlm.nih.gov/protein/CAH1442966.1?report=genbank&log$=prottop&blast_rank=360&RID=EK4FW9P8013","CAH1442966.1") |
| hypothetical protein LSAT_V11C300138260 [Lactuca sativa] | 306 | 306 | 87% | =HYPERLINK("https://www.ncbi.nlm.nih.gov/protein/KAJ0218732.1?report=genbank&log$=prottop&blast_rank=361&RID=EK4FW9P8013","KAJ0218732.1") |
| unnamed protein product [Lactuca saligna] | 312 | 312 | 88% | =HYPERLINK("https://www.ncbi.nlm.nih.gov/protein/CAI9266811.1?report=genbank&log$=prottop&blast_rank=362&RID=EK4FW9P8013","CAI9266811.1") |
| probable serine/threonine-protein kinase At1g54610 [Cucumis sativus] | 309 | 309 | 89% | =HYPERLINK("https://www.ncbi.nlm.nih.gov/protein/XP_004142044.1?report=genbank&log$=prottop&blast_rank=363&RID=EK4FW9P8013","XP_004142044.1") |
| shaggy-related protein kinase zeta isoform X2 [Cucumis melo] | 302 | 302 | 89% | =HYPERLINK("https://www.ncbi.nlm.nih.gov/protein/XP_050947465.1?report=genbank&log$=prottop&blast_rank=364&RID=EK4FW9P8013","XP_050947465.1") |
| putative serine/threonine-protein kinase [Cucumis melo var. makuwa] | 311 | 311 | 88% | =HYPERLINK("https://www.ncbi.nlm.nih.gov/protein/TYJ98912.1?report=genbank&log$=prottop&blast_rank=365&RID=EK4FW9P8013","TYJ98912.1") |
| cyclin-dependent kinase B2-2 [Lactuca sativa] | 300 | 300 | 80% | =HYPERLINK("https://www.ncbi.nlm.nih.gov/protein/XP_023730721.1?report=genbank&log$=prottop&blast_rank=366&RID=EK4FW9P8013","XP_023730721.1") |
| unnamed protein product [Lactuca virosa] | 306 | 306 | 94% | =HYPERLINK("https://www.ncbi.nlm.nih.gov/protein/CAH1414139.1?report=genbank&log$=prottop&blast_rank=367&RID=EK4FW9P8013","CAH1414139.1") |
| unnamed protein product [Lactuca saligna] | 300 | 300 | 80% | =HYPERLINK("https://www.ncbi.nlm.nih.gov/protein/CAI9296299.1?report=genbank&log$=prottop&blast_rank=368&RID=EK4FW9P8013","CAI9296299.1") |
| cyclin-dependent kinase F-4 [Lactuca sativa] | 305 | 305 | 93% | =HYPERLINK("https://www.ncbi.nlm.nih.gov/protein/XP_023760592.1?report=genbank&log$=prottop&blast_rank=369&RID=EK4FW9P8013","XP_023760592.1") |
| shaggy-related protein kinase zeta isoform X2 [Cucumis sativus] | 301 | 301 | 89% | =HYPERLINK("https://www.ncbi.nlm.nih.gov/protein/XP_031743448.1?report=genbank&log$=prottop&blast_rank=370&RID=EK4FW9P8013","XP_031743448.1") |
| mitogen-activated protein kinase 8 [Lactuca sativa] | 311 | 474 | 80% | =HYPERLINK("https://www.ncbi.nlm.nih.gov/protein/XP_023750137.2?report=genbank&log$=prottop&blast_rank=371&RID=EK4FW9P8013","XP_023750137.2") |
| putative serine/threonine-protein kinase [Cucumis melo var. makuwa] | 307 | 307 | 90% | =HYPERLINK("https://www.ncbi.nlm.nih.gov/protein/KAA0049439.1?report=genbank&log$=prottop&blast_rank=372&RID=EK4FW9P8013","KAA0049439.1") |
| cell division control protein 2 homolog C [Cucumis melo] | 298 | 298 | 80% | =HYPERLINK("https://www.ncbi.nlm.nih.gov/protein/XP_008463312.1?report=genbank&log$=prottop&blast_rank=373&RID=EK4FW9P8013","XP_008463312.1") |
| protein IMPAIRED IN BABA-INDUCED STERILITY 1 [Cucumis melo] | 311 | 311 | 88% | =HYPERLINK("https://www.ncbi.nlm.nih.gov/protein/XP_008445783.1?report=genbank&log$=prottop&blast_rank=374&RID=EK4FW9P8013","XP_008445783.1") |
| shaggy-related protein kinase eta isoform X1 [Cucumis melo] | 302 | 302 | 89% | =HYPERLINK("https://www.ncbi.nlm.nih.gov/protein/XP_008448253.1?report=genbank&log$=prottop&blast_rank=375&RID=EK4FW9P8013","XP_008448253.1") |
| glycogen synthase kinase-3 homolog MsK-3 [Sorghum bicolor] | 301 | 301 | 88% | =HYPERLINK("https://www.ncbi.nlm.nih.gov/protein/XP_002455378.1?report=genbank&log$=prottop&blast_rank=376&RID=EK4FW9P8013","XP_002455378.1") |
| shaggy-related protein kinase eta isoform X1 [Cucumis sativus] | 302 | 302 | 89% | =HYPERLINK("https://www.ncbi.nlm.nih.gov/protein/XP_004149276.1?report=genbank&log$=prottop&blast_rank=377&RID=EK4FW9P8013","XP_004149276.1") |
| cyclin-dependent kinase E-1 isoform X1 [Cucumis melo] | 304 | 304 | 91% | =HYPERLINK("https://www.ncbi.nlm.nih.gov/protein/XP_008442225.2?report=genbank&log$=prottop&blast_rank=378&RID=EK4FW9P8013","XP_008442225.2") |
| unnamed protein product [Lactuca virosa] | 309 | 309 | 91% | =HYPERLINK("https://www.ncbi.nlm.nih.gov/protein/CAH1442635.1?report=genbank&log$=prottop&blast_rank=379&RID=EK4FW9P8013","CAH1442635.1") |
| unnamed protein product [Lactuca saligna] | 318 | 487 | 93% | =HYPERLINK("https://www.ncbi.nlm.nih.gov/protein/CAI9268204.1?report=genbank&log$=prottop&blast_rank=380&RID=EK4FW9P8013","CAI9268204.1") |
| cyclin-dependent kinase E-1 [Cucumis melo var. makuwa] | 303 | 303 | 91% | =HYPERLINK("https://www.ncbi.nlm.nih.gov/protein/KAA0041158.1?report=genbank&log$=prottop&blast_rank=381&RID=EK4FW9P8013","KAA0041158.1") |
| unnamed protein product [Lactuca saligna] | 318 | 487 | 93% | =HYPERLINK("https://www.ncbi.nlm.nih.gov/protein/CAI9268203.1?report=genbank&log$=prottop&blast_rank=382&RID=EK4FW9P8013","CAI9268203.1") |
| shaggy-related protein kinase epsilon [Cucumis melo] | 301 | 301 | 85% | =HYPERLINK("https://www.ncbi.nlm.nih.gov/protein/XP_008443540.1?report=genbank&log$=prottop&blast_rank=383&RID=EK4FW9P8013","XP_008443540.1") |
| hypothetical protein SORBI_3003G141500 [Sorghum bicolor] | 300 | 300 | 84% | =HYPERLINK("https://www.ncbi.nlm.nih.gov/protein/KXG32344.1?report=genbank&log$=prottop&blast_rank=384&RID=EK4FW9P8013","KXG32344.1") |
| shaggy-related protein kinase eta [Cucumis sativus] | 300 | 300 | 89% | =HYPERLINK("https://www.ncbi.nlm.nih.gov/protein/XP_004134656.1?report=genbank&log$=prottop&blast_rank=385&RID=EK4FW9P8013","XP_004134656.1") |
| shaggy-related protein kinase epsilon [Cucumis sativus] | 300 | 300 | 85% | =HYPERLINK("https://www.ncbi.nlm.nih.gov/protein/XP_004141099.2?report=genbank&log$=prottop&blast_rank=386&RID=EK4FW9P8013","XP_004141099.2") |
| shaggy-related protein kinase eta [Cucumis melo] | 299 | 299 | 89% | =HYPERLINK("https://www.ncbi.nlm.nih.gov/protein/XP_008439730.1?report=genbank&log$=prottop&blast_rank=387&RID=EK4FW9P8013","XP_008439730.1") |
| cyclin-dependent kinase E-1 [Cucumis sativus] | 303 | 303 | 91% | =HYPERLINK("https://www.ncbi.nlm.nih.gov/protein/XP_031739820.1?report=genbank&log$=prottop&blast_rank=388&RID=EK4FW9P8013","XP_031739820.1") |
| shaggy-related protein kinase eta [Lactuca sativa] | 299 | 299 | 89% | =HYPERLINK("https://www.ncbi.nlm.nih.gov/protein/XP_023732896.1?report=genbank&log$=prottop&blast_rank=389&RID=EK4FW9P8013","XP_023732896.1") |
| shaggy-related protein kinase kappa isoform X2 [Cucumis sativus] | 301 | 301 | 85% | =HYPERLINK("https://www.ncbi.nlm.nih.gov/protein/XP_004147888.1?report=genbank&log$=prottop&blast_rank=390&RID=EK4FW9P8013","XP_004147888.1") |
| shaggy-related protein kinase alpha [Sorghum bicolor] | 300 | 300 | 88% | =HYPERLINK("https://www.ncbi.nlm.nih.gov/protein/XP_002440537.1?report=genbank&log$=prottop&blast_rank=391&RID=EK4FW9P8013","XP_002440537.1") |
| unnamed protein product [Lactuca virosa] | 299 | 299 | 89% | =HYPERLINK("https://www.ncbi.nlm.nih.gov/protein/CAH1448573.1?report=genbank&log$=prottop&blast_rank=392&RID=EK4FW9P8013","CAH1448573.1") |
| unnamed protein product [Lactuca saligna] | 298 | 298 | 89% | =HYPERLINK("https://www.ncbi.nlm.nih.gov/protein/CAI9292837.1?report=genbank&log$=prottop&blast_rank=393&RID=EK4FW9P8013","CAI9292837.1") |
| uncharacterized protein LOC8068552 [Sorghum bicolor] | 320 | 320 | 87% | =HYPERLINK("https://www.ncbi.nlm.nih.gov/protein/XP_002444909.2?report=genbank&log$=prottop&blast_rank=394&RID=EK4FW9P8013","XP_002444909.2") |
| putative serine/threonine-protein kinase [Cucumis melo var. makuwa] | 305 | 305 | 91% | =HYPERLINK("https://www.ncbi.nlm.nih.gov/protein/TYK12982.1?report=genbank&log$=prottop&blast_rank=395&RID=EK4FW9P8013","TYK12982.1") |
| shaggy-related protein kinase NtK-1 [Cucumis melo var. makuwa] | 300 | 300 | 85% | =HYPERLINK("https://www.ncbi.nlm.nih.gov/protein/KAA0050959.1?report=genbank&log$=prottop&blast_rank=396&RID=EK4FW9P8013","KAA0050959.1") |
| protein IMPAIRED IN BABA-INDUCED STERILITY 1 [Cucumis sativus] | 308 | 308 | 88% | =HYPERLINK("https://www.ncbi.nlm.nih.gov/protein/XP_004137420.1?report=genbank&log$=prottop&blast_rank=397&RID=EK4FW9P8013","XP_004137420.1") |
| shaggy-related protein kinase kappa [Sorghum bicolor] | 299 | 299 | 89% | =HYPERLINK("https://www.ncbi.nlm.nih.gov/protein/XP_002463546.1?report=genbank&log$=prottop&blast_rank=398&RID=EK4FW9P8013","XP_002463546.1") |
| cyclin-dependent kinase C-2 C [Cucumis melo] | 307 | 307 | 88% | =HYPERLINK("https://www.ncbi.nlm.nih.gov/protein/XP_008459351.1?report=genbank&log$=prottop&blast_rank=399&RID=EK4FW9P8013","XP_008459351.1") |
| hypothetical protein IC582_020683 [Cucumis melo] | 307 | 307 | 88% | =HYPERLINK("https://www.ncbi.nlm.nih.gov/protein/KAL0540673.1?report=genbank&log$=prottop&blast_rank=400&RID=EK4FW9P8013","KAL0540673.1") |
| putative serine/threonine-protein kinase [Cucumis melo var. makuwa] | 310 | 310 | 88% | =HYPERLINK("https://www.ncbi.nlm.nih.gov/protein/KAA0039451.1?report=genbank&log$=prottop&blast_rank=401&RID=EK4FW9P8013","KAA0039451.1") |
| unnamed protein product [Lactuca saligna] | 300 | 300 | 93% | =HYPERLINK("https://www.ncbi.nlm.nih.gov/protein/CAI9263022.1?report=genbank&log$=prottop&blast_rank=402&RID=EK4FW9P8013","CAI9263022.1") |
| glycogen synthase kinase-3 homolog MsK-3 [Cucumis melo] | 298 | 298 | 85% | =HYPERLINK("https://www.ncbi.nlm.nih.gov/protein/XP_050940277.1?report=genbank&log$=prottop&blast_rank=403&RID=EK4FW9P8013","XP_050940277.1") |
| putative serine/threonine-protein kinase [Cucumis melo var. makuwa] | 309 | 309 | 88% | =HYPERLINK("https://www.ncbi.nlm.nih.gov/protein/TYK00641.1?report=genbank&log$=prottop&blast_rank=404&RID=EK4FW9P8013","TYK00641.1") |
| glycogen synthase kinase-3-like protein MsK-3 [Cucumis melo var. makuwa] | 298 | 298 | 85% | =HYPERLINK("https://www.ncbi.nlm.nih.gov/protein/KAA0038106.1?report=genbank&log$=prottop&blast_rank=405&RID=EK4FW9P8013","KAA0038106.1") |
| cyclin-dependent kinase B2-2 isoform X1 [Lactuca sativa] | 295 | 295 | 81% | =HYPERLINK("https://www.ncbi.nlm.nih.gov/protein/XP_042753648.1?report=genbank&log$=prottop&blast_rank=406&RID=EK4FW9P8013","XP_042753648.1") |
| unnamed protein product [Lactuca virosa] | 305 | 305 | 89% | =HYPERLINK("https://www.ncbi.nlm.nih.gov/protein/CAH1428329.1?report=genbank&log$=prottop&blast_rank=407&RID=EK4FW9P8013","CAH1428329.1") |
| unnamed protein product [Lactuca saligna] | 295 | 295 | 80% | =HYPERLINK("https://www.ncbi.nlm.nih.gov/protein/CAI9266930.1?report=genbank&log$=prottop&blast_rank=408&RID=EK4FW9P8013","CAI9266930.1") |
| cyclin-dependent kinase C-2 C-like [Cucumis melo] | 307 | 307 | 91% | =HYPERLINK("https://www.ncbi.nlm.nih.gov/protein/XP_008465829.1?report=genbank&log$=prottop&blast_rank=409&RID=EK4FW9P8013","XP_008465829.1") |
| unnamed protein product [Lactuca saligna] | 302 | 302 | 86% | =HYPERLINK("https://www.ncbi.nlm.nih.gov/protein/CAI9275441.1?report=genbank&log$=prottop&blast_rank=410&RID=EK4FW9P8013","CAI9275441.1") |
| glycogen synthase kinase-3 homolog MsK-3 [Cucumis sativus] | 298 | 298 | 85% | =HYPERLINK("https://www.ncbi.nlm.nih.gov/protein/XP_004148581.1?report=genbank&log$=prottop&blast_rank=411&RID=EK4FW9P8013","XP_004148581.1") |
| hypothetical protein BDA96_04G113700 [Sorghum bicolor] | 298 | 298 | 89% | =HYPERLINK("https://www.ncbi.nlm.nih.gov/protein/KAG0532511.1?report=genbank&log$=prottop&blast_rank=412&RID=EK4FW9P8013","KAG0532511.1") |
| shaggy-related protein kinase eta [Sorghum bicolor] | 298 | 298 | 89% | =HYPERLINK("https://www.ncbi.nlm.nih.gov/protein/XP_002453590.1?report=genbank&log$=prottop&blast_rank=413&RID=EK4FW9P8013","XP_002453590.1") |
| serine/threonine-protein kinase TIO [Cucumis melo var. makuwa] | 296 | 296 | 81% | =HYPERLINK("https://www.ncbi.nlm.nih.gov/protein/TYK20740.1?report=genbank&log$=prottop&blast_rank=414&RID=EK4FW9P8013","TYK20740.1") |
| serine/threonine-protein kinase TIO [Cucumis melo var. makuwa] | 297 | 297 | 81% | =HYPERLINK("https://www.ncbi.nlm.nih.gov/protein/KAA0055120.1?report=genbank&log$=prottop&blast_rank=415&RID=EK4FW9P8013","KAA0055120.1") |
| unnamed protein product [Lactuca saligna] | 292 | 292 | 79% | =HYPERLINK("https://www.ncbi.nlm.nih.gov/protein/CAI9287186.1?report=genbank&log$=prottop&blast_rank=416&RID=EK4FW9P8013","CAI9287186.1") |
| protein IMPAIRED IN BABA-INDUCED STERILITY 1 isoform X2 [Cucumis sativus] | 306 | 306 | 88% | =HYPERLINK("https://www.ncbi.nlm.nih.gov/protein/XP_031741619.1?report=genbank&log$=prottop&blast_rank=417&RID=EK4FW9P8013","XP_031741619.1") |
| shaggy-related protein kinase epsilon isoform X2 [Lactuca sativa] | 296 | 296 | 85% | =HYPERLINK("https://www.ncbi.nlm.nih.gov/protein/XP_023728710.1?report=genbank&log$=prottop&blast_rank=418&RID=EK4FW9P8013","XP_023728710.1") |
| unnamed protein product [Lactuca saligna] | 296 | 296 | 85% | =HYPERLINK("https://www.ncbi.nlm.nih.gov/protein/CAI9272761.1?report=genbank&log$=prottop&blast_rank=419&RID=EK4FW9P8013","CAI9272761.1") |
| hypothetical protein LSAT_V11C100006200 [Lactuca sativa] | 305 | 305 | 89% | =HYPERLINK("https://www.ncbi.nlm.nih.gov/protein/KAJ0228582.1?report=genbank&log$=prottop&blast_rank=420&RID=EK4FW9P8013","KAJ0228582.1") |
| shaggy-related protein kinase eta [Cucumis melo var. makuwa] | 297 | 297 | 89% | =HYPERLINK("https://www.ncbi.nlm.nih.gov/protein/KAA0052613.1?report=genbank&log$=prottop&blast_rank=421&RID=EK4FW9P8013","KAA0052613.1") |
| shaggy-related protein kinase kappa [Lactuca sativa] | 297 | 297 | 86% | =HYPERLINK("https://www.ncbi.nlm.nih.gov/protein/XP_023730280.1?report=genbank&log$=prottop&blast_rank=422&RID=EK4FW9P8013","XP_023730280.1") |
| unnamed protein product [Lactuca saligna] | 304 | 304 | 89% | =HYPERLINK("https://www.ncbi.nlm.nih.gov/protein/CAI9284001.1?report=genbank&log$=prottop&blast_rank=423&RID=EK4FW9P8013","CAI9284001.1") |
| shaggy-related protein kinase epsilon [Lactuca sativa] | 296 | 296 | 85% | =HYPERLINK("https://www.ncbi.nlm.nih.gov/protein/XP_023728705.1?report=genbank&log$=prottop&blast_rank=424&RID=EK4FW9P8013","XP_023728705.1") |
| receptor-like protein kinase FERONIA isoform X1 [Lactuca sativa] | 306 | 471 | 79% | =HYPERLINK("https://www.ncbi.nlm.nih.gov/protein/XP_023750132.1?report=genbank&log$=prottop&blast_rank=425&RID=EK4FW9P8013","XP_023750132.1") |
| probable serine/threonine-protein kinase At1g09600 isoform X3 [Sorghum bicolor] | 303 | 303 | 90% | =HYPERLINK("https://www.ncbi.nlm.nih.gov/protein/XP_021301880.1?report=genbank&log$=prottop&blast_rank=426&RID=EK4FW9P8013","XP_021301880.1") |
| unnamed protein product [Lactuca saligna] | 296 | 296 | 86% | =HYPERLINK("https://www.ncbi.nlm.nih.gov/protein/CAI9287189.1?report=genbank&log$=prottop&blast_rank=427&RID=EK4FW9P8013","CAI9287189.1") |
| shaggy-related protein kinase NtK-1 [Cucumis melo] | 296 | 296 | 85% | =HYPERLINK("https://www.ncbi.nlm.nih.gov/protein/XP_008450520.1?report=genbank&log$=prottop&blast_rank=428&RID=EK4FW9P8013","XP_008450520.1") |
| probable serine/threonine-protein kinase At1g54610 [Lactuca sativa] | 301 | 301 | 86% | =HYPERLINK("https://www.ncbi.nlm.nih.gov/protein/XP_023743669.1?report=genbank&log$=prottop&blast_rank=429&RID=EK4FW9P8013","XP_023743669.1") |
| shaggy-related protein kinase alpha [Cucumis sativus] | 296 | 296 | 85% | =HYPERLINK("https://www.ncbi.nlm.nih.gov/protein/XP_004135570.1?report=genbank&log$=prottop&blast_rank=430&RID=EK4FW9P8013","XP_004135570.1") |
| hypothetical protein LSAT_V11C500246670 [Lactuca sativa] | 302 | 302 | 89% | =HYPERLINK("https://www.ncbi.nlm.nih.gov/protein/KAJ0205020.1?report=genbank&log$=prottop&blast_rank=431&RID=EK4FW9P8013","KAJ0205020.1") |
| shaggy-related protein kinase epsilon isoform X3 [Lactuca sativa] | 294 | 294 | 86% | =HYPERLINK("https://www.ncbi.nlm.nih.gov/protein/XP_023728711.1?report=genbank&log$=prottop&blast_rank=432&RID=EK4FW9P8013","XP_023728711.1") |
| hypothetical protein LSAT_V11C400222050 [Lactuca sativa] | 302 | 302 | 88% | =HYPERLINK("https://www.ncbi.nlm.nih.gov/protein/KAJ0209696.1?report=genbank&log$=prottop&blast_rank=433&RID=EK4FW9P8013","KAJ0209696.1") |
| protein IMPAIRED IN BABA-INDUCED STERILITY 1 [Cucumis sativus] | 306 | 306 | 91% | =HYPERLINK("https://www.ncbi.nlm.nih.gov/protein/XP_004137627.2?report=genbank&log$=prottop&blast_rank=434&RID=EK4FW9P8013","XP_004137627.2") |
| shaggy-related protein kinase GSK1 [Sorghum bicolor] | 295 | 295 | 89% | =HYPERLINK("https://www.ncbi.nlm.nih.gov/protein/XP_002454973.1?report=genbank&log$=prottop&blast_rank=435&RID=EK4FW9P8013","XP_002454973.1") |
| unnamed protein product [Lactuca virosa] | 296 | 296 | 85% | =HYPERLINK("https://www.ncbi.nlm.nih.gov/protein/CAH1443728.1?report=genbank&log$=prottop&blast_rank=436&RID=EK4FW9P8013","CAH1443728.1") |
| shaggy-related protein kinase eta isoform X2 [Lactuca sativa] | 294 | 294 | 89% | =HYPERLINK("https://www.ncbi.nlm.nih.gov/protein/XP_023764137.1?report=genbank&log$=prottop&blast_rank=437&RID=EK4FW9P8013","XP_023764137.1") |
| putative serine/threonine-protein kinase [Cucumis melo var. makuwa] | 306 | 306 | 91% | =HYPERLINK("https://www.ncbi.nlm.nih.gov/protein/TYJ99040.1?report=genbank&log$=prottop&blast_rank=438&RID=EK4FW9P8013","TYJ99040.1") |
| shaggy-related protein kinase theta [Cucumis melo] | 297 | 297 | 81% | =HYPERLINK("https://www.ncbi.nlm.nih.gov/protein/XP_008460173.1?report=genbank&log$=prottop&blast_rank=439&RID=EK4FW9P8013","XP_008460173.1") |
| shaggy-related protein kinase kappa isoform X1 [Lactuca sativa] | 295 | 295 | 85% | =HYPERLINK("https://www.ncbi.nlm.nih.gov/protein/XP_023731228.1?report=genbank&log$=prottop&blast_rank=440&RID=EK4FW9P8013","XP_023731228.1") |
| hypothetical protein Csa_007854 [Cucumis sativus] | 296 | 296 | 78% | =HYPERLINK("https://www.ncbi.nlm.nih.gov/protein/KAE8648933.1?report=genbank&log$=prottop&blast_rank=441&RID=EK4FW9P8013","KAE8648933.1") |
| unnamed protein product [Lactuca saligna] | 301 | 301 | 90% | =HYPERLINK("https://www.ncbi.nlm.nih.gov/protein/CAI9291623.1?report=genbank&log$=prottop&blast_rank=442&RID=EK4FW9P8013","CAI9291623.1") |
| shaggy-related protein kinase kappa isoform X2 [Lactuca sativa] | 295 | 295 | 85% | =HYPERLINK("https://www.ncbi.nlm.nih.gov/protein/XP_023731229.1?report=genbank&log$=prottop&blast_rank=443&RID=EK4FW9P8013","XP_023731229.1") |
| unnamed protein product [Lactuca saligna] | 294 | 294 | 89% | =HYPERLINK("https://www.ncbi.nlm.nih.gov/protein/CAI9269884.1?report=genbank&log$=prottop&blast_rank=444&RID=EK4FW9P8013","CAI9269884.1") |
| protein IMPAIRED IN BABA-INDUCED STERILITY 1 isoform X2 [Lactuca sativa] | 302 | 302 | 89% | =HYPERLINK("https://www.ncbi.nlm.nih.gov/protein/XP_023735381.1?report=genbank&log$=prottop&blast_rank=445&RID=EK4FW9P8013","XP_023735381.1") |
| shaggy-related protein kinase eta isoform X1 [Lactuca sativa] | 293 | 293 | 89% | =HYPERLINK("https://www.ncbi.nlm.nih.gov/protein/XP_023764136.1?report=genbank&log$=prottop&blast_rank=446&RID=EK4FW9P8013","XP_023764136.1") |
| probable serine/threonine-protein kinase At1g54610 [Lactuca sativa] | 300 | 300 | 90% | =HYPERLINK("https://www.ncbi.nlm.nih.gov/protein/XP_023743230.1?report=genbank&log$=prottop&blast_rank=447&RID=EK4FW9P8013","XP_023743230.1") |
| shaggy-related protein kinase theta isoform X1 [Cucumis sativus] | 296 | 296 | 81% | =HYPERLINK("https://www.ncbi.nlm.nih.gov/protein/XP_004144981.1?report=genbank&log$=prottop&blast_rank=448&RID=EK4FW9P8013","XP_004144981.1") |
| cyclin-dependent kinase E-1 isoform X2 [Cucumis melo] | 296 | 296 | 79% | =HYPERLINK("https://www.ncbi.nlm.nih.gov/protein/XP_050945210.1?report=genbank&log$=prottop&blast_rank=449&RID=EK4FW9P8013","XP_050945210.1") |
| hypothetical protein LSAT_V11C300110750 [Lactuca sativa] | 299 | 299 | 89% | =HYPERLINK("https://www.ncbi.nlm.nih.gov/protein/KAJ0215428.1?report=genbank&log$=prottop&blast_rank=450&RID=EK4FW9P8013","KAJ0215428.1") |
| unnamed protein product [Lactuca virosa] | 293 | 293 | 89% | =HYPERLINK("https://www.ncbi.nlm.nih.gov/protein/CAH1440380.1?report=genbank&log$=prottop&blast_rank=451&RID=EK4FW9P8013","CAH1440380.1") |
| protein IMPAIRED IN BABA-INDUCED STERILITY 1 isoform X1 [Lactuca sativa] | 301 | 301 | 89% | =HYPERLINK("https://www.ncbi.nlm.nih.gov/protein/XP_023735380.1?report=genbank&log$=prottop&blast_rank=452&RID=EK4FW9P8013","XP_023735380.1") |
| shaggy-related protein kinase theta [Cucumis melo var. makuwa] | 296 | 296 | 81% | =HYPERLINK("https://www.ncbi.nlm.nih.gov/protein/TYK24466.1?report=genbank&log$=prottop&blast_rank=453&RID=EK4FW9P8013","TYK24466.1") |
| cyclin-dependent kinase E-1 isoform X4 [Cucumis melo] | 293 | 293 | 93% | =HYPERLINK("https://www.ncbi.nlm.nih.gov/protein/XP_050945212.1?report=genbank&log$=prottop&blast_rank=454&RID=EK4FW9P8013","XP_050945212.1") |
| unnamed protein product [Lactuca virosa] | 297 | 297 | 91% | =HYPERLINK("https://www.ncbi.nlm.nih.gov/protein/CAH1427128.1?report=genbank&log$=prottop&blast_rank=455&RID=EK4FW9P8013","CAH1427128.1") |
| hypothetical protein BDA96_01G436600 [Sorghum bicolor] | 288 | 288 | 74% | =HYPERLINK("https://www.ncbi.nlm.nih.gov/protein/KAG0551593.1?report=genbank&log$=prottop&blast_rank=456&RID=EK4FW9P8013","KAG0551593.1") |
| unnamed protein product [Lactuca virosa] | 298 | 298 | 90% | =HYPERLINK("https://www.ncbi.nlm.nih.gov/protein/CAH1454061.1?report=genbank&log$=prottop&blast_rank=457&RID=EK4FW9P8013","CAH1454061.1") |
| hypothetical protein Csa_021669 [Cucumis sativus] | 294 | 294 | 87% | =HYPERLINK("https://www.ncbi.nlm.nih.gov/protein/KAE8649413.1?report=genbank&log$=prottop&blast_rank=458&RID=EK4FW9P8013","KAE8649413.1") |
| hypothetical protein LSAT_V11C300155080 [Lactuca sativa] | 293 | 293 | 91% | =HYPERLINK("https://www.ncbi.nlm.nih.gov/protein/KAJ0215086.1?report=genbank&log$=prottop&blast_rank=459&RID=EK4FW9P8013","KAJ0215086.1") |
| unnamed protein product [Lactuca saligna] | 295 | 295 | 84% | =HYPERLINK("https://www.ncbi.nlm.nih.gov/protein/CAI9272672.1?report=genbank&log$=prottop&blast_rank=460&RID=EK4FW9P8013","CAI9272672.1") |
| shaggy-related protein kinase epsilon [Lactuca sativa] | 294 | 294 | 91% | =HYPERLINK("https://www.ncbi.nlm.nih.gov/protein/XP_023729467.1?report=genbank&log$=prottop&blast_rank=461&RID=EK4FW9P8013","XP_023729467.1") |
| shaggy-related protein kinase alpha [Lactuca sativa] | 292 | 292 | 85% | =HYPERLINK("https://www.ncbi.nlm.nih.gov/protein/XP_023759401.1?report=genbank&log$=prottop&blast_rank=462&RID=EK4FW9P8013","XP_023759401.1") |
| shaggy-related protein kinase alpha isoform X2 [Sorghum bicolor] | 294 | 294 | 85% | =HYPERLINK("https://www.ncbi.nlm.nih.gov/protein/XP_021306685.1?report=genbank&log$=prottop&blast_rank=463&RID=EK4FW9P8013","XP_021306685.1") |
| shaggy-related protein kinase alpha isoform X1 [Sorghum bicolor] | 293 | 293 | 85% | =HYPERLINK("https://www.ncbi.nlm.nih.gov/protein/XP_002465046.1?report=genbank&log$=prottop&blast_rank=464&RID=EK4FW9P8013","XP_002465046.1") |
| SNF1-related protein kinase catalytic subunit alpha KIN10 [Lactuca sativa] | 295 | 295 | 84% | =HYPERLINK("https://www.ncbi.nlm.nih.gov/protein/XP_023771389.1?report=genbank&log$=prottop&blast_rank=465&RID=EK4FW9P8013","XP_023771389.1") |
| cyclin-dependent kinase E-1 [Lactuca sativa] | 294 | 294 | 91% | =HYPERLINK("https://www.ncbi.nlm.nih.gov/protein/XP_023749334.1?report=genbank&log$=prottop&blast_rank=466&RID=EK4FW9P8013","XP_023749334.1") |
| hypothetical protein SORBI_3001G410100 [Sorghum bicolor] | 287 | 287 | 74% | =HYPERLINK("https://www.ncbi.nlm.nih.gov/protein/KXG39625.1?report=genbank&log$=prottop&blast_rank=467&RID=EK4FW9P8013","KXG39625.1") |
| probable serine/threonine-protein kinase At1g54610 [Sorghum bicolor] | 296 | 296 | 90% | =HYPERLINK("https://www.ncbi.nlm.nih.gov/protein/XP_002459013.1?report=genbank&log$=prottop&blast_rank=468&RID=EK4FW9P8013","XP_002459013.1") |
| cyclin-dependent kinase E-1 isoform X5 [Cucumis melo] | 291 | 291 | 80% | =HYPERLINK("https://www.ncbi.nlm.nih.gov/protein/XP_050945213.1?report=genbank&log$=prottop&blast_rank=469&RID=EK4FW9P8013","XP_050945213.1") |
| unnamed protein product [Lactuca saligna] | 293 | 293 | 91% | =HYPERLINK("https://www.ncbi.nlm.nih.gov/protein/CAI9278031.1?report=genbank&log$=prottop&blast_rank=470&RID=EK4FW9P8013","CAI9278031.1") |
| hypothetical protein SORBI_3001G060800 [Sorghum bicolor] | 288 | 288 | 82% | =HYPERLINK("https://www.ncbi.nlm.nih.gov/protein/EER93315.1?report=genbank&log$=prottop&blast_rank=471&RID=EK4FW9P8013","EER93315.1") |
| unnamed protein product [Lactuca virosa] | 291 | 291 | 89% | =HYPERLINK("https://www.ncbi.nlm.nih.gov/protein/CAH1447976.1?report=genbank&log$=prottop&blast_rank=472&RID=EK4FW9P8013","CAH1447976.1") |
| unnamed protein product [Lactuca virosa] | 298 | 298 | 86% | =HYPERLINK("https://www.ncbi.nlm.nih.gov/protein/CAH1414604.1?report=genbank&log$=prottop&blast_rank=473&RID=EK4FW9P8013","CAH1414604.1") |
| shaggy-related protein kinase epsilon isoform X1 [Lactuca sativa] | 291 | 291 | 89% | =HYPERLINK("https://www.ncbi.nlm.nih.gov/protein/XP_023764380.1?report=genbank&log$=prottop&blast_rank=474&RID=EK4FW9P8013","XP_023764380.1") |
| hypothetical protein LSAT_V11C500266230 [Lactuca sativa] | 291 | 291 | 86% | =HYPERLINK("https://www.ncbi.nlm.nih.gov/protein/KAJ0207838.1?report=genbank&log$=prottop&blast_rank=475&RID=EK4FW9P8013","KAJ0207838.1") |
| SNF1-related protein kinase catalytic subunit alpha KIN10 [Lactuca sativa] | 292 | 292 | 94% | =HYPERLINK("https://www.ncbi.nlm.nih.gov/protein/XP_023763673.1?report=genbank&log$=prottop&blast_rank=476&RID=EK4FW9P8013","XP_023763673.1") |
| probable serine/threonine-protein kinase At1g54610 isoform X2 [Cucumis sativus] | 295 | 295 | 86% | =HYPERLINK("https://www.ncbi.nlm.nih.gov/protein/XP_011656469.1?report=genbank&log$=prottop&blast_rank=477&RID=EK4FW9P8013","XP_011656469.1") |
| probable serine/threonine-protein kinase At1g54610 isoform X1 [Cucumis sativus] | 295 | 295 | 86% | =HYPERLINK("https://www.ncbi.nlm.nih.gov/protein/XP_004138365.1?report=genbank&log$=prottop&blast_rank=478&RID=EK4FW9P8013","XP_004138365.1") |
| putative cyclin-dependent kinase F-2 [Sorghum bicolor] | 288 | 288 | 82% | =HYPERLINK("https://www.ncbi.nlm.nih.gov/protein/XP_002466317.2?report=genbank&log$=prottop&blast_rank=479&RID=EK4FW9P8013","XP_002466317.2") |
| shaggy-related protein kinase eta [Cucumis melo var. makuwa] | 288 | 288 | 89% | =HYPERLINK("https://www.ncbi.nlm.nih.gov/protein/TYK13215.1?report=genbank&log$=prottop&blast_rank=480&RID=EK4FW9P8013","TYK13215.1") |
| shaggy-related protein kinase theta isoform X2 [Lactuca sativa] | 289 | 289 | 89% | =HYPERLINK("https://www.ncbi.nlm.nih.gov/protein/XP_023764381.1?report=genbank&log$=prottop&blast_rank=481&RID=EK4FW9P8013","XP_023764381.1") |
| unnamed protein product [Lactuca saligna] | 292 | 292 | 91% | =HYPERLINK("https://www.ncbi.nlm.nih.gov/protein/CAI9285171.1?report=genbank&log$=prottop&blast_rank=482&RID=EK4FW9P8013","CAI9285171.1") |
| hypothetical protein SORBI_3001G408200 [Sorghum bicolor] | 287 | 287 | 81% | =HYPERLINK("https://www.ncbi.nlm.nih.gov/protein/KXG39614.1?report=genbank&log$=prottop&blast_rank=483&RID=EK4FW9P8013","KXG39614.1") |
| unnamed protein product [Lactuca virosa] | 291 | 291 | 94% | =HYPERLINK("https://www.ncbi.nlm.nih.gov/protein/CAH1428332.1?report=genbank&log$=prottop&blast_rank=484&RID=EK4FW9P8013","CAH1428332.1") |
| unnamed protein product [Lactuca virosa] | 288 | 288 | 85% | =HYPERLINK("https://www.ncbi.nlm.nih.gov/protein/CAH1434206.1?report=genbank&log$=prottop&blast_rank=485&RID=EK4FW9P8013","CAH1434206.1") |
| hypothetical protein BDA96_08G070500 [Sorghum bicolor] | 286 | 286 | 79% | =HYPERLINK("https://www.ncbi.nlm.nih.gov/protein/KAG0520396.1?report=genbank&log$=prottop&blast_rank=486&RID=EK4FW9P8013","KAG0520396.1") |
| unnamed protein product [Lactuca virosa] | 294 | 294 | 85% | =HYPERLINK("https://www.ncbi.nlm.nih.gov/protein/CAH1429857.1?report=genbank&log$=prottop&blast_rank=487&RID=EK4FW9P8013","CAH1429857.1") |
| putative serine/threonine-protein kinase [Cucumis melo var. makuwa] | 293 | 293 | 88% | =HYPERLINK("https://www.ncbi.nlm.nih.gov/protein/KAA0039176.1?report=genbank&log$=prottop&blast_rank=488&RID=EK4FW9P8013","KAA0039176.1") |
| serine/threonine protein kinase OSK1 [Sorghum bicolor] | 291 | 291 | 80% | =HYPERLINK("https://www.ncbi.nlm.nih.gov/protein/XP_002441429.1?report=genbank&log$=prottop&blast_rank=489&RID=EK4FW9P8013","XP_002441429.1") |
| probable serine/threonine-protein kinase At1g54610 [Cucumis melo] | 293 | 293 | 88% | =HYPERLINK("https://www.ncbi.nlm.nih.gov/protein/XP_008463051.2?report=genbank&log$=prottop&blast_rank=490&RID=EK4FW9P8013","XP_008463051.2") |
| serine/threonine-protein kinase TIO [Lactuca sativa] | 306 | 306 | 80% | =HYPERLINK("https://www.ncbi.nlm.nih.gov/protein/XP_023731866.1?report=genbank&log$=prottop&blast_rank=491&RID=EK4FW9P8013","XP_023731866.1") |
| hypothetical protein LSAT_V11C600338920 [Lactuca sativa] | 306 | 306 | 80% | =HYPERLINK("https://www.ncbi.nlm.nih.gov/protein/KAJ0199998.1?report=genbank&log$=prottop&blast_rank=492&RID=EK4FW9P8013","KAJ0199998.1") |
| hypothetical protein IC582_024034 [Cucumis melo] | 296 | 296 | 88% | =HYPERLINK("https://www.ncbi.nlm.nih.gov/protein/KAL0539813.1?report=genbank&log$=prottop&blast_rank=493&RID=EK4FW9P8013","KAL0539813.1") |
| serine/threonine protein kinase OSK1 [Sorghum bicolor] | 289 | 289 | 84% | =HYPERLINK("https://www.ncbi.nlm.nih.gov/protein/XP_002456286.1?report=genbank&log$=prottop&blast_rank=494&RID=EK4FW9P8013","XP_002456286.1") |
| putative cyclin-dependent kinase F-2 [Sorghum bicolor] | 284 | 284 | 79% | =HYPERLINK("https://www.ncbi.nlm.nih.gov/protein/XP_002442963.1?report=genbank&log$=prottop&blast_rank=495&RID=EK4FW9P8013","XP_002442963.1") |
| SNF1-related protein kinase catalytic subunit alpha KIN10 [Cucumis melo] | 290 | 290 | 83% | =HYPERLINK("https://www.ncbi.nlm.nih.gov/protein/XP_008460110.1?report=genbank&log$=prottop&blast_rank=496&RID=EK4FW9P8013","XP_008460110.1") |
| SNF1-related protein kinase catalytic subunit alpha KIN10 isoform X1 [Cucumis sativus] | 290 | 290 | 83% | =HYPERLINK("https://www.ncbi.nlm.nih.gov/protein/XP_011656702.1?report=genbank&log$=prottop&blast_rank=497&RID=EK4FW9P8013","XP_011656702.1") |
| unnamed protein product [Lactuca saligna] | 291 | 291 | 94% | =HYPERLINK("https://www.ncbi.nlm.nih.gov/protein/CAI9283994.1?report=genbank&log$=prottop&blast_rank=498&RID=EK4FW9P8013","CAI9283994.1") |
| hypothetical protein LSAT_V11C500236640 [Lactuca sativa] | 293 | 293 | 89% | =HYPERLINK("https://www.ncbi.nlm.nih.gov/protein/KAJ0206033.1?report=genbank&log$=prottop&blast_rank=499&RID=EK4FW9P8013","KAJ0206033.1") |
| SNF1-related protein kinase catalytic subunit alpha KIN10 [Cucumis melo var. makuwa] | 291 | 291 | 83% | =HYPERLINK("https://www.ncbi.nlm.nih.gov/protein/KAA0039992.1?report=genbank&log$=prottop&blast_rank=500&RID=EK4FW9P8013","KAA0039992.1") |
